# Parameter estimation in large-scale systems biology models: a parallel and self-adaptive cooperative strategy

**DOI:** 10.1186/s12859-016-1452-4

**Published:** 2017-01-21

**Authors:** David R. Penas, Patricia González, Jose A. Egea, Ramón Doallo, Julio R. Banga

**Affiliations:** 1 BioProcess Engineering Group, IIM-CSIC, Eduardo Cabello 6, Vigo, 36208 Spain; 2 Computer Architecture Group, Universidade da Coruña, Campus de Elviña s/n, Coruña, 15071 A Spain; 3 Department of Applied Mathematics and Statistics, Universidad Politécnica de Cartagena, c/ Dr. Fleming s/n, Cartagena, 30202 Spain

**Keywords:** Dynamic models, Parameter estimation, Global optimization, Metaheuristics, Parallelization

## Abstract

**Background:**

The development of large-scale kinetic models is one of the current key issues in computational systems biology and bioinformatics. Here we consider the problem of parameter estimation in nonlinear dynamic models. Global optimization methods can be used to solve this type of problems but the associated computational cost is very large. Moreover, many of these methods need the tuning of a number of adjustable search parameters, requiring a number of initial exploratory runs and therefore further increasing the computation times.

Here we present a novel parallel method, self-adaptive cooperative enhanced scatter search (saCeSS), to accelerate the solution of this class of problems. The method is based on the scatter search optimization metaheuristic and incorporates several key new mechanisms: (i) asynchronous cooperation between parallel processes, (ii) coarse and fine-grained parallelism, and (iii) self-tuning strategies.

**Results:**

The performance and robustness of saCeSS is illustrated by solving a set of challenging parameter estimation problems, including medium and large-scale kinetic models of the bacterium *E. coli*, bakerés yeast *S. cerevisiae*, the vinegar fly *D. melanogaster*, Chinese Hamster Ovary cells, and a generic signal transduction network.

The results consistently show that saCeSS is a robust and efficient method, allowing very significant reduction of computation times with respect to several previous state of the art methods (from days to minutes, in several cases) even when only a small number of processors is used.

**Conclusions:**

The new parallel cooperative method presented here allows the solution of medium and large scale parameter estimation problems in reasonable computation times and with small hardware requirements. Further, the method includes self-tuning mechanisms which facilitate its use by non-experts. We believe that this new method can play a key role in the development of large-scale and even whole-cell dynamic models.

**Electronic supplementary material:**

The online version of this article (doi:10.1186/s12859-016-1452-4) contains supplementary material, which is available to authorized users.

## Background

Computational simulation and optimization are key topics in systems biology and bioinformatics, playing a central role in mathematical approaches considering the reverse engineering of biological systems [1–9] and the handling of uncertainty in that context [10–14]. Due to the significant computational cost associated with the simulation, calibration and analysis of models of realistic size, several authors have considered different parallelization strategies in order to accelerate those tasks [15–18].

Recent efforts have been focused on scaling-up the development of dynamic (kinetic) models [19–25], with the ultimate goal of obtaining whole-cell models [26, 27]. In this context, the problem of parameter estimation in dynamic models (also known as model calibration) has received great attention [28–30], particularly regarding the use of global optimization metaheuristics and hybrid methods [31–35]. It should be noted that the use of multi-start local methods (i.e. repeated local searches started from different initial guesses inside a bounded domain) also enjoys great popularity, but it has been shown to be rather inefficient, even when exploiting high-quality gradient information [35]. Parallel global optimization strategies have been considered in several system biology studies, including parallel variants of simulated annealing [36], evolution strategies [37–40], particle swarm optimization [41, 42] and differential evolution [43].

Scatter search is a promising metaheuristic that in sequential implementations has been shown to outperform other state of the art stochastic global optimization methods [35, 44–50]. Recently, a prototype of cooperative scatter search implementation using multiple processors was presented [51], showing good performance for the calibration of several large-scale models. However, this prototype used a simple synchronous strategy and small number of processors (due to inefficient communications). Thus, although it could reduce the computation times of sequential scatter search, it still required very significant efforts when dealing with large-scale applications.

Here we significantly extend and improve this method by proposing a new parallel cooperative scheme, named self-adaptive cooperative enhanced scatter search (saCeSS) that incorporates the following novel strategies:

- the combination of a coarse-grained distributed-memory parallelization paradigm and an underlying fine-grained parallelization of the individual tasks with a shared-memory model, in order to improve the scalability.
- an improved cooperation scheme, including an information exchange mechanism driven by the quality of the solutions, an asynchronous communication protocol to handle inter-process information exchange, and a self-adaptive procedure to dynamically tune the settings of the parallel searches.

We present below a detailed description of saCeSS, including the details of a high-performance implementation based on a hybrid message passing interface (MPI) and open multi-processing (OpenMP) combination. The excellent performance and scalability of this novel method are illustrated considering a set of very challenging parameter estimation problems in large-scale dynamic models of biological systems. These problems consider kinetic models of the bacterium *E. coli*, bakerés yeast *S. cerevisiae*, the vinegar fly *D. melanogaster*, Chinese Hamster Ovary cells and a generic signal transduction network. The results consistently show that saCeSS is a robust and efficient method, allowing a very significant reduction of computation times with respect to previous methods (from days to minutes, in several cases) even when only a small number of processors is used. Therefore, we believe that this new method can play a key role in the development of large-scale dynamic models in systems biology.

## Methods

### Problem statement

Here we consider the problem of parameter estimation in dynamic models described by deterministic nonlinear ordinary differential equation models. However, it should be noted that the method described below is applicable to other model classes.

Given such a model and a measurements data set (observations of some of the dynamic states, given as time-series), the objective of parameter estimation is to find the optimal vector **p** (unknown model parameters) that minimizes the mismatch between model predictions and the measurements. Such a mismatch is given by a cost function, i.e. a scalar function that quantifies the model error (typically, a least-squares or maximum likelihood form).

The mathematical statement is therefore a nonlinear programming (NLP) problem with differential-algebraic constraints (DAEs). Assuming a generalized least squares cost function, the problem is:

Find **p** to minimize

1 $$ J = \sum\limits_{\varepsilon=1}^{n_{\varepsilon}} \sum\limits_{o=1}^{n_{o}^{\varepsilon}} \sum\limits_{s=1}^{n_{s}^{\varepsilon,o}} (ym_{s}^{\varepsilon,o} - y_{s}^{\varepsilon,o}(\mathbf{p}))^{T} W(ym_{s}^{\varepsilon,o} - y_{s}^{\varepsilon,o}(\mathbf{p}))  $$

where *n*_*ε*_ is the number of experiments, $n_{o}^{\epsilon }$ is the number of the observables which represent the state variables measured experimentally, $ym_{s}^{\varepsilon,o}$ corresponds with the measured data, $n_{s}^{\epsilon,o}$ is the number of the samples per observable per experiment, $y_{s}^{\varepsilon,o}(\mathbf {p})$ are the model predictions and *W* is a scaling matrix that balances the residuals.

In addition, the optimization above is subject to a number of constraints:

2 $$ \dot{\mathbf{x}} = f(x,\mathbf{p},t)  $$

3 $$ \mathbf{x}(t_{o}) = x_{o}  $$

4 $$ \mathbf{y} = g(x,\mathbf{p},t)  $$

5 $$ \mathbf{h}_{eq}(x,y,\mathbf{p}) = 0  $$

6 $$ \mathbf{h}_{in}(x,y,\mathbf{p}) \leq 0  $$

7 $$ \mathbf{p}^{L} \leq \mathbf{p} \leq \mathbf{p}^{U}  $$

where *f* is the nonlinear dynamic problem with the differential-algebraic constraints (DAEs), *x* is the vector of state variables and *x*_*o*_ are their initial conditions; *g* is the observation function that gives the predicted observed states (**y** is mapped to *y*_*s*_ in Eq. 1); *h*_*eq*_ and *h*_*in*_ are equality and inequality constraints; and *p* ^*L*^ and *p* ^*U*^ are upper and lower bounds for the decision vector **p**.

Due to the non-convexity of the parameter estimation problem above, suitable global optimization must be used [31, 33, 35, 52–54]. Previous studies have shown that the scatter search metaheuristic is a very competitive method for this class of problems [35, 44, 45].

### Scatter search

Scatter search (SS) [55] is a population based metaheuristic for global optimization that constructs new solutions based on systematic combinations of the members of a reference set (called *RefSet* in this context). The *RefSet* is the analogous concept to the *population* in genetic or evolutionary algorithms but its size is considerably smaller than in those methods. A consequence is that the degree of randomness in scatter search is lower than in other population based metaheuristic and the generation of new solutions is based on the combination of the *RefSet* members. Another difference between scatter search and other classical population based methods is the use of the *improvement method* which usually consists of local searches from selected solutions to accelerate the convergence to the optimum in certain problems, turning the algorithm into a more effective combination of global and local search. This *improvement method* can of course be ignored in those problems where local searches are very time-consuming and/or inefficient.

**Fig. 1:**
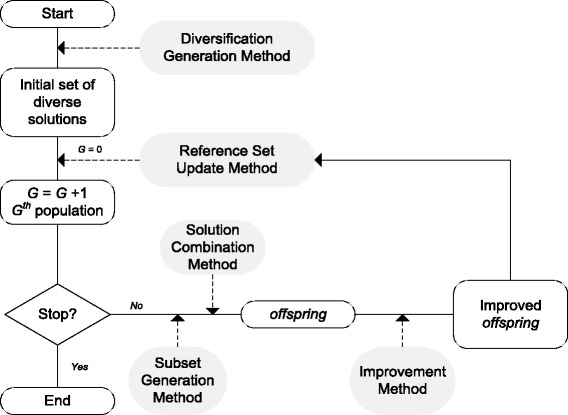
Schematic representation of a basic Scatter Search algorithm

Figure 1 shows a schematic representation of a basic Scatter Search algorithm where the steps of the popular *five-step template* [56] are highlighted. Classical scatter search implementations update the *RefSet* by replacing the worst elements with new ones which outperform their quality. In continuous optimization, as is the case of the problems considered in the present study, this can lead to premature stagnation and lack of diversity among the *RefSet* members. The scatter search version used in this work as a starting point is based on a recent implementation [45, 57], named *enhanced scatter search* (eSS), in which the population update is carried out in a different way so as to avoid stagnation problems and increase the diversity of the search without losing efficiency.

Basic pseudocodes of the eSS algorithm are shown in Algorithms 1 (main routine) and 2 (local search). The method begins by creating and evaluating an initial set of *ndiverse* random solutions within the search space (line 4). Then, the *RefSet* is generated using the best solutions and random solutions from the initial set (line 6). When all data is initialized and the first *RefSet* is created, the eSS repeats the main loop until the stopping criterion is fulfilled.

These main steps of the algorithm are briefly described in the following lines:

1. *RefSet* order and duplicity check: The members of the *RefSet* are sorted by quality. After that, if two (or more) *RefSet* members are too close to one another, one (or more) will automatically be replaced by random solutions (lines 8-12). These comparisons are performed pair to pair for all members of the *Refset*, considering normalized solutions: every solution vector is normalized in the interval [0, 1] based on the upper and lower bounds. Thus, two solutions are “too close” to each other if the maximum difference of its components is higher than a given threshold, with a default value of 1e-3. This mechanism contributes to increase the diversity in the *RefSet* thus preventing the search from stagnation.
2. Solution combination: This step consists in pair-wise combinations of the *RefSet* members (lines 13-23). The new solutions resulting from the combinations are generated in hyper-rectangles defined by the relative position and distance of the *RefSet* members being combined. This is accomplished by doing linear combinations in every dimension of the solutions, weighted by a random factor and bounded by the relative distance of the combined solutions. More details about this type of combination can be found in [57].
3. *RefSet* update: The solutions generated by combination can replace the *RefSet* members if they outperform their quality (line 53). In order to preserve the *RefSet* diversity and avoid premature stagnation, a (1+ *λ*)[58] evolution strategy is implemented in this step. This means that a new solution can only replace that *RefSet* member that defined the hyper-rectangle where the new solution was created. In other words, a solution can only replace its “parent”. What is more, among all the solutions generated in the same hyper-rectangle, only the best of them will replace the “parent”. This mechanism avoids clusters of similar solutions in early stages of the search which could produce premature stagnation.
4. Extra mechanisms: eSS includes two procedures to make the search more efficient. One is the so-called “go-beyond” strategy and consists in exploiting promising search directions. If a new solution outperforms its “parent”, a new hyper-rectangle following the direction of both solutions and beyond the line linking them is created. A new solution is created in this new hyper-rectangle, and the process is repeated varying the hyper-rectangle size as long as there is improvement (lines 24-49). The second mechanism consists in a stagnation checking. If a *RefSet* solution has not been updated during a predefined number of iterations, we consider that it is a local solution and replace it with a new random solution in the *RefSet*. This is carried out by using a counter (*n*_*stuck*_) for each *RefSet* member (lines 54-59).
5. Improvement method. This is basically a local search procedure that is implemented in the following form (see Algorithm 2): when the local search is activated, we distinguish between the first local search (which is carried out from the best found solution after *local.n1* function evaluations), and the rest. Once the first local search has been performed, the next ones take place after *local.n2* function evaluations from the previous local search. In this case, the initial point is chosen from the new solutions created by combination in the previous step, balancing between their quality and diversity. The diversity is computed measuring the distance between each solution and all the previous local solutions found. The parameter *balance* gives more weight to the quality or to the diversity when choosing a candidate as the initial point for the local search. Once a new local solution is found, it is added to a list. There is an exception when the *best*_*sol* parameter is activated. In this case, the local search will only be applied over the best found solution as long as it has been updated in the incumbent iteration. Based on our previous experience, this strategy is only useful in certain pathological problems, and should not be activated by default.

For further details on the eSS implementation, the reader is referred to [45, 57].

### Parallelization of enhanced Scatter Search

The parallelization of metaheuristics pursues one or more of the following goals: to increase the size of the problems that can be solved, to speed-up the computations, or to attempt a more thorough exploration of the solution space [59, 60]. However, achieving an efficient parallelization of a metaheuristic is usually a complex task since the search of new solutions depends on previous iterations of the algorithm, which not only complicates the parallelization itself but also limits the achievable speedup. Different strategies can be used to address this problem: (i) attempting to find parallelism in the sequential algorithms and preserving their behavior; (ii) finding parallel variants of the sequential algorithms and slightly varying their behavior to obtain a more easily parallelizable algorithm; or (iii) developing fully decoupled algorithms, where each process executes its part without communication with other processes, at the expense of reducing its effectiveness.

In the case of the enhanced scatter search method, finding parallelism in the sequential algorithm is straightforward: the majority of time-consuming operations (evaluations of the cost function) are located in inner loops (e.g. lines 13-23 in Algorithm 1) which can be easily performed in parallel. However, since the main loop of the algorithm (line 7 in Algorithm 1) presents dependencies between different iterations and the dimension of the combination loop is rather small, a fine-grained parallelization would limit the scalability in distributed systems. Thus, a more effective solution is a coarse-grained parallelization that implies finding a parallel variant of the sequential algorithm. An island-model approach [61] can be used, so that the reference set is divided into subsets (*islands*) where the eSS is executed isolated and sparse individual exchanges are performed among islands to link different subsets. This solution drastically reduces the communications between distributed processes. However, its scalability is again heavily restrained by the small size of the reference set in the eSS method. Reducing the already small reference set by dividing it between the different islands will have a negative impact on the convergence of the eSS. Thus, building upon the ideas outlined in [51], here we propose an island-based method where each island performs an eSS using a different *RefSet*, while they cooperate modifying the systemic properties of the individual searches.





Current High Performance Computing (HPC) systems include clusters of multicore nodes that can benefit from the use of a hybrid programming model, in which a message passing library, such as MPI (Message Passing Interface), is used for the inter-node communications while a shared memory programming model, such as OpenMP, is used intra-node. Even though programming using a hybrid MPI+OpenMP model requires some effort from application developers, this model provides several advantages such as reducing the communication needs and memory consumption, as well as improving load balance and numerical convergence [62].

**Fig. 2:**
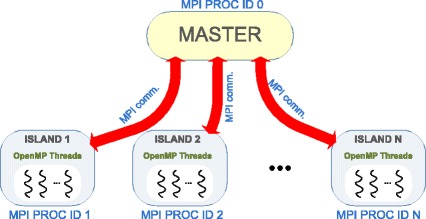
Schematic representation of the proposed hybrid MPI+OpenMP algorithm. Each MPI process is an island that performs an isolated eSS. Cooperation between islands is achieved through the master process by means of message passing. Each MPI process (island) spawns multiple OpenMP threads to perform the evaluations within its population in parallel

Thus, the combination of a coarse-grained parallelization using a distributed-memory paradigm and an underlying fine-grained parallelization of the individual tasks with a shared-memory model is an attractive solution for improving the scalability of the proposal. A hybrid implementation combining MPI and OpenMP is explored in this work. The proposed solution pursues the development of an efficient cooperative enhanced Scatter Search, focused on both the acceleration of the computation by performing separate evaluations in parallel and the convergence improvement through the stimulation of the diversification in the search and the cooperation between different islands. MPI is used for communication between different islands, that is, for the cooperation itself, while OpenMP is used inside each island to accelerate the computation of the evaluations. Figure 2 schematically illustrates this idea.

### Fine-grained parallelization

The most time consuming task in the eSS algorithm is the evaluation of the solutions (cost function values corresponding to new vectors in the parameter space). This task appears in several steps of the algorithm, such as in the creation of the initial random *ndiverse* solutions, in the combination loop to generate new solutions, and in the go-beyond method (lines 4, 13-23 and 24-49 in Algorithm 1, respectively). Thus, we have decided to perform all these evaluations in parallel using the OpenMP library.

Algorithm 3 shows a basic pseudocode for performing the solutions’ evaluation in parallel. As can be observed, every time an evaluation of the solutions is needed, a parallel loop is defined. In OpenMP, the execution of a parallel loop is based on the fork-join programming model. In the parallel section, the thread running creates a group of threads, so that the set of solutions to be evaluated are divided among them and each evaluation is performed in parallel. At the end of the parallel loop, the different threads are synchronized and finally joined again into only one thread. Due to this synchronization, load imbalance in the parallel loop can cause significant delays. This is the case of the evaluations in the eSS, since different evaluations can have entirely different computational loads. Thus, a dynamic schedule clause must be used so that the assignment can vary at run-time and the iterations are handed out to threads as they complete their previously assigned evaluation. Finally, at the end of the parallel region, a reduction operation allows for counting the number of total evaluations performed.



### Coarse-grained parallelization

The coarse-grained parallelization proposed is based on the cooperation between parallel processes (*islands*). For this cooperation to be efficient in large-scale difficult problems, each island must adopt a different strategy to increase the diversification in the search. The idea is to run in parallel processes with different degrees of *aggressiveness*. Some processes will focus on diversification (global search) increasing the probabilities of finding a feasible solution even in a *rough* or difficult space. Other processes will concentrate on intensification (local search) and speed-up the computations in *smoother* spaces. Cooperation among them enables each process to benefit from the knowledge gathered by the rest. However, an important issue to be solved in parallel cooperative schemes is the coordination between islands so that the processes’ stalls due to synchronizations are minimized in order to improve the efficiency and, specifically, the scalability of the parallel approach.

The solution proposed in this work follows a popular centralized master-slave approach. However, as opposed to most master-slave approaches, in the proposed solution the master process does not play the role of a central globally accessible memory. The data is completely distributed among the slaves (islands) that perform a sequential eSS each. The master process is in charge of the cooperation between the islands. The main features of the proposal scheme presented in this work are:

- *cooperation between islands*: by means of the exchange of information driven by the quality of the solutions obtained in each slave, rather than by elapsed time, to achieve more effective cooperation between processes.
- *asynchronous communication protocol*: to handle inter-process information exchange, avoiding idle processes while waiting for information exchanged from other processes.
- *self-adaptive procedure*: to dynamically change the settings of those slaves that do not cooperate, sending to them the settings of the most promising processes.

In the following subsections we describe in detail the implementation of the new self-adaptive cooperative enhanced Scatter Search algorithm (saCeSS), focusing on these three main features and providing evidences for each of the design decisions taken.

#### Cooperation between islands

Some fundamental issues have to be addressed when designing cooperative parallel strategies [63], such as what information is exchanged, between which processes it is exchanged, when and how information is exchanged and how the imported information is used. The solution to these issues has to be carefully designed to avoid well-documented adverse impacts on diversity that may lead to premature convergence.

The cooperative search strategy proposed in this paper accelerates the exploration of the search space through different mechanisms: launching simultaneous searches with different configurations from independent initial points and including cooperation mechanisms to share information between processes. The exchange of information among cooperating search processes is driven by the quality of the solutions obtained. *Promising* solutions obtained in each island are sent to the master process to be spread to the rest of the islands.

On the one hand, a key aspect of the cooperation scheme is deciding when a solution is considered *promising*. The accumulated knowledge of the field indicates that information exchange between islands should not be too frequent to avoid premature convergence to local optima [64, 65]. Thus, exchanging all current-best solutions is avoided to prevent the cooperation entries from filling up the islands’ populations and leading to a rapid decrease of the diversity. Instead, a threshold is used to determine when a new best solution outperforms significantly the current-best solution and it deserves to be spread to the rest. The threshold selection adds to a new degree of freedom that needs to be fixed to the cooperative scheme. The adaptive procedure described further in this section solves this issue.

On the other hand, the strategy used to select those members of the *RefSet* to be replaced with the incoming solutions, that is, with *promising* solutions from other islands, should be carefully decided. One of the most popular selection/replacement policies for incoming solutions in parallel metaheuristics is to replace the *worst* solution in the current population with the incoming solution when the value of the latter is better than that of the former. However, this policy is contrary to the *RefSet* update strategy used in the eSS method, going against the idea that *parents* can only be replaced by their own *children* to avoid loss of diversity and to prevent premature stagnation. Since an incoming solution is always a promising one, replacing the *worst* solution will promote this entry to higher positions in the sorted *RefSet*. It is easy to realise that, after a few iterations receiving new *best* solutions, considering the small *RefSet* in the eSS method, the initial population in each island will be lost and the *RefSet* will be full of foreign individuals. Moreover, all the island populations would tend to be uniform, thus, losing diversity and potentially leading to rapidly converge to suboptimal solutions. Replacing the *best* solution instead of the *worst* one solves this issue most of the times. However, several members of the initial population could still be replaced by foreign solutions. Thus, the selection/replacement policy proposed in this work consists in labeling one member of the *RefSet* as a cooperative member, so that a foreign solution can only enter the population by replacing this cooperative solution. The first time a shared solution is received, the *worst* solution in the *RefSet* will be replaced. This solution will be labeled as a *cooperative* solution for the next iterations. A *cooperative* solution is handled like any other solution in the *RefSet*, being combined and improved following the eSS algorithm. It can also be updated by being replaced by its own offspring solutions. Restricting the replacement of foreign solutions to the *cooperative* entry, the algorithm will evolve over the initial population and still *promising* solutions from other islands may benefit the search in the next iterations.

As described before, the eSS method already includes a stagnation checking mechanism (lines 54–59 in Algorithm 1) to replace those solutions of the population that which cannot be improved in a certain number of iterations of the algorithm by random generated solutions. Thus, diversity is automatically introduced in the eSS when the members in the *RefSet* appeared to be stuck. In the cooperative scheme this strategy may punish the *cooperative* solution by replacing it too early. In order to avoid that, a *nstuck* larger than that of other members of the RefSet is assigned to the *cooperative* solution.

#### Asynchronous communication protocol

An important aspect when designing the communication protocol is the interconnection topology of the different components of the parallel algorithm. A widely used topology in master-slave models, the *star* topology, is used in this work, since it enables different components of the parallel algorithm to be tightly coupled, thus quickly spreading the solutions to improve the convergence. The master process is in the center of the star and all the rest of the processes (slaves) exchange information through the master. The distance between any two slaves is always two, therefore it avoids communication delays that would harm the cooperation between processes.

**Fig. 3:**
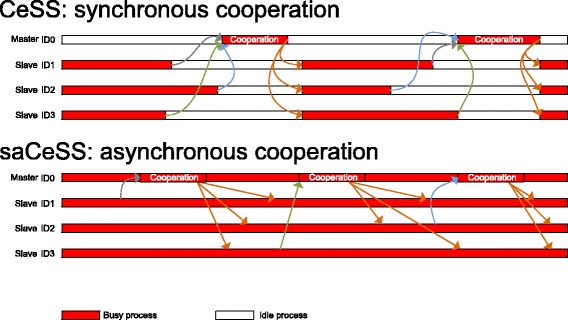
Visualization of performance analysis against time comparing synchronous versus asynchronous cooperation schemes

The communication protocol is designed to avoid processes’ stalls if messages have not arrived during an external iteration, allowing for the progress of the execution in every individual process. Both the emission and reception of the messages are performed using non-blocking operations, thus allowing for the overlap of communications and computations. This is crucial in the application of the saCeSS method to solve large-scale difficult problems since the algorithm success heavily depends on the diversification degree introduced in the different islands that would result in an asynchronous running of the processes and a computationally unbalanced scenario. Figure 3 illustrates this fact by comparing a synchronous cooperation scheme with the asynchronous cooperation proposed here. In a synchronous scheme, all the processes need to be synchronized during the cooperation stage, while in the proposal, each process communicates its promising results and receives the cooperative solutions to/from the master in an asynchronous fashion, avoiding idle periods.

#### Self-adaptive procedure

The adaptive procedure aims to dynamically change, during the search process, several parameters that impact the success of the parallel cooperative scheme. In the proposed solution, the master process controls the long-term behavior of the parallel searches and their cooperation. An iterative life cycle model has been followed for the design and implementation of the tuning procedure and several parameter estimation benchmarks have been used for the evaluation of the proposal in each iteration, in order to refine the solution to tune widespread problems.

**Fig. 4:**
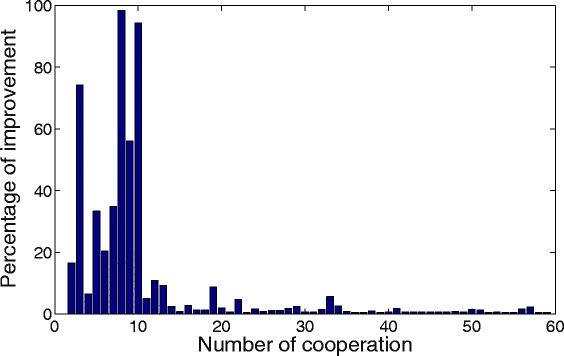
Improvement as a function of cooperation. Percentage of improvement of a new best solution with respect to the previous best known solution, as a function of the number of cooperation events. Results obtained from benchmark B4, as reported in the Results and discussion section

First, the master process is in charge of the threshold selection used to decide which cooperative solutions that arrive at the master are qualified to be spread to the island. If the threshold is too large, cooperation will happen only sporadically, and its efficiency will be reduced. However, if the threshold is too small, the number of communications will increase, which not only negatively affects the efficiency of the parallel implementation, but also is often counterproductive since solutions are generally similar, and the receiver processes have no chance of actually acting on the incoming information. It has also been observed that excess cooperation may rapidly decrease the diversity of the parts of the search space explored (many islands will search in the same region) and bring an early convergence to a non-optimal solution. For illustrative purposes Fig. 4 shows the percentage of improvement of each spread solution when using a very low fixed threshold. Considering that at the beginning of the execution the improvements in the local solutions will be notably larger than at the end, an adaptive procedure that allows to start with a large threshold and decrease it with the search progress will improve the efficiency of the cooperation scheme. The suggested threshold to begin with is a 10%, that is, incoming solutions that improve the best known solution in the master process by at least 10% are spread to the islands as cooperative solutions. Once the search progresses and most of the incoming solutions are below this threshold of improvement, the master reduces the threshold to a half. This procedure is repeated, so that the threshold is reduced, driven by the incoming solutions (i.e., the search progress in the islands). Note that if a excessively high threshold is selected, it will rapidly decrease to an adequate value for the problem at hand, when the master process ascertains that there are insufficient incoming solutions below this threshold.

Second, the master process is used as a scoreboard intended to dynamically tune the settings of the eSS in the different islands. As commented above, each island in the proposed scheme performs a different eSS. An *aggressive* island performs frequent local searches, trying to refine the solution very quickly and keeps a small reference set of solutions. It will perform well in problems with parameter spaces that have a smooth shape. On the other hand, *conservative* islands have a large reference set and perform local searches only sporadically. They spend more time combining parameter vectors and exploring the different regions of the parameter space. Thus, they are more appropriate for problems with rugged parameter spaces. Since the exact nature of the problem at hand is always unknown, it is recommended to choose, at the beginning of the scheme, a range of settings that yields conservative, aggressive, and intermediate islands. However, a procedure that adaptively changes the settings in the islands during the execution, favoring those settings that exhibit the highest success, will further improve the efficiency of the evolutionary search.

There are several configurable settings that determine the strategy (conservative/aggressive) used by the sequential eSS algorithm, and whose selection may have a great impact in the algorithm performance. Namely, these settings are:

- Number of elements in the reference set (*dimRefSet*, defined in line 1 in Algorithm 1).
- Minimum number of iterations of the eSS algorithm between two local searches (*local.n2*, line 11 in Algorithm 2).
- Balance between intensification and diversification in the selection of initial points for the local searches (*balance*, line 15 in Algorithm 2).

All these settings have qualitatively the same influence on the algorithm’s behavior: large setting values lead to conservative executions, while small values lead to aggressive executions.

**Fig. 5:**
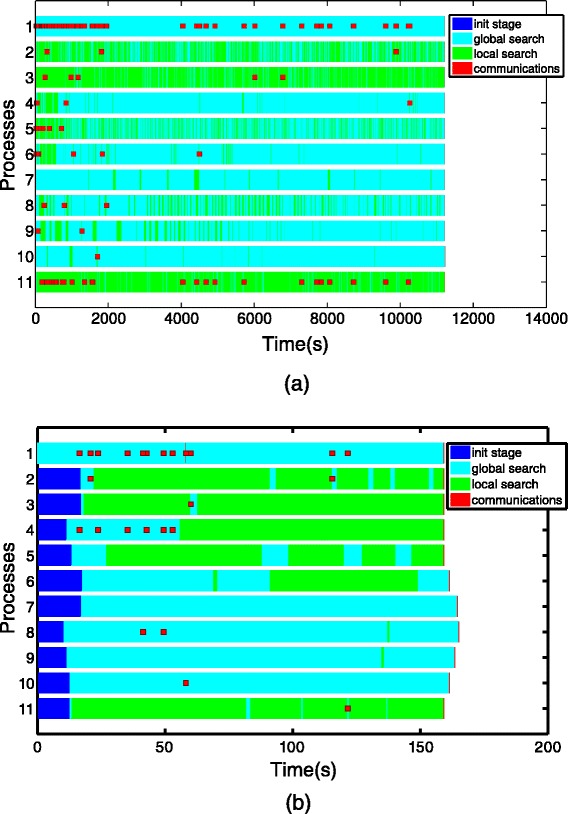
Gantt diagrams representing the tasks and cooperation between islands against execution time. Process 1 is the master, and processes 2-11 are slaves (islands). *Red dots* represent asynchronous communications between master and slaves. Light blue marks represent global search steps, while green marks represent local search steps. These figures correspond to two different examples, intended to illustrate the design decisions, described in the text, taken in the self-adaptive procedure to identify successful and failure islands. **a** Gantt diagram 1 and **b** Gantt diagram 2

Designing a self-adaptive procedure that identifies those islands that are becoming failures and those that are successful is not an easy task. To decide the most promising islands, the master process serves as a scoreboard where the islands are ranked according to their potential. In the rating of the islands, two facts have to be considered: (1) the number of total communications received in the master from each islands, to identify promising islands among those that intensively cooperates with new *good* solutions; and (2) for each island, the moment when its last solution has been received, to prioritize those islands that have more recently cooperated. A good balance between these two factors will produce a more accurate scoreboard. To better illustrate this problem Fig. 5(a) shows, as an example, a Gantt diagram where the communications are colored in red. Process 1 is the master process, while process 2-11 are the slaves (islands). At a time of *t*=2000, process 5 has a large number of communications performed, however, all these communications were performed a considerable time ago. On the other hand, process 2 has just communicated a new solution, but presents a smaller number of total communications performed. To accurately update the scoreboard, the rate of each island is calculated in the master as the product of the number of communications performed and the time elapsed from the beginning of the execution until the last reception from that island. In the example above, process 6 achieves a higher rate because it presents a better balance between the number of communications and the time elapsed since the last reception.

Identifying the *worst* islands is also cumbersome. Those processes at the bottom of the scoreboard are there because they do not communicate sufficient solutions or because a considerable amount of time has passed since their last communication. However, they can be either non-cooperating (less promising) islands or more aggressive ones. An aggressive thread often calls the local solver, performing longer iterations, and thus being unable to communicate results as often as conservative islands can do so. To better illustrate this problem, Fig. 5(b) shows a new Gantt diagram. At a time of *t*=60, process 4 will be at the top of the scoreboard because it often obtains promising solutions. This is a conservative island. Process 3 will be at the bottom of the scoreboard because at that time it has not yet communicated a significant result. The reason is that process 3 is an aggressive slave that is still performing its first local search. To accurately identify the non-cooperating islands, the master process would need additional information from islands that would imply extra messages in each iteration of the eSS. The solution implemented is that each island decides by itself whether it is evolving in a promising mode or not. If an island detects that it is receiving cooperative solutions from the master but it cannot improve its results, it will send the master a reconfiguration request. The master, will then communicate to this island the settings of the island on the top of the scoreboard.

#### Comprehensive overview of the saCeSS algorithm

The pseudocode for the master process is shown in Algorithm 4, while the basic pseudocode for each slave is shown in Algorithm 5.





At the beginning of the algorithm, a local variable present in the master and in each slave is declared to keep track of the best solution shared in the cooperation step. The master process sets the initial threshold, initiates the scoreboard to keep track of the cooperation rate of each slave, and begins to listen to the requests and cooperations arriving from the slaves. Each time the master receives from a slave a solution that significantly improves the current best known solution (*BestKnownSol*), it increments the score of this slave on the board.

Each slave creates its own population matrix of *ndiverse* solutions. Then an initial *RefSet* is generated for each process with *dimRefSet* solutions with the best elements and random elements. Again, different *dimRefSet* are possible for different processes. The rest of the operations are performed within each *RefSet* in each process, in the same way as in the serial eSS implementation. Every external iteration of the algorithm, a cooperation phase is performed to exchange information with the rest of the processes in the parallel application. Whenever a process reaches the cooperation phase, it checks if any message with a new best solution from the master has arrived at its reception memory buffer. If a new solution has arrived, the process checks whether this new solution improves the *BestKnownSol* or not. If the new solution improves the current one, the new solution promotes to be the *BestKnownSol*. The loop to check the reception of new solutions must be repeated until there are no more shared solutions to attend. This is because the execution time of one external iteration may be very different from one process to another, due to the diversification strategy explained before. Thus, while a process has completed only one external iteration, their neighbors may have completed more and several messages from the master may be waiting in the reception buffer. Then, the *BestKnownSol* has to replace the cooperation entry in the process *RefSet*.

After the reception step, the slave process checks whether its best solution improves in, at least, an *ε* the *BestKnownSol*. If this is the case, it updates *BestKnownSol* with its best solution and sends it to the master. Note that the *ε* used in the slaves is not the same as the *ε* used in the master process. The slaves use a rather small *ε* so that many *good* solutions will be sent to the master. The master, in turn, is in charge of selecting those incoming solutions that are qualified to be spread, thus, its *ε* begins with quite a large value that decreases when the number of refused solutions increases and no incoming solution overcomes the current *ε*.

Finally, before the end of the iteration, the adaptive phase is performed. Each slave decides if it is progressing in the search by checking if:

$$N_{eval} \textgreater N_{par}\times 5000 $$

where *N*_*eval*_ is the number of evaluations performed by this process since its last cooperation with the master and *N*_*par*_ is the number of parameters of the problem. Thus, the reconfiguration condition depends on the problem at hand. Besides, if the number of received solutions is greater than the number of solutions sent, that is, if other processes are cooperating much more than itself, the reconfiguration condition is also met. Summarizing, if a process detects that it is not improving while it is receiving solutions from the master, it sends a request for reconfiguration to the master process. The master listens to these requests and sends to those slaves the settings of the most promising ones, i.e., those that are on the top of the scoreboard.

Finally, the saCeSS algorithm repeats the external loop until the stopping criterion is met. The current version can consider three different stopping criteria (or any combination among them): maximum number of evaluations, maximum execution time and a *value-to-reach (VTR)*. While the *VTR* is usually known in benchmark problems (such as those used below), for a new problem, the *VTR* will be, in general, unknown. Thus, in such more realistic cases, the recommended practice in metaheuristics is to perform some trial runs and then analyze the convergence curves in order to find sensible values for the maximum number of evaluations and/or execution time.

**Fig. 6:**
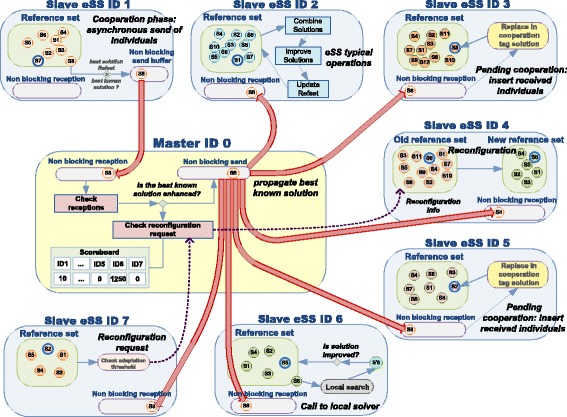
saCeSS: self-adaptive Cooperative enhanced Scatter Search. An example representation of the different mechanisms of communication and adaptation proposed in saCeSS

For illustrative purposes, Fig. 6 graphically shows the self-adaptive Cooperative enhanced Scatter Search (saCeSS) proposed. Note that different processes (islands) are executing a different eSS. Since they run asynchronously, they might be in different stages at every time moment. Thus, cooperation between these different searches should also be performed in an asynchronous fashion, avoiding stalls if any of the islands is involved in a time consuming phase, such as the execution of the local solver (see process ID 6 in the figure), while other islands (processes ID 1, ID 3 and ID 5 in the figure) are in the cooperation phase. When an island cannot attend to a cooperation reception, the message will be stored in the process as a pending cooperation, avoiding the blocking of the sender process (see processes ID 3 or ID 5 in the figure, attending pending cooperations). The master process (ID 0 in the figure) is in charge of the cooperation between parallel searches and their long-term behavior. It maintains a scoreboard to guide the adaptive procedure that tunes the settings of the eSS in the different islands. When an island detects that it is not progressing in its search (see process ID 7 in the figure), it sends the master a reconfiguration request. The master communicates, to those islands that request a reconfiguration, the settings of the most promising searches according to its scoreboard (see process ID 4 in the figure).

## Results and discussion

The proposed self-adaptive cooperative algorithm (saCeSS) has been applied to a set of benchmarks from the BioPreDyn-bench suite [66], with the goal of assessing its efficiency in challenging parameter estimation problems in computational system biology:

- *Problem B1*: genome-wide kinetic model of *S. cerevisiae*. It contains 276 dynamic states, 44 observed states and 1759 parameters.
- *Problem B2*: dynamic model of the central carbon metabolism of *E. coli*. It consists of 18 dynamic states, 9 observed states and 116 estimable parameters.
- *Problem B3*: dynamic model of enzymatic and transcriptional regulation of the central carbon metabolism of *E. coli*. It contains 47 dynamic states (fully observed) and 178 parameters to be estimated.
- *Problem B4*: kinetic metabolic model of Chinese Hamster Ovary (CHO) cells, with 34 dynamic states, 13 observed states and 117 parameters.
- *Problem B5*: signal transduction logic model, with 26 dynamic states, 6 observed states and 86 parameters.
- *Problem B6*: dynamic model describing the gap gene regulatory network of the vinegar fly, *Drosophila melanogaster*. It consists of three processes formalized with 108-212 ODEs, and resulting in a model with 37 unknown parameters.

Different experiments have been conducted using the saCeSS method, and its performance has been compared with two different parallel versions of the eSS, namely: an embarrassingly-parallel non-cooperative enhanced Scatter Search (*np*-eSS), and a previous cooperative synchronous version (CeSS) [51]. The *np*-eSS algorithm consists of *np* independent eSS runs (being *np* the number of available processors) performed in parallel without cooperation between them and reporting the best execution time of the *np* runs. Diversity is introduced in these *np* eSS runs, alike in the cooperative methods, that is, each one executes a separate eSS using different strategies.

It should be noted that the eSS [57] and CeSS [51] methods have been originally implemented in Matlab. Both algorithms have been now coded in F90 to perform an honest comparison with the proposed saCeSS method. For the same reason, the CeSS method has been re-written using the MPI library for the communications between master and slaves, since its original reported implementation used *jPar* [67].

The experiments reported here have been performed in a multicore cluster with 16 nodes powered by two octa-core Intel Xeon E5-2660 CPUs with 64 GB of RAM. The cluster nodes were connected through an InfiniBand FDR network. For problem *B3* this cluster could not be used because the execution of *B3* exceeds the maximum allowed job length. Thus, another multicore heterogeneous cluster was used for this problem that consists of 4 nodes powered by two quadcore Intel Xeon E5420 CPUs with 16 GB of RAM and 8 nodes powered by two quadcore Intel Xeon E5520 CPUs with 24 GB of RAM, connected through a Gigabit Ethernet network.

The computational results shown in this paper were analyzed from a horizontal view [68], that is, assessing the performance by measuring the time needed to reach a given target value. To evaluate the efficiency of the proposal, experiments with a stopping criteria based on a *value-to-reach (VTR)* were performed. The *VTR* used was the optimal fitness value reported in [66]. Also, since comparing different metaheuristic is not an easy task, due to the substantial dispersion of computational results due to the stochastic nature of these methods, each experiment reported in this section was performed 20 times and a statistical study was carried out.

### Performance evaluation of the coarse-grained parallelization and the self-adaptive mechanism

The cooperation between processes in the coarse-grained parallelization can change the systemic properties of the eSS algorithm and therefore its macroscopic behavior. The same happens with the self-adaptive mechanism proposed. Thus, the first set of experiments shown in this section disables the fine-grained parallelization, since it does not alter the convergence properties of the algorithm, to evaluate solely the impact of the coarse-grained parallelization, as well as the self-adaptive mechanism.

Table 1 displays, for each benchmark and each method, the number of external iterations performed (line 7 in Algorithm 1), the average number of evaluations needed to achieve the *VTR*, the mean and standard deviation execution time of all the runs in the experiment and the speedup achieved versus the non-cooperative case (*10*-eSS). In these experiments, the computational load is not shared among processors. Therefore, the total speedup achieved over the *np*-eSS method depends on the impact that the cooperation among processes produces on achieving a good result, performing less evaluations and, hence, providing a better performance. We compare the CeSS method, that exchanges the information based on a time elapsed, and where the information to be exchanged is the complete *RefSet*, and the proposed saCeSS method, that exchanges the information driven by the quality of the solutions through a star topology using asynchronous communications and, thus, avoiding communication delays. Additionally, to better evaluate the efficiency of the self-adaptive procedure proposed, Table 1 also displays results for two different experiments with the saCeSS method: experiments where the self-adaptive procedure was disabled, labeled as saCeSS(non-adaptive) in the table, that is, the settings of the eSS in the different islands were not dynamically tuned during the execution; and, experiments where this procedure was enabled, allowing for the adaptive reconfiguration of the islands, labeled as saCeSS in the table.

**Table 1:** Performance of the coarse-grained parallelization and the self-adaptive scheme proposed

P	Method	Mean iter ±std	Mean evals ±std	Mean time ±std(s)	Speedup
	*10*-eSS	80 ±30	199214 ±44836	5378±1070	-
	CeSS (*τ*=700*s*)	109 ±49	188131 ±82834	6487±3226	0.83
*B1*	CeSS (*τ*=1400*s*)	122 ±41	175331 ±98255	5018±1477	1.07
	saCeSS(non-adaptive)	92 ±35	143145 ±61828	3759±976	1.43
	saCeSS	62 ±21	92122 ±35058	2753±955	1.95
	*10*-eSS	450 ±167	1504503 ±541257	1914±714	-
	CeSS (*τ*=400*s*)	452 ±278	1637125 ±1016688	2459±2705	0.78
*B2*	CeSS (*τ*=800*s*)	508 ±205	1802917 ±690613	1911±1103	1.00
	saCeSS(non-adaptive)	440 ±192	1528793 ±647677	1918±833	1.00
	saCeSS	846 ±982	1247699 ±1222378	1694±1677	1.13
	*10*-eSS	10062 ±2528	66915128 ±15623835	511166±135988	-
	CeSS(*τ*=50000*s*)	7288 ±5551	52592578 ±35513874	332721±245829	1.53
*B3*	saCeSS(non-adaptive)	4323 ±3251	32604331 ±23357322	251305±209082	2.03
	saCeSS	4113 ±3130	27647470 ±21488783	229888±238970	2.22
	*10*-eSS	99 ±121	2230089 ±2068300	750±692	-
	CeSS (*τ*=100*s*)	140 ±386	1665954 ±2921838	817±1909	0.92
*B4*	CeSS (*τ*=200*s*)	119 ±87	1649723 ±1024833	518±428	1.45
	saCeSS(non-adaptive)	39 ±30	1163458 ±927751	402±303	1.86
	saCeSS	35 ±24	1017956 ±728328	343±240	2.18
	10-eSS	16 ±4	69448 ±14570	901±197	-
	CeSS (*τ*=200*s*)	11 ±4	108481 ±36190	1481±634	0.61
*B5*	CeSS (*τ*=400*s*)	14 ±3	94963 ±20172	996±264	0.90
	saCeSS(non-adaptive)	10 ±2	49622 ±9530	637±131	1.42
	saCeSS	10 ±3	51076 ±12696	658±174	1.37
	*10*-eSS	4659 ±3742	9783720 ±8755231	8217±7536	-
	CeSS (*τ*=1000*s*)	5919 ±5079	10475485 ±8978383	8109±7441	1.01
*B6*	CeSS (*τ*=2000*s*)	6108 ±6850	10778260 ±12157617	7878±9400	1.04
	saCeSS(non-adaptive)	2501 ±1517	4394243 ±2689489	3638±2302	2.26
	saCeSS	1500 ±1265	2594741 ±2214235	2177±1933	3.77

Execution time and speedup results using 10 processors. Stopping criteria: *VTR*_*B*1_=1.3753×10^4^, *VTR*_*B*2_=2.50×10^2^, *VTR*_*B*3_=3.7×10^−1^, *VTR*_*B*4_=55, *VTR*_*B*5_=4.2×10^3^, *VTR*_*B*6_=1.0833×10^5^

The results of the saCeSS (non-adaptive) show that the cooperation mechanisms and the asynchronous communication protocol proposed in the saCeSS method improve the results obtained by *10*-eSS and CeSS, both in terms of number of evaluations and execution time. As regards the CeSS method, the results can be explained both because the synchronization slows down the processes, since it implies more processes’ stalls while waiting for data, and because of the effectiveness of the information exchanged. The results of the saCeSS show that the self-adaptive approach improves the previous results even more. The main goal of this approach is to reduce the impact that the initial choice of the configurable settings may have in the evolution of the method. For instance, one issue of the CeSS algorithm is the selection of the migration time (*τ*), that is, the time between information sharing. On the one hand, this time has to be long enough to allow each of the threads to exploit the eSS capabilities. On the other hand, if the time is too long, cooperation will happen only sporadically, reducing its efficiency. On the contrary, in the saCeSS method proposed, the initial selection of the threshold to spread a cooperative solution, as well as the selection of the other configurable settings of the eSS method, will change adaptively during the execution progress.

**Fig. 7:**
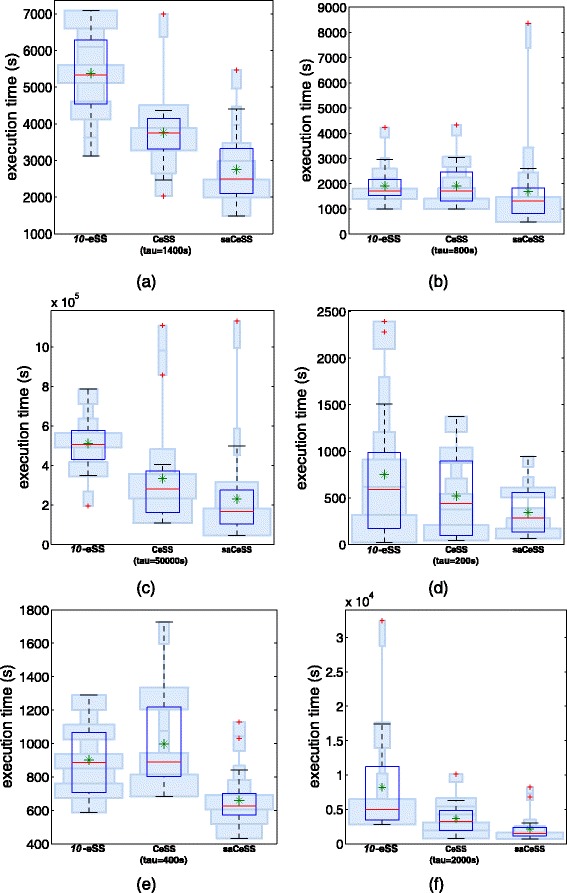
Hybrid violin/Box plots of the execution times using 10 processes. Results for experiments reported in Table 1.The *green* asterisks correspond to the mean and *light blue* boxes illustrate the distribution of the results. Each box with a strong *blue* contour represents a typical boxplot: the central red line is the median, the edges of the box are the 25th and 75th percentiles, and outliers are plotted with red crosses. (**a**) B1, (**b**) B2, (**c**) B3, (**d**) B4, (**e**) B5 and (**f**) B6

When dealing with stochastic optimization solvers, it is important to evaluate the dispersion of the computational results. Figure 7 illustrates how the proposed saCeSS reduces the variability of execution time in the non-cooperative *10*-eSS method. This is an important feature of the saCeSS, because it reduces the average execution time for each benchmark. For instance, even in the B2 problem, where the mean execution time is similar in the non-cooperative and the cooperative executions, the dispersion of the results is reduced in the cooperative case.

Finally, to prove the significance of the results, a non-parametric statistical analysis has been applied to the final runtimes of the experiments over each test problem. Recent studies show that the most appropriate methods to compare the performance of different metaheuristics are the nonparametric procedures [69]. In this work we have applied the Kruskal-Wallis test [70] followed by Dunn’s test [71], that reports the results among multiple pairwise comparisons after the Kruskall-Wallis test. The objective is to explain if the observed differences among final runtimes for each problem are due to the optimization method used (i.e., *np*-eSS, CeSS and saCeSS) or to pure randomness. Table 2 shows, for each problem, the *p*-values of Dunn’s test for every pair comparison after the Kruskall-Wallis test application. Table 3 shows, for each problem, the classification of methods in groups at 95% confidence level according to the p-values shown above. As shown in Tables 2 and 3, the statistical results reveal that saCeSS shows better performance than the other two methods in the problems considered. It is clear for problems B1, B3, B5 and B6. For problems B2 and especially B4, the differences are not as significant as in the other problems. For B2, the reason is the outlier in one of the runs with saCeSS in problem B2, which damages the results since it is the worst value among all runs and methods for this problem. For B4, the reason is the high dispersion of the results, together with the fact that many experiments achieved the convergence in the first iterations of the algorithm (note the swelling at the bottom of the violin/box plots in Fig. 7), before the cooperation turned into effective, or the self-adaptive procedure became operational, thus, reducing the difference among the three algorithms.

**Table 2:** *p*-values of the pairwise comparisons provided by the Dunn’s test

	B1	B2	B3	B4	B5	B6
saCeSS vs CeSS	0.0000	0.0630	0.0380	0.1924	0.0000	0.0000
saCeSS vs *10*-eSS	0.0000	0.0115	0.0000	0.0324	0.0001	0.0000
CeSS vs *10*-eSS	0.1850	0.2289	0.0006	0.1641	0.2128	0.3964

**Table 3:** Group classification of the optimization methods at 95% confidence level

	B1		B2 ^a^		B3			B4		B5		B6	
saCeSS	A		A		A			A		A		A	
CeSS		B	A	B		B		A	B		B		B
*10*-eSS		B		B			C		B		B		B

^a^For problem B2, the classification at 90% confidence interval is:

saCeSS(A), CeSS(B), *np*-eSS(B)

**Fig. 8:**
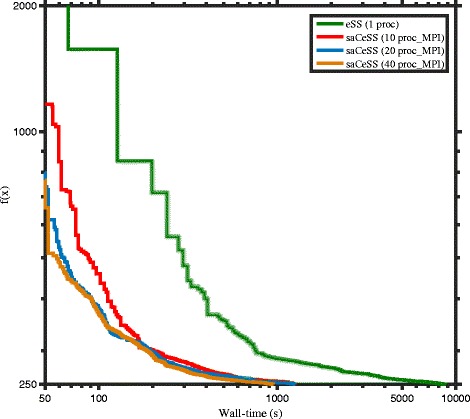
Scalability of self-adaptive Cooperative enhanced Scatter Search (saCeSS) using 1, 10, 20 and 40 processors in problem B2. Note that the saCeSS method using 1 MPI process is equivalent to the eSS method using 1 processor. For each method, the convergence curves that are closer to the median of the results distribution are shown in the figure. Detailed distributions for all the benchmarks can be found in Additional file 1

The CeSS method presents an important issue related to its scalability, since the cooperation among islands is carried through a synchronous communication step. To assess the scalability of the coarse-grained parallelization proposed in saCeSS, experiments were carried out using 1 (that is a single eSS), 10, 20 and 40 processors. The convergence curves for B2 benchmark (see Additional file 1 for the others problems) are shown in Fig. 8. This curves represent the logarithm of the objective function value against the execution time for those experiments that fall in the median values of the results distribution. Similar results are obtained for the rest of the benchmarks, and they are reported in the supplementary information. As it can be observed, when the number of processors grows, the saCeSS method keeps on improving the convergence results. This is due to the proposed asynchronous communication protocol. However, as the number of cooperative processes increases, the improvement in the algorithm performance is restrained. This is readily justified, because the self-adaptive mechanism will drive the different processes from different initial parameters to those settings that obtain successful results, which, in the long term, means that having a larger number of processes does not aim to a larger diversity and better results. Thus, the hybrid MPI+OpenMP proposal aims to improve the performance results when the number of processors increases, by combining the MPI stimulation on diversification in the search with the OpenMP intensification.

### Performance evaluation of the hybrid MPI+OpenMP proposal

The goal of the fine-grained parallelization proposed in this paper is to perform the evaluation of the obtained solutions in parallel threads, thus, accelerating the execution without altering the properties of the algorithm. As already commented in previous sections, the scalability of the fine-grained parallelization is limited in the eSS algorithm, due to the small *dimRefSet*. Also, the workload is uneven, since different evaluations lead some threads to be busy for longer times. Thus, the dynamic schedule used allows the threads with small workloads to go after other chunks of work, and hopefully, balance the work between threads. But it introduces a large overhead at runtime, as work has to be taken off of a queue.

Table 4 shows the performance and scalability of the hybrid MPI+OpenMP implementation proposed in this work. Benchmarks B3 and B6 were excluded of these evaluations: B3 due to our lack of available resources to run such long executions, and B6 since its currently available implementation could not be parallelized using the OpenMP library. Note that the *np*-eSS algorithm was executed also using 10, 20 and 40 processes (non-cooperative), to perform a fair comparison with saCeSS using 10, 20 and 40 processors respectively. The speedup was calculated accordingly. In most of the cases, the hybrid configurations obtain better performance than other configurations that only use MPI processes. In general, results show that, for the same number of processors, those hybrid configurations that achieve a good balance between intensification and diversification perform more effectively. For instance, the configuration of 5 MPI processes with 4 OpenMP threads each, achieves, in general, better speedup than the configuration with 10 MPI processes with 2 OpenMP threads each, while both use 20 processors. The exception is benchmark B4, whose performance is heavily affected by the number of different processes cooperating. That is, benchmark B4 greatly benefits from the diversity introduced with the scalability in the number of MPI processes versus the intensification of the OpenMP search. Thus, for benchmark B4, configurations using all the available processors to run MPI cooperative processes perform better.

**Table 4:** Performance of the hybrid MPI+OpenMP saCeSS and scalability analysis when the number of processors grows

P	Method	# proc. config.	Mean iter ±std	Mean evals ±std	Mean time ±std(s)	Speedup
		# (MPI x OpenMP)				
*B1*	*10*-eSS	10	80 ±30	199214 ±44836	5378±1070	-
	saCeSS	10 (10 x 1)	62 ±21	92122 ±35058	2753±955	1.95
	saCeSS	10 (5 x 2)	76 ±40	88186 ±46200	2762±1273	1.95
	*20*-eSS	20	106 ±34	304972 ±88870	4524±942	-
	saCeSS	20 (20 x 1)	79 ±38	218871 ±107769	3125±1349	1.45
	saCeSS	20 (10 x 2)	86 ±43	147268 ±75752	2299±1199	1.97
	saCeSS	20 (5 x 4)	54 ±19	68907 ±27132	1288±380	3.51
	*40*-eSS	40	79 ±21	455091 ±121875	3662±718	-
	saCeSS	40 (40 x 1)	51 ±19	274628 ±115644	2070±650	1.77
	saCeSS	40 (20 x 2)	64 ±27	205766 ±93753	1743±759	2.10
	saCeSS	40 (10 x 4)	58 ±30	111804 ±61212	1130±535	3.24
	saCeSS	40 (5 x 8)	57 ±29	82414 ±38357	1078±494	3.39
*B2*	*10*-eSS	10	450 ±167	1504503 ±541257	1914±714	-
	saCeSS	10 (10 x 1)	846 ±982	1247699 ±1222378	1694±1677	1.13
	saCeSS	10 (5 x 2)	630 ±275	916166 ±357665	1298±700	1.47
	*20*-eSS	20	355 ±130	2306298 ±851619	1465±546	-
	saCeSS	20 (20 x 1)	649 ±287	1901601 ±747379	1345±619	1.09
	saCeSS	20 (10 x 2)	904 ±646	1388211 ±854461	962±616	1.52
	saCeSS	20 (5 x 4)	609 ±244	925076 ±353594	734±320	2.00
	*40*-eSS	40	297 ±108	3878818 ±1478105	1223±447	-
	saCeSS	40 (40 x 1)	571 ±542	3286363 ±2593258	1326±1764	0.92
	saCeSS	40 (20 x 2)	766 ±863	2185567 ±2228163	951±1468	1.29
	saCeSS	40 (10 x 4)	756 ±243	1309744 ±446419	506±180	2.42
	saCeSS	40 (5 x 8)	522 ±290	801661 ±380553	353±162	3.46
*B4*	*10*-eSS	10	99 ±121	2230089 ±2068300	750±692	-
	saCeSS	10 (10 x 1)	35 ±24	1017956 ±728328	343±240	2.18
	saCeSS	10 (5 x 2)	177 ±269	1854102 ±2331070	916±1210	0.82
	*20*-eSS	20	18 ±22	1206948 ±1177645	176±203	-
	saCeSS	20 (20 x 1)	11 ±8	819845 ±548341	111±93	1.58
	saCeSS	20 (10 x 2)	30 ±23	898459 ±657350	215±169	0.82
	saCeSS	20 (5 x 4)	294 ±692	2090044 ±3980316	736±1453	0.24
	*40*-eSS	40	12 ±13	1811454 ±1608998	121±141	-
	saCeSS	40 (40 x 1)	9 ±5	1254545 ±603925	78±51	1.55
	saCeSS	40 (20 x 2)	26 ±26	1523817 ±1267964	159±160	0.76
	saCeSS	40 (10 x 4)	37 ±50	878008 ±1178953	168±238	0.72
	saCeSS	40 (5 x 8)	159 ±692	1666511 ±3980316	490±446	0.25

**Table 4:** Performance of the hybrid MPI+OpenMP saCeSS and scalability analysis when the number of processors grows (*Continued*)

*B5*	*10*-eSS	10	16 ±4	69448 ±14570	901±197	-
	saCeSS	10 (10 x 1)	10 ±3	51076 ±12696	658±174	1.37
	saCeSS	10 (5 x 2)	11 ±3	36090 ±8761	520±136	1.73
	*20*-eSS	20	11 ±2	106051 ±17602	685±127	-
	saCeSS	20 (20 x 1)	10 ±2	99995 ±21070	654±151	1.05
	saCeSS	20 (10 x 2)	9 ±2	52419 ±12040	368±92	1.86
	saCeSS	20 (5 x 4)	10 ±3	38427 ±10523	331±97	2.07
	*40*-eSS	40	10 ±2	192192 ±29082	618±102	-
	saCeSS	40 (40 x 1)	8 ±2	161687 ±40392	514±138	1.20
	saCeSS	40 (20 x 2)	9 ±2	100315 ±17758	350±68	1.76
	saCeSS	40 (10 x 4)	11 ±2	64594 ±13173	280±61	2.21
	saCeSS	40 (5 x 8)	11 ±3	45854 ±11607	248±67	2.48

Execution time and speedup results. Stopping criteria: *VTR*_*B*1_=1.3753×10^4^, *VTR*_*B*2_=2.50×10^2^, *VTR*_*B*3_=3.7×10^−1^, *VTR*_*B*4_=55, *VTR*_*B*5_=4.2×10^3^, *VTR*_*B*6_=1.0833×10^5^

**Fig. 9:**
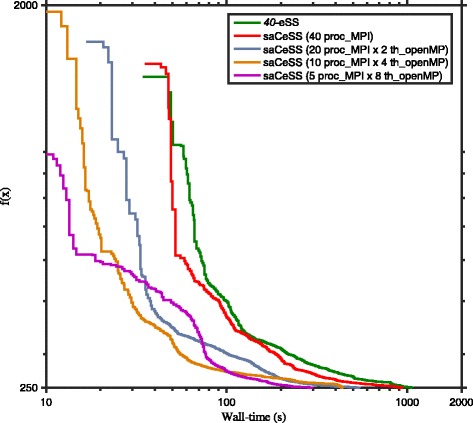
Comparative between non-cooperative enhanced Scatter Search (eSS) and self-adaptive Cooperative enhanced Scatter Search (saCeSS) using 40 processors in problem B2. The performance of 40 individual, non-cooperative eSS is compared with different hybrid configurations of the saCeSS method using 40 processors. For each method, the convergence curves that are closer to the median of the results distribution are shown in the figure. Detailed distributions for all the benchmarks can be found in Additional file 1

Figure 9 shows, for B2 benchmark (see Additional file 1 for the others problems), the convergence curves for those experiments that fall in the median values of the results distribution. Similar results are obtained for the rest of the benchmarks, and they are reported in the supplementary information. The performance of 40 individual, non cooperative processes, is compared to the performance of saCeSS method with different hybrid configurations using 40 processors in all of them. As it has already been pointed out, when the number of available processors increases, hybrid MPI+OpenMP configurations will improve the performance versus a solely coarse-grained parallelization (see Fig. 9). This is an important result because it allows us to predict a good performance of this method on HPC systems, built as clusters of multicore nodes, where MPI processes would be located in different nodes, improving diversification, while, within each node, the OpenMP threads would intensify the search.

**Fig. 10:**
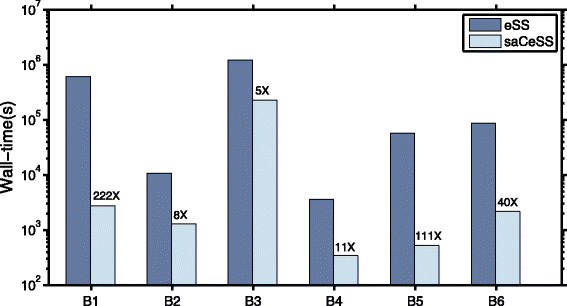
Performance acceleration of saCeSS versus eSS (as reported in [66]). Bars show wall-clock times for each solver and benchmark problem, with the numbers over saCeSS bars indicating the overall acceleration obtained. Computations with saCeSS were carried out with 10 processors while computations with eSS were carried out with 1 processor

Finally, Fig. 10 compares the results obtained by the proposed saCeSS method and the results reported in the BioPreDyn-bench suite [66]. Note that, although these figures correspond to a partially different language implementation of eSS (Matlab and C, as reported in [66]), the cost functions and the associated dynamics were computed using the same C code in both cases. It can be observed that the parallel cooperative scheme significantly reduces the execution time in the very complex problems as B1 (1 week vs 1 hour) or B3 problem (10 days vs less than 46 hours). These figures can be especially illustrative for those interested in the potential of HPC in general, and the saCeSS method in particular, in the solution of parameter estimation problems.

### Comparison with other parallel metaheuristics

In order to properly evaluate the novel parallel method presented here, we now compare it with a parallel version of another state of the art metaheuristic, Differential Evolution (DE [72]). Previous works in the literature have already pointed out that, for sequential implementations, eSS performs better than DE in those problems where local searches are instrumental in refining the solution [51]. Thus, to ensure a more fair comparison here, we chose a parallel DE implementation (asynPDE [43]) that performs a global search through an asynchronous parallel implementation based on a cooperative island-model, and that also improves the local search phase by means of several heuristics also used in the eSS (i.e. an efficient local solver, a tabu list and a logarithmic space search).

**Fig. 11:**
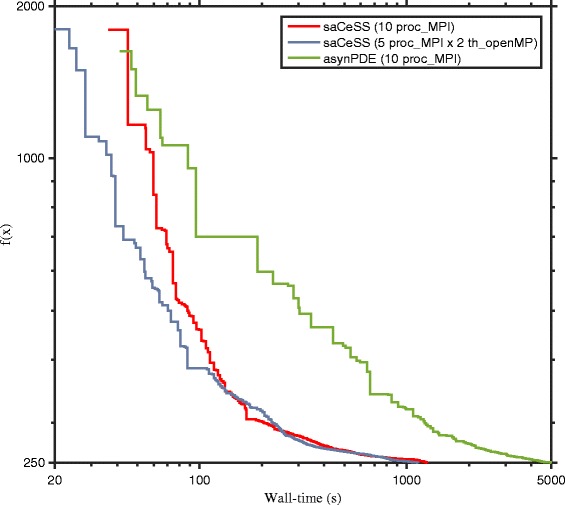
Comparison of the convergence curves of asynchronous parallel Differential Evolution (asynPDE) and self-adaptive Cooperative enhanced Scatter Search (saCeSS) using 10 processors in problem B2. For each method, the convergence curves that are closer to the median of the results distribution are shown in the figure. Detailed distributions for all the benchmarks can be found in Additional file 1

**Fig. 12:**
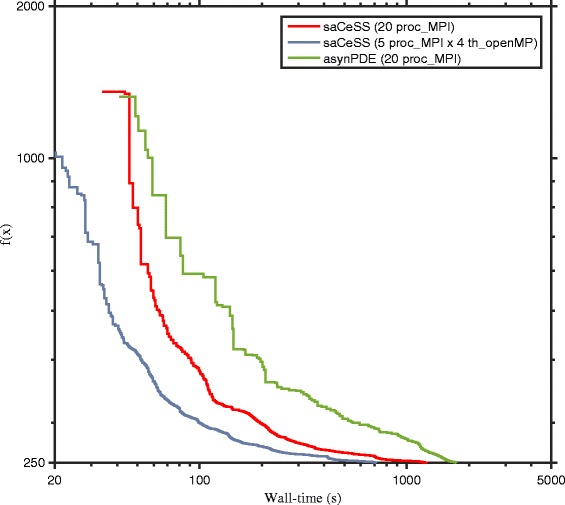
Comparison of the convergence curves of asynchronous parallel Differential Evolution (asynPDE) and self-adaptive Cooperative enhanced Scatter Search (saCeSS) using 20 processors in problem B2. For each method, the convergence curves that are closer to the median of the results distribution are shown in the figure. Detailed distributions for all the benchmarks can be found in Additional file 1

The convergence curves, considering problem B2 (see Additional file 1 for the others problems), of the asynPDE and the saCeSS algorithms for 10 and 20 processors are shown in Figs. 11 and 12 respectively. These figures represent the convergence curves for those experiments that fall in the median values of the results distribution. Although the best configuration for the saCeSS method is a hybrid MPI+OpenMP one, since the asynPDE method only performs a coarse-grained parallelization, for comparison purposes the convergence curves of saCeSS using only MPI processes are shown. As can be seen, in all cases asynPDE is significantly outperformed by saCeSS. Results for the other benchmarks can be found in the supplementary information.

## Conclusions

In this contribution we present a novel parallel method for global optimization in computational systems biology: a self-adaptive parallel cooperative enhanced scatter search algorithm (saCeSS). A hybrid MPI+OpenMP implementation is proposed combining a coarse-grained parallelization, focused on stimulating the diversification in the search and the cooperation between different processes, with a fine-grained parallelization, aimed at accelerating the computation by performing separate evaluations in parallel. This novel approach results in a much more effective way of cooperation between different processes running different configurations of the scatter search algorithm.

The new proposed approach has four main features: (i) a coarse-grained parallelization using a centralized master-slave approach, where the master is in charge of the control of the communications between slaves (islands) and serves as a scoreboard to dynamically tune the settings of the islands based on its individual progress; (ii) a cooperation between processes driven by the quality of the solution obtained during the execution progress, instead of by time elapsed; (iii) an asynchronous communication protocol that minimizes processes’ halts, thus improving the efficiency in a computational unbalanced scenario; and (iv) a fine-grained parallelization is included in each process to perform separate cost-function evaluations in parallel and, thus, accelerate the global search.

The proposed saCeSS method was evaluated considering a suite of very challenging benchmark problems from the domain of computational systems biology. The computational results show that saCeSS attains a very significant reduction in the convergence time through cooperation of the parallel islands (from days to minutes in several cases, using a small number of processors). A nonparametric statistical analysis shows that the saCeSS method significantly outperforms other parallel eSS approaches, such as an embarrassingly-parallel non-cooperative eSS algorithm, and a previous synchronous cooperative proposal.

These results indicate that parameter estimation in large-scale dynamic models of biological systems can be feasible even when using small and low-cost parallel machines, thus opening new perspectives towards the development and calibration of whole-cell models [73].

## Additional file

Additional file 1 Supplementary info document. This document is a complete report for all experiments performed for this work. (PDF 9 305 kb)

## Abbreviations

SS: Scatter search
eSS: enhanced scatter search
CeSS: Cooperative enhanced scatter search
saCeSS: Self-Adaptive cooperative enhanced scatter search
MPI: Message passing interface
openMP: Open multi-processing
NLP: NonLinear programming
DAE: Differential algebraic equations
HPC: High performance computing
F90: Fortran 90
DE: Differential evolution
asynPDE: asynchronous parallel differential evolution
VTR: Value to reach
