# Supplementary material for: Parameter estimation in large-scale systems biology models: a parallel and self-adaptive cooperative strategy
Source: BMC Bioinformatics. 2017 Jan 21;18:52. doi: 10.1186/s12859-016-1452-4 (PMC5251293; doi:10.1186/s12859-016-1452-4)
Supplement: Additional file 1 — Supplementary info document. This document is a complete report for all experiments performed for this work. (PDF 9 305 kb) [file 12859_2016_1452_MOESM1_ESM.pdf]

Parameter estimation in large-scale systems biology models: a parallel and self-adaptive cooperative strategy (supplementary information)

David R. Penas*[1], Patricia González†[2], José A. Egea‡[3], Ramón Doallo§[2] and Julio R. Banga¶[1]

[1]BioProcess Engineering Group, IIM-CSIC, Vigo (Spain)
[2]Computer Architecture Group, Universidade da Coruña (Spain)
[3]Department of Applied Mathematics and Statistics, Universidad Politécnica de Cartagena (Spain)

*david.penas@iim.csic.es
†patricia.gonzalez@udc.es
‡josea.egea@upct.es
§doallo@udc.es
¶julio@iim.csic.es

Contents

1 Source code availability **2**

2 Configuration settings for each experiment **3**

2.1 Local solvers 7

3 Measurement methodology and reported results **8**

4 Performance comparison among different parallel eSS schemes **11**

5 Scalability analyses of the proposed saCeSS method **52**

6 Performance analysis of the hybrid MPI+OpenMP saCeSS implementation **56**

7 Comparative between saCeSS and asynPDE **81**

1 Source code availability

There is a public distribution of a package called "saCeSS library", where the method proposed in this manuscript was implemented. The code is available at:

`https://bitbucket.org/DavidPenas/sacess-library`

This repository includes detailed documentation at:

[sacess-library]/doc/manual

Moreover, a set of scripts to reproduce the results were included in the repository in:

[sacess-library]/reproducibility_scripts

2 Configuration settings for each experiment

There are several tunable parameters in the eSS algorithm, and their values may have a significant impact on its performance. In the parallel eSS schemes evaluated in this paper (i.e., cooperative and non-cooperative) each parallel process adopts a different strategy to increase the diversification in the search. In other words, each parallel process performs a different eSS with a different degree of *aggressiveness*. An *aggressive* process performs frequent local searches, trying to refine the solution very quickly, and keeps a small reference set of solutions. It will perform well in problems with parameter spaces that have a smooth shape. On the other hand, *conservative* processes have a large reference set and perform local searches only sporadically. They spend more time combining parameter vectors and exploring the different regions of the parameter space. Thus, they are more appropriate for problems with rugged parameter spaces. Since the exact nature of the problem at hand is always unknown, it is recommended to choose, at the beginning of the scheme, a range of settings that yields conservative, aggressive, and intermediate processes.

There are several configurable settings that determine the strategy (conservative/aggressive) used by the sequential eSS algorithm:

- Number of elements in the reference set (*dimRefSet*).
- Minimum number of iterations of the eSS algorithm between two local searches (*local.n2*).
- Balance between intensification and diversification in the selection of initial points for the local searches (*balance*).
- Number of diverse solutions initially generated (*ndiverse*).

All these settings have qualitatively the same influence on the algorithm's behavior: large values lead to conservative executions, while small values lead to aggressive executions. It is advisable to use a broad spectrum of aggressiveness, such as the recommended default values [1] $0.5 \times npar$ (number of parameters) $< dimRefSet < 20 \times npar$; $0 < local.n2 < 100$; $0 < balance < 0.5$; and $5 \times npar < ndiverse < 20 \times npar$.

In order to evaluate the proposed self-adaptive cooperative algorithm (saCeSS), the benchmarks from the BioPreDyn-bench suite [2] have been tested. These are challenging parameter estimation problems from the domain of computational system biology. The following were the settings used for the experiments reported in the paper:

- *Problem B1*: genome-wide kinetic model of *S. cerevisiae*. It contains 276 dynamic states, 44 observed states and 1759 parameters. Common settings: ndiverse population=100; local solver $=dhc$; VTR=13753.

Specific settings per process (B1)

slave	dimRefset	n2	balance
1	7	1	0.0
2	11	4	0.0
3	14	10	0.25
4	20	20	0.5
5	24	100	0.25
6	22	50	0.25
7	17	15	0.25
8	14	7	0.25
9	9	2	0.0
10	5	1	0.0

- *Problem B2*: dynamic model of the central carbon metabolism of *E. coli*. It consists of 18 dynamic states, 9 observed states and 116 estimable parameters. Common settings: ndiverse population = 1160; local solver = *none*; VTR = 250.

Specific settings per process (B2)

slave	dimRefset	n2	balance
1	12	1	0.0
2	20	4	0.0
3	25	10	0.25
4	35	20	0.5
5	43	100	0.25
6	38	50	0.25
7	30	15	0.25
8	25	7	0.25
9	16	2	0.0
10	9	1	0.0

- *Problem B3*: dynamic model of enzymatic and transcriptional regulation of the central carbon metabolism of *E. coli*. It contains 47 dynamic states (fully observed) and 178 parameters to be estimated. Common settings: ndiverse population = 1780; local solver = *dhc*; VTR = 0.37029.

Specific settings per process (B3)

slave	dimRefset	n2	balance
1	14	1	0.0
2	24	4	0.0
3	31	10	0.25
4	43	20	0.5
5	53	100	0.25
6	47	50	0.25
7	38	15	0.25
8	31	7	0.25
9	20	2	0.0
10	10	1	0.0

- *Problem B4*: kinetic metabolic model of Chinese Hamster Ovary (CHO) cells, with 34 dynamic states, 13 observed states and 117 parameters. Common settings: ndiverse population = 1170; local solver = *nl2sol*; VTR = 55.

Specific settings per process (B4)

slave	dimRefset	n2	balance
1	12	1	0.0
2	20	4	0.0
3	25	10	0.25
4	35	20	0.5
5	43	100	0.25
6	38	50	0.25
7	31	15	0.25
8	25	7	0.25
9	16	2	0.0
10	9	1	0.0

- *Problem B5*: signal transduction logic model, with 26 dynamic states, 6 observed states and 86 parameters. Common settings: ndiverse population = 860; local solver = *dhc*; VTR = 4200.

Specific settings per process (B5)

slave	dimRefset	n2	balance
1	10	1	0.0
2	17	4	0.0
3	22	10	0.25
4	30	20	0.5
5	37	100	0.25
6	33	50	0.25
7	26	15	0.25
8	22	7	0.25
9	14	2	0.0
10	8	1	0.0

- *Problem B6*: dynamic model describing the gap gene regulatory network of the vinegar fly, *Drosophila melanogaster*. It consists of three processes formalized with 108-212 ODEs, and resulting in a model having 37 unknown parameters. Common settings: ndiverse population = 370; local solver = *dhc*; VTR = 108330.

Specific settings per process (B6)

**slave**	**dimRefset**	**n2**	**balance**
1	7	1	0.0
2	12	4	0.0
3	15	10	0.25
4	20	20	0.5
5	25	100	0.25
6	22	50	0.25
7	18	15	0.25
8	15	7	0.25
9	10	2	0.0
10	5	1	0.0

2.1 Local solvers

Local solvers play a major role in the scatter search metaheuristic, accelerating convergence. Two different local solvers were used in results reported in our study:

- *nl2sol*, which stands for "Nonlinear Least-Squares Algorithm"[3]. This solver uses the Jacobian of the residual vector to approximate and iteratively improve the input solution. The version used is the nl2sol solver from the PORT library. IMPORTANT: in order to use this local solver, the user needs to provide the vector of residuals, in addition to the scalar objective (cost) function.

- *DHC*, which stands for "Dynamic Hill Climbing"[4]. It is a direct search method recommended for those problems in which the objective and/or constraint function are very noisy, or when the gradient information is difficult to approximate accurately. It can be regarded as a more robust choice for those problems where nl2sol fails or performs badly.

Regarding the settings associated to local solvers in scatter search, in addition to the already commented *local.balance* and *local.n2*, the following are relevant:

- *local.n1*: Number of iterations before the first call to the local solver.

- *local.tol*: tolerance in local search.

- *evaluation threshold*: maximum number of evaluations to be performed in the local solver.

Default values of these settings are detailed in saCeSS package (see Section 1)).

3 Measurement methodology and reported results

Both in the manuscript and in this supplementary info, we present a comparative performance analysis of the proposed saCeSS method with respect to several other parallel eSS schemes. The different parallel scatter search schemes compared are:

- ***np*-eSS:** where a specific *np* instances of sequential scatter search are performed in parallel without cooperation among them.
- **CeSS:** a parallel cooperative eSS scheme where different sequential eSS exchange information synchronously based on a time elapse [1].
- **saCeSS(worst)**: a parallel cooperative eSS scheme similar to the one proposed, where different sequential eSS exchange information driven by quality of the solution, but that replace the *worst* entry of the *RefSet* with the incoming cooperative solutions, and without the self-adaptive mechanism to tune the settings of the different eSS processes.
- **saCeSS(coop)**: a parallel cooperative eSS scheme similar to the one proposed, where different sequential eSS exchange information driven by quality of the solution, and that replace only one labeled *cooperative* entry of the *RefSet* with the incoming solutions, but without the self-adaptive mechanism to tune the settings of the different eSS processes.
- **saCeSS**: the novel, more competitive, parallel self-adaptive eSS scheme proposed.

Note that all these schemes are parallel, including the *np*-eSS. When executing *np*-eSS, *np* different sequential eSS threads are running in parallel without cooperation. This allows for the evaluation of the impact of the parallel cooperation in the cooperative schemes.

The speedups reported in the paper are calculated as Sp=Tref/T, where Tref is the execution time of the algorithm of reference, most of the time the *np*-eSS. Note that this is not the classical way of calculate the speedup in parallel algorithms, where usually the Tref corresponds to the execution time of the serial algorithm running in one processor. Results of the execution time for the serial eSS running in one processor would be much larger and present a larger dispersion, as it can be seen in Figure 1. Thus, speedups calculated versus the serial algorithm will be much larger than the speedups reported in this manuscript.

Figure 1 shows the results obtained for the serial eSS algorithm running on a single processor, the parallel non cooperative *np*-eSS algorithm running on 10 processors and reporting the best execution time of those 10 independent runs, and the saCeSS proposed using 10 processors. Note the logarithmic scale used in Y-axis. As can be observed, the dispersion of the sequential execution is huge for the B2 problem, presenting also outliers. However, when the *10*-eSS algorithm is executed the dispersion drastically reduces and the outliers

disappear. This is because, for each test, we are running 10 independent eSS algorithms and discarding all the results from the distribution obtained except the best one. We provide in the manuscript results for *np*-eSS because we truly believe that this represents a more honest comparison with saCeSS. If a researcher has 10 free cores to run his/her problem, he/she can use those 10 cores even if he/she only has a sequential version of the algorithm implemented and keep the best result. In previous figure note that the saCeSS, thanks to the cooperation between islands, obtained most of the execution times in the low part of the distribution, significatively improving the *10*-eSS results.

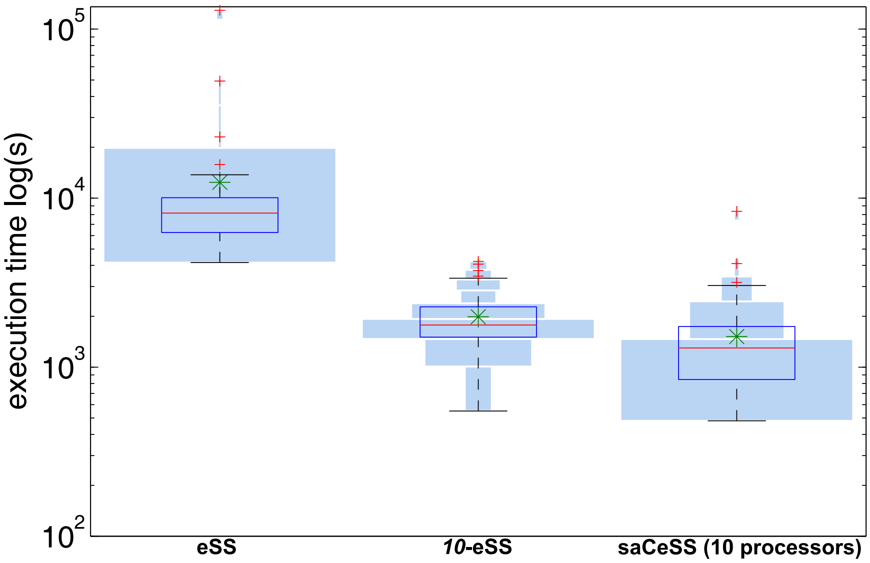

Figure 1: Violin/Box plots of execution time for the serial eSS running on 1 processor, *10*-eSS and the saCeSS proposed using 10 cores. Benchmark B2, VTR=250 and number of runs=70

Assessing the performance of metaheuristics is not an easy task, due to the stochastic nature of these methods and the substantial dispersion of computational results. Thus, for each experiment reported, 20 runs were performed and a statistical analysis was carried out. The number of executions (20) was decided based on a pragmatic reason. The complexity of some problems such as B3 causes a very large calculation times, difficulting for these cases the performing of a large number of runs in a reasonable time (note that we are limited by the hardware availability of the local cluster). In most of the BioPreDyn benchmarks these number of samples suffices to accurately analyse the performance of the proposal method. However, for B2 problem a reasonable doubt arise since in the first 20 runs an outlier appears when executing saCeSS method in 10 processors. Thus, in order to determine if 20 samples suffices also for B2, we have performed 50 extra runs (i.e. 70 samples in total) for this benchmark and

saCeSS method using 10, 20 and 40 processors. Figure 2 shows a violin/boxplot with the results obtained.

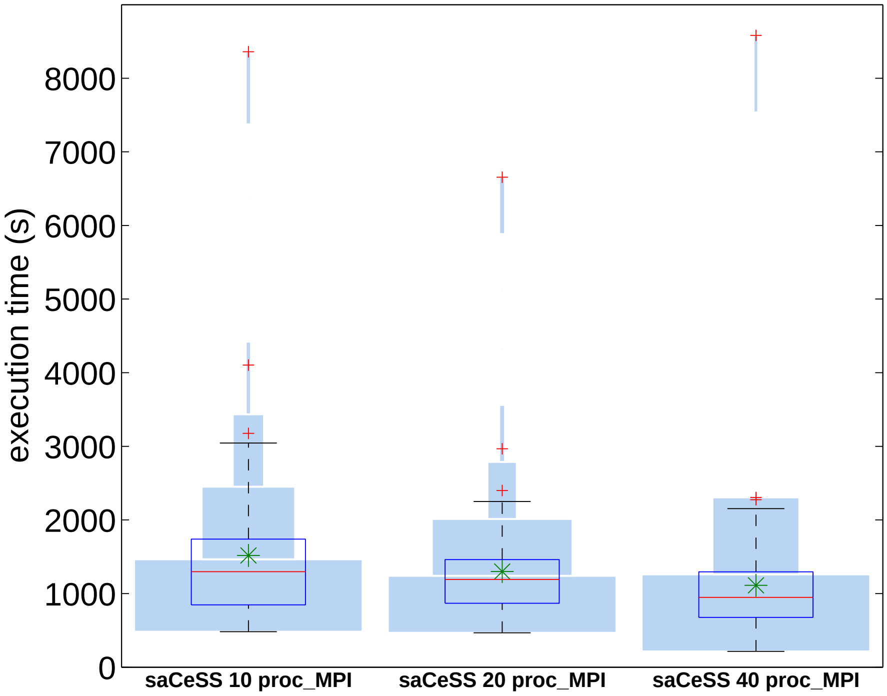

Figure 2: Violin/Box plots of execution time for saCeSS using 10, 20 and 40 cores. Benchmark B2, VTR=250 and number of runs=70.

As can be seen, the dispersion obtained is similar to the one reported in the manuscript for 20 runs each. In general, one outlier appeared in each experiment during the 70 runs, which, on our opinion, can be considered a very low frequency. Also, the mean execution time obtained after 70 runs is as reported in table 1. Although the results for 70 runs are slightly better than those for only 20 runs, we can conclude that 20 runs suffices to test and compare these metaheuristics for the BioPreDyn set.

Table 1: **Comparative mean execution time for saCeSS**

**#islands**	**70 runs**	**20 runs (reported in the manuscript)**
10	1518 ± 1091 (s)	1694 ± 1677 (s)
20	1299 ± 818 (s)	1345 ± 619 (s)
40	1111 ± 1004 (s)	1326 ± 1764 (s)

4 Performance comparison among different parallel eSS schemes

In this section we perform, for all the benchmarks in the BioPreDyn suite, a comparative performance analysis of the different parallel scatter search schemes described in previous section, using 10 processors for each of the tests.

Benchmark B1

Figures 3, 4, 5, 7 and 6 show the convergence curves for each parallel scheme compared with the proposed saCeSS. Each figure illustrates the region between the lower and upper bounds of the 20 runs performed for each experiment, with a strong line representing the median value for each time moment. For Figures 4 and 5 parameter τ specifies the time elapse between information sharing among different processes.

For comparison purposes the convergence curves of each method which are closer to the median values of the results distribution are shown the Figure 8.

When evaluating stochastic optimization solvers, it is important to take into account the dispersion of the experimental results. Figure 9 illustrates how the proposed saCeSS method reduces the variability of the execution time compared to the other parallel schemes. In addition, Table 2 details different results from these experiments, such as the mean number of evaluations needed, the mean execution time and its deviation, and the minimum and maximum execution times of the 20 independent runs.

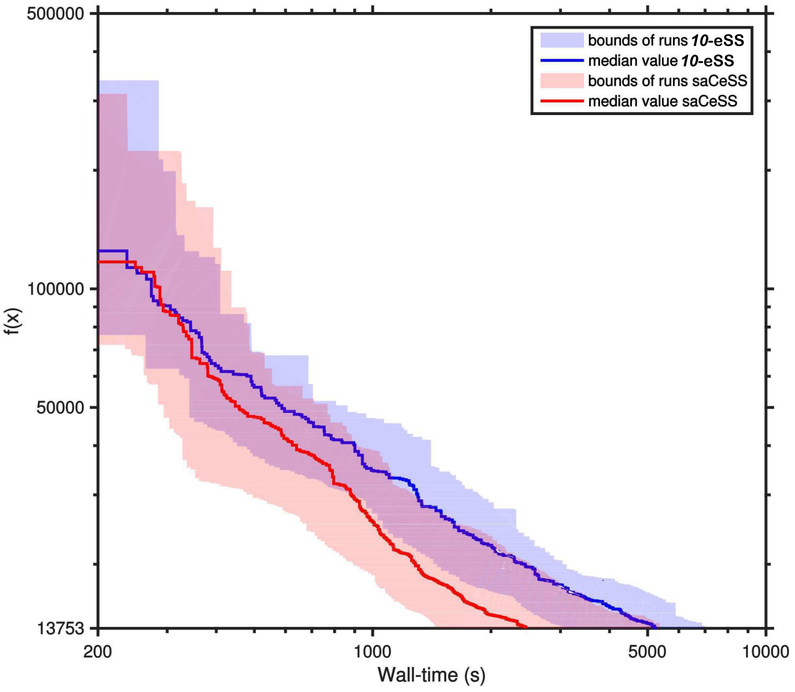

Figure 3: Convergence curves for *10*-eSS vs saCeSS considering benchmark B1.

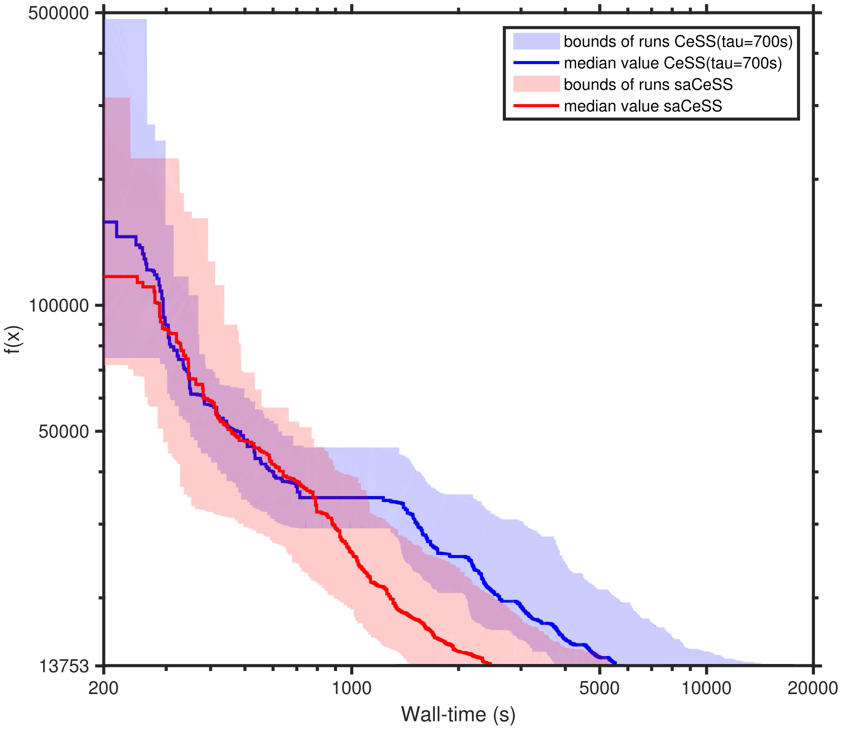

Figure 4: Convergence curves for CeSS($\tau = 700s$) vs saCeSS considering benchmark B1.

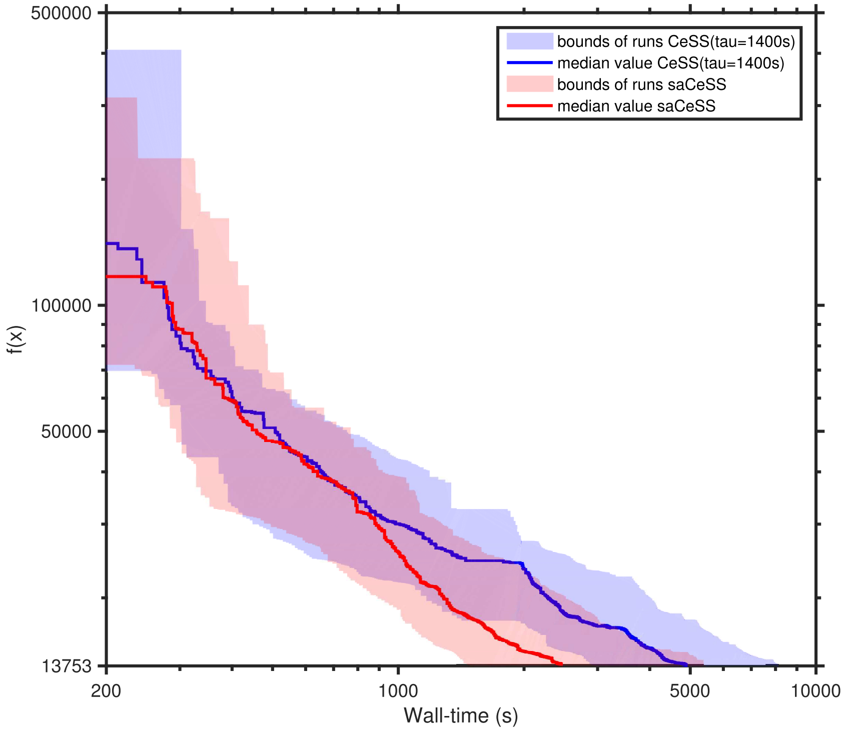

Figure 5: Convergence curves for CeSS($\tau = 1400s$) vs saCeSS considering benchmark B1.

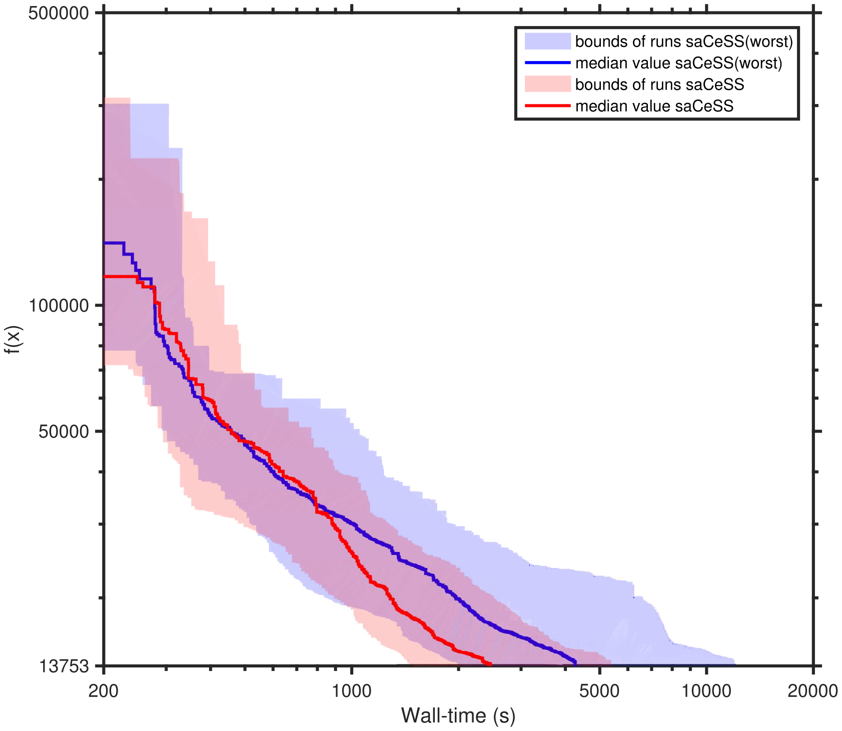

Figure 6: Convergence curves of saCeSS(worst) vs saCeSS considering benchmark B1.

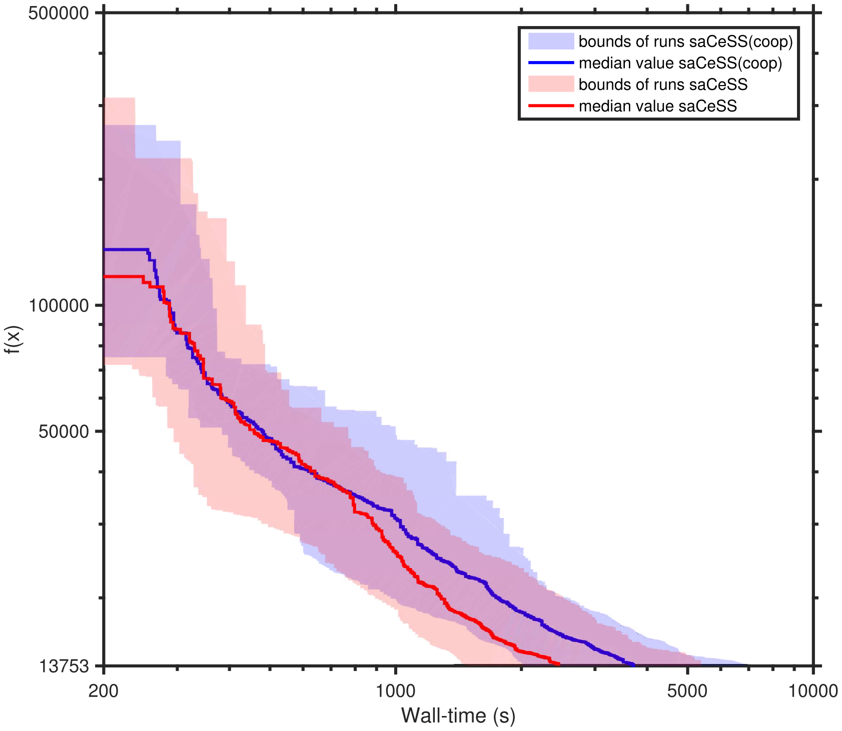

Figure 7: Convergence curves of saCeSS(coop) vs saCeSS considering benchmark B1.

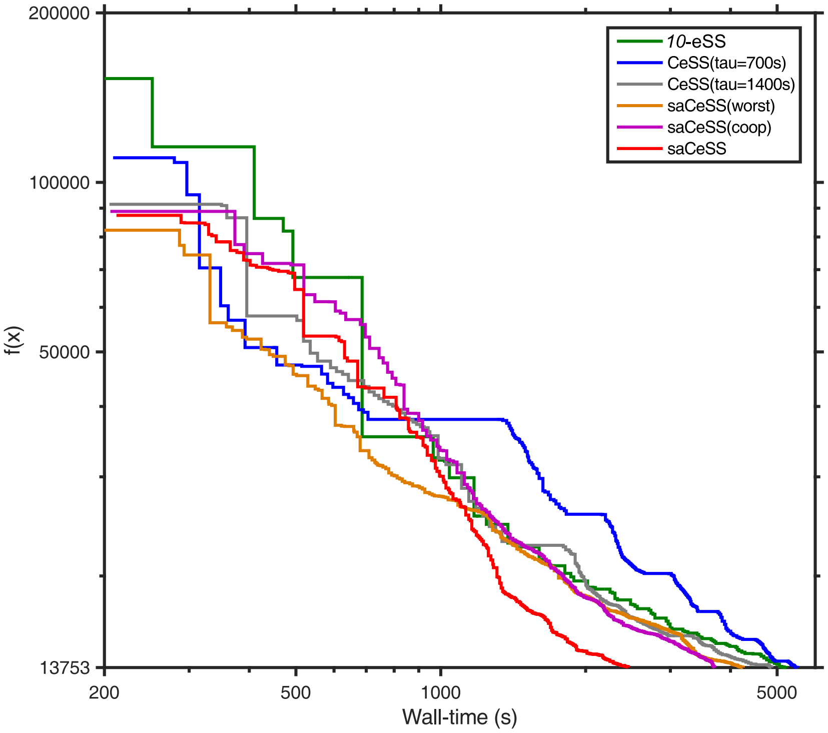

Figure 8: Convergence curves for methods *10*-eSS, CeSS and saCeSS, corresponding to the runs that are closer to the median values of the results distribution considering benchmark B1.

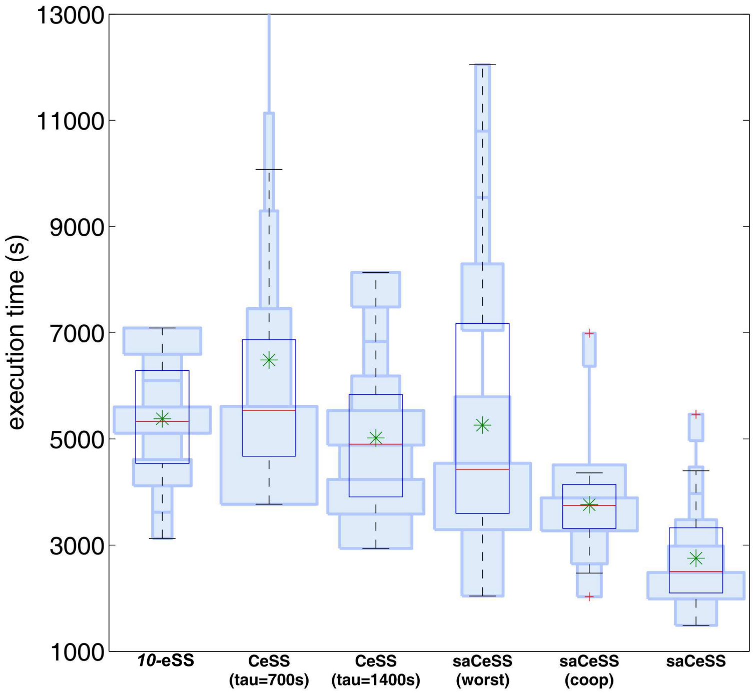

Figure 9: Violin/Box plots of execution time for different strategies in benchmark B1.

Table 2: **Detailed results for benchmark B1**

**method**	**mean iter ±std**	**mean evals ±std**	**mean time ±std(s)**	**min/max time(s)**
10-eSS	80±30	199214±44836	5378 ± 1070	3124/7090
CeSS ($\tau = 700s$)	109±49	188131±82834	6487 ± 3226	3769/18504
CeSS ($\tau = 1400s$)	122±41	175331±98255	5018 ± 1477	2936/8134
saCeSS(worst)	136±72	211742±124121	5259 ± 2640	2040/12049
saCeSS(coop)	92±35	143145±61828	3759 ± 976	2026/6991
saCeSS	62±21	92122±35058	2753 ± 955	1489/5465

Benchmark B2

Figures 10, 11, 12, 14 and 13 show the convergence curves for each parallel scheme compared with the proposed saCeSS. Each figure illustrates the region between the lower and upper bounds of the 20 runs performed for each experiment, with a strong line representing the median value for each time moment.For Figures 11 and 12 parameter τ specifies the time elapse between information sharing among different processes. For comparison purposes the convergence curves of each method for those experiments that are closer to the median values of the results distribution are shown the Figure 15. Figure 16 illustrates how the proposed saCeSS method reduces the variability of the execution time compared to the other parallel schemes. Finally Table 3 details results from these experiments.

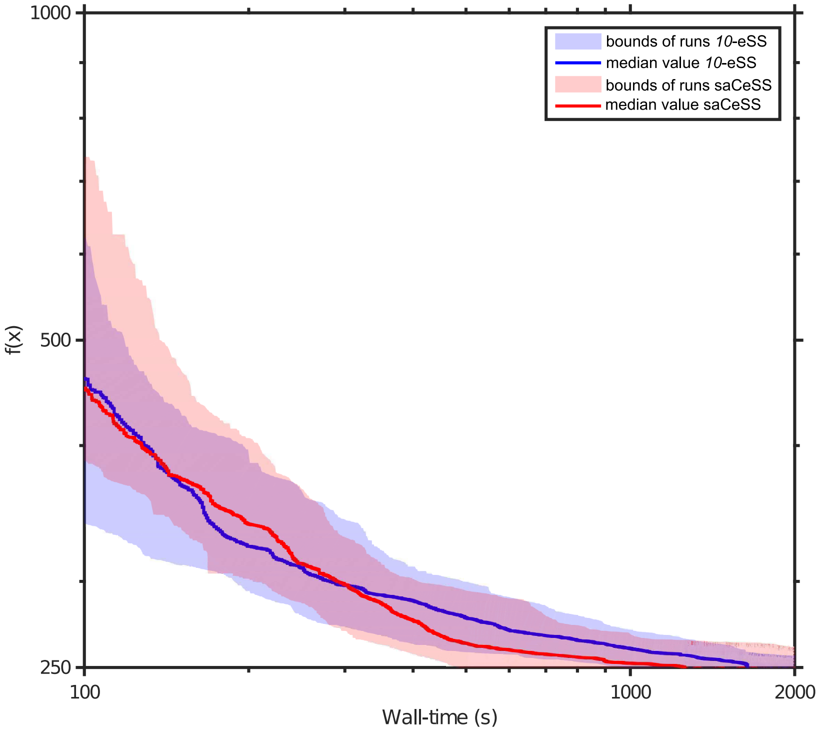

Figure 10: Convergence curves for *10*-eSS vs saCeSS considering benchmark B2.

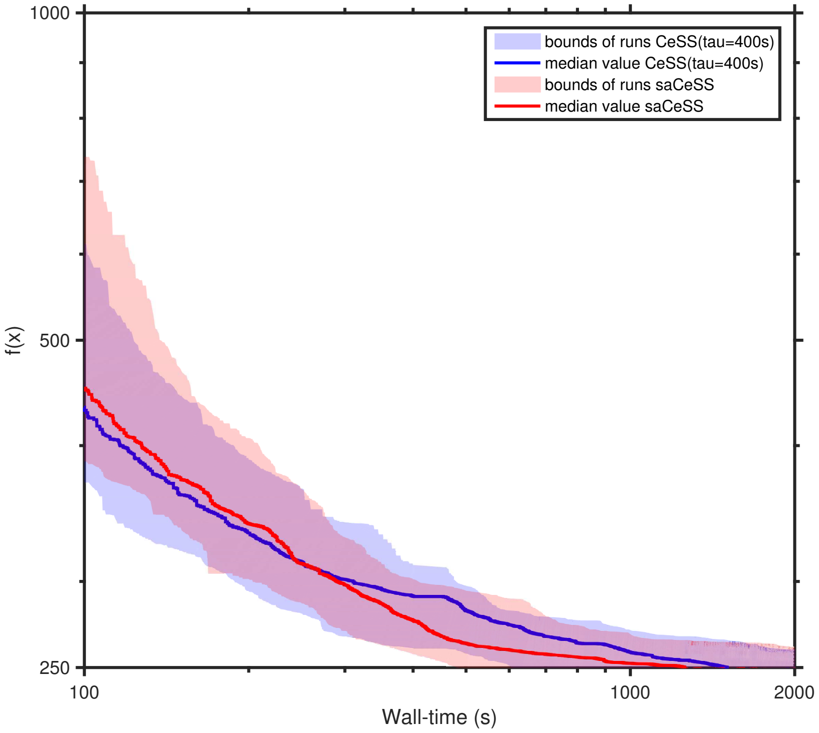

Figure 11: Convergence curves for CeSS(τ = 400s) vs saCeSS considering benchmark B2.

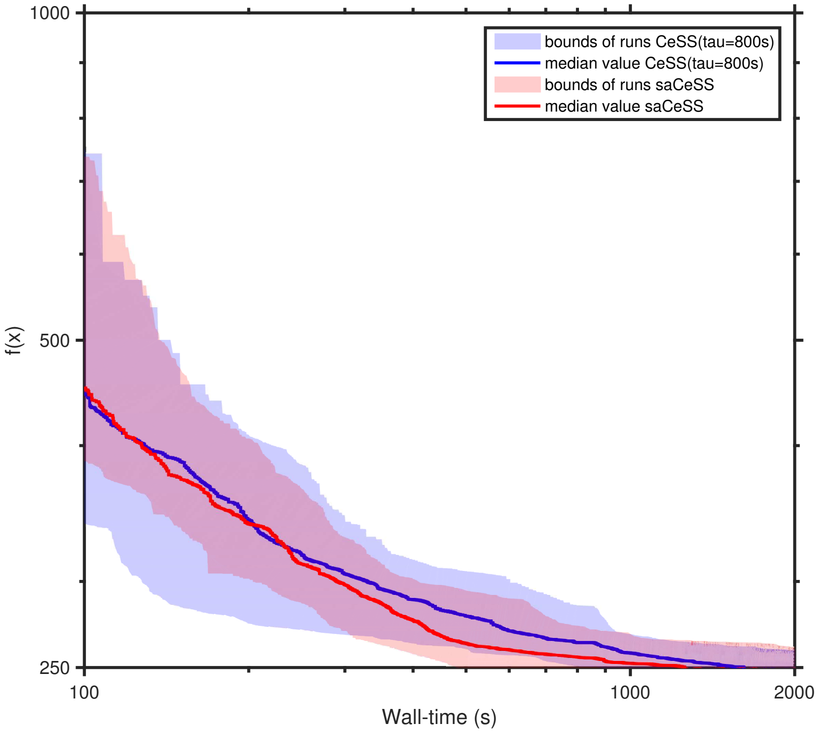

Figure 12: Convergence curves for CeSS($\tau = 800s$) vs saCeSS considering benchmark B2.

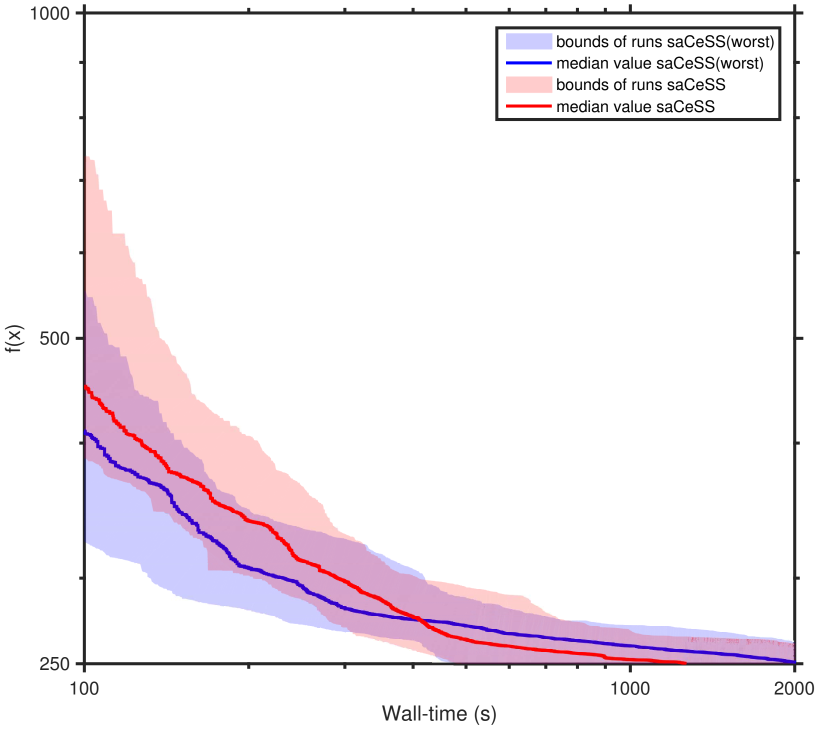

Figure 13: Convergence curves of saCeSS(worst) vs saCeSS considering benchmark B2.

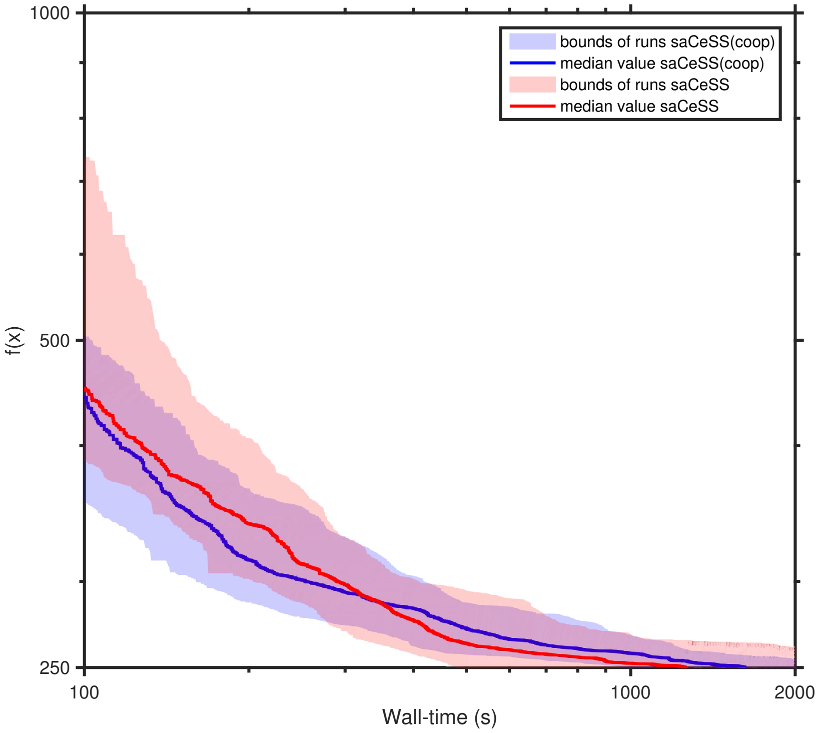

Figure 14: Convergence curves of saCeSS(coop) vs saCeSS considering benchmark B2.

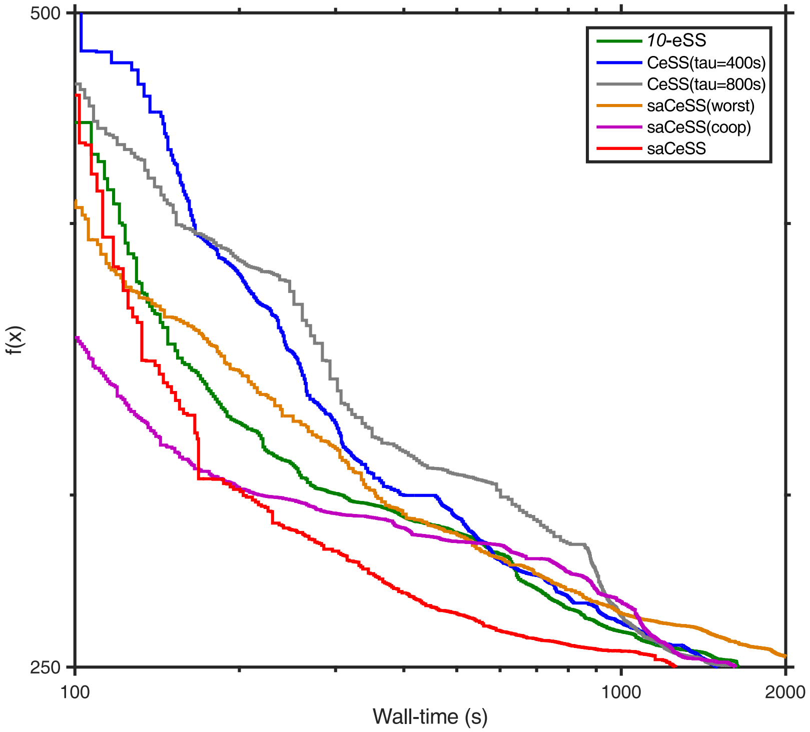

Figure 15: Convergence curves for methods *10*-eSS, CeSS and saCeSS, corresponding to the runs that are closer to the median values of the results distribution considering benchmark B2.

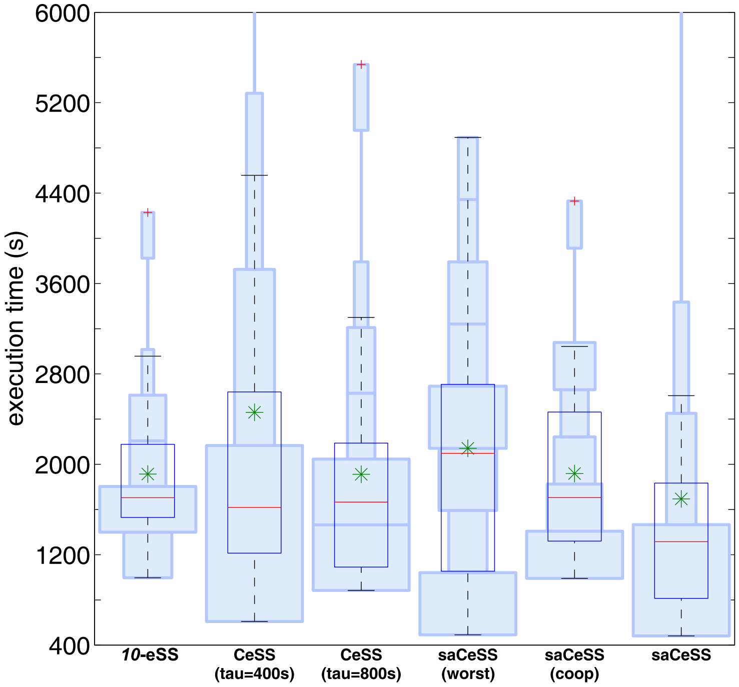

Figure 16: Violin/Box plots of execution time for different strategies in benchmark B2.

Table 3: **Detailed results for benchmark B2**

**method**	**mean iter ±std**	**mean evals ±std**	**mean time ±std(s)**	**min/max time(s)**
10-eSS	450±167	1504503±541257	1914 ± 714	995/4228
CeSS ($\tau = 400s$)	452±278	1637125±1016688	2459 ± 2705	608/13075
CeSS ($\tau = 800s$)	508±205	1802917±690613	1911 ± 1103	883/5537
saCeSS(worst)	480±268	1692240±955027	2140 ± 1206	490/4892
saCeSS(coop)	440±192	1528793±647677	1918 ± 833	990/4330
saCeSS	846±982	1247699±1222378	1694 ± 1677	481/8359

Benchmark B3

Figures 17, 18 and 19 show the convergence curves for each parallel scheme compared with the proposed saCeSS. Each figure illustrates the region between the lower and upper bounds of the 20 runs performed for each experiment, with a strong line representing the median value for each time moment. For Figure 18 parameter τ specifies the time elapse between information sharing among different processes.

For comparison purposes the convergence curves of each method for those experiments that are closer to the median values of the results distribution are shown the Figure 20.

Figure 21 illustrates how the proposed saCeSS method reduces the variability of the execution time compared to the other parallel schemes for benchmark B3. Table 4 details results from these experiments.

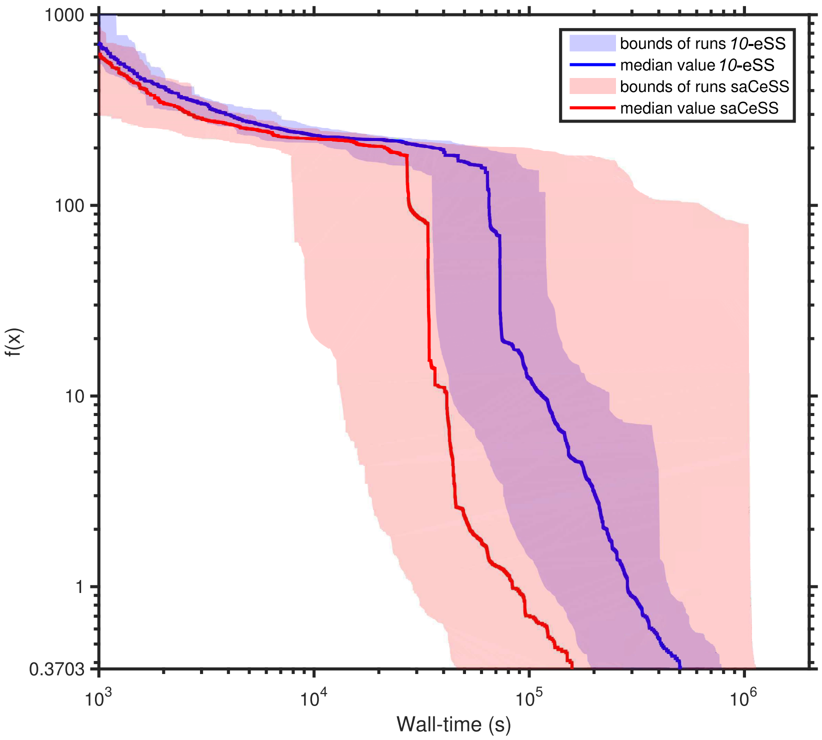

Figure 17: Convergence curves for *10*-eSS vs saCeSS considering benchmark B3.

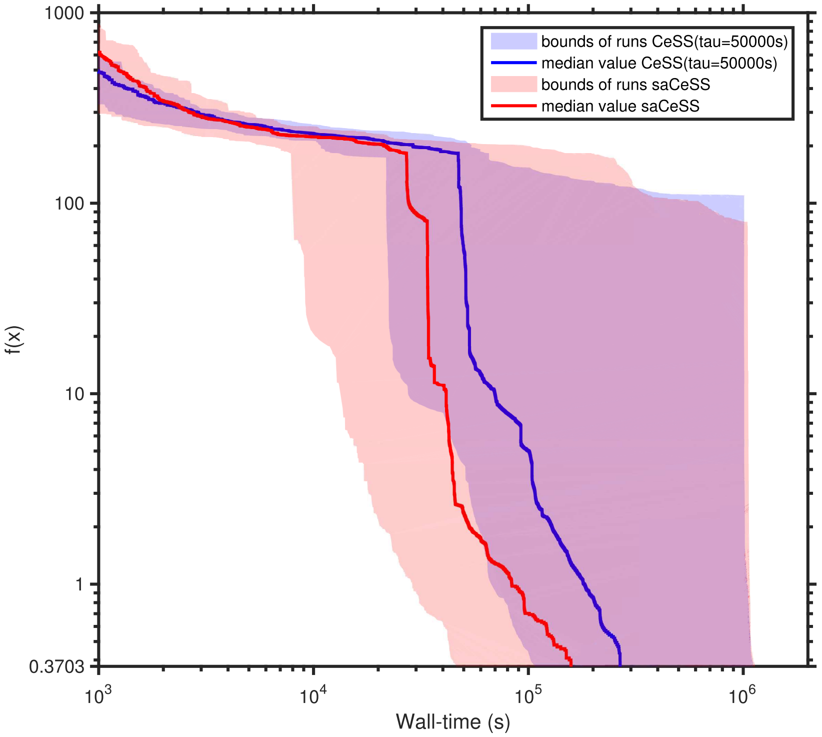

Figure 18: Convergence curves for CeSS($\tau = 50000s$) vs saCeSS considering benchmark B3.

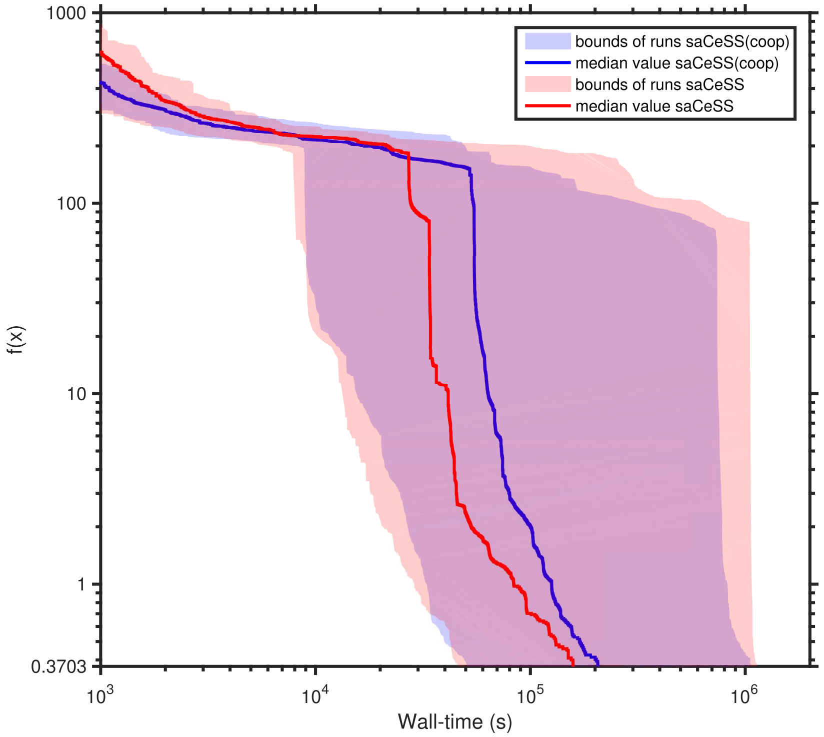

Figure 19: Convergence curves of saCeSS(coop) vs saCeSS considering benchmark B3.

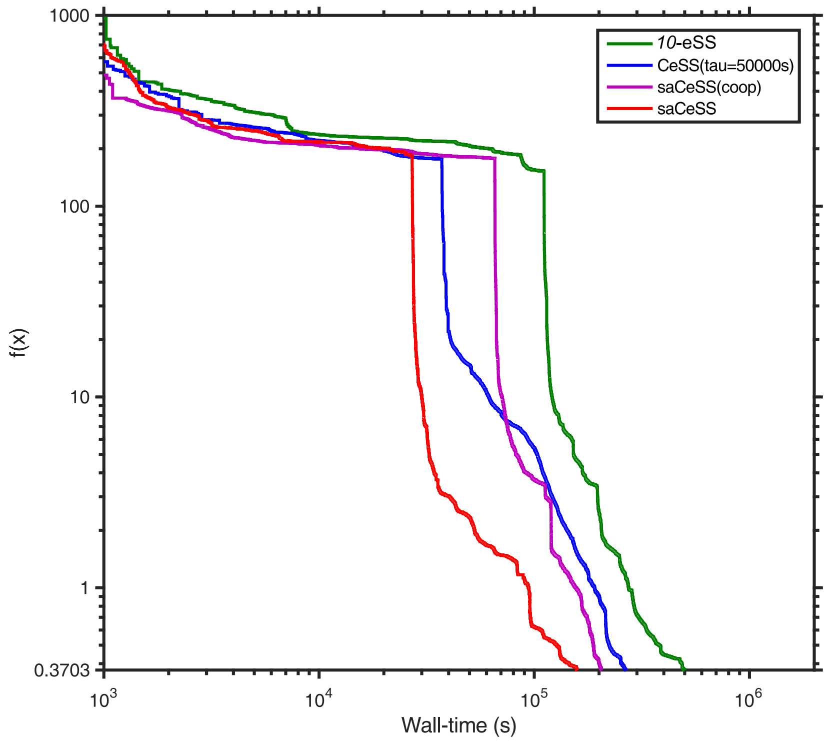

Figure 20: Convergence curves for methods *10*-eSS, CeSS and saCeSS, corresponding to the runs that are closer to the median values of the results distribution considering benchmark B3.

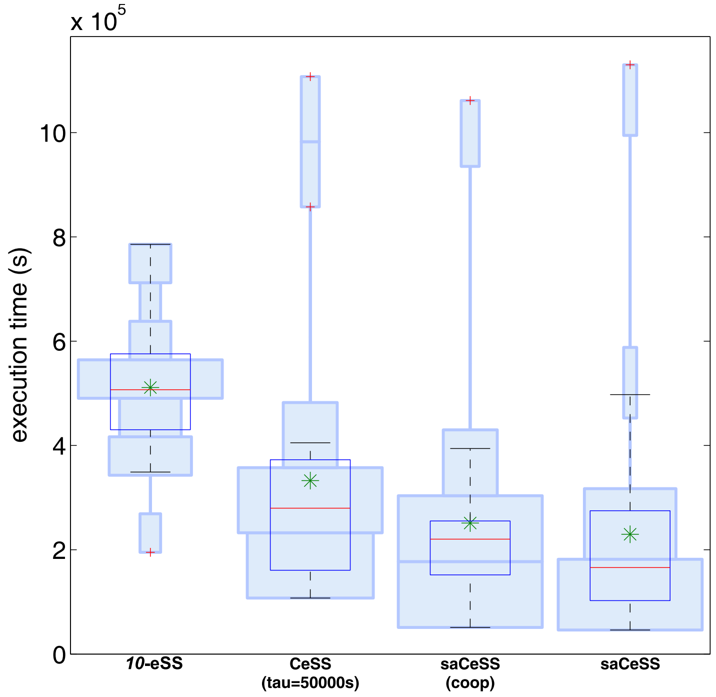

Figure 21: Violin/Box plots of execution time for different strategies in benchmark B3.

Table 4: **Detailed results for benchmark B4**

**method**	**mean iter ±std**	**mean evals ±std**	**mean time ±std(s)**	**min/max time(s)**
10-eSS	10062±2528	66915128±15623835	511166 ± 135988	195256/785777
CeSS($\tau = 50000s$)	7288±5551	52592578±35513874	332721 ± 245829	107526/1107389
saCeSS(coop)	4323±3251	32604331±23357322	251305 ± 209082	51185/1061604
saCeSS	4113±3130	27647470±21488783	229888 ± 238970	46290/1129976

Benchmark B4

Figures 22, 23, 24, 26 and 25 show the convergence curves for each parallel scheme compared with the proposed saCeSS. Each figure illustrates the region between the lower and upper bounds of the 20 runs performed for each experiment, with a strong line representing the median value for each time moment.For Figures 23 and 24 parameter τ specifies the time elapse between information sharing among different processes.

For comparison purposes the convergence curves of each method for those experiments that are closer to the median values of the results distribution are shown the Figure 27.

Figure 28 illustrates how the proposed saCeSS method reduces the variability of the execution time compared to the other parallel schemes for benchmark B4. Table 5 details results from these experiments.

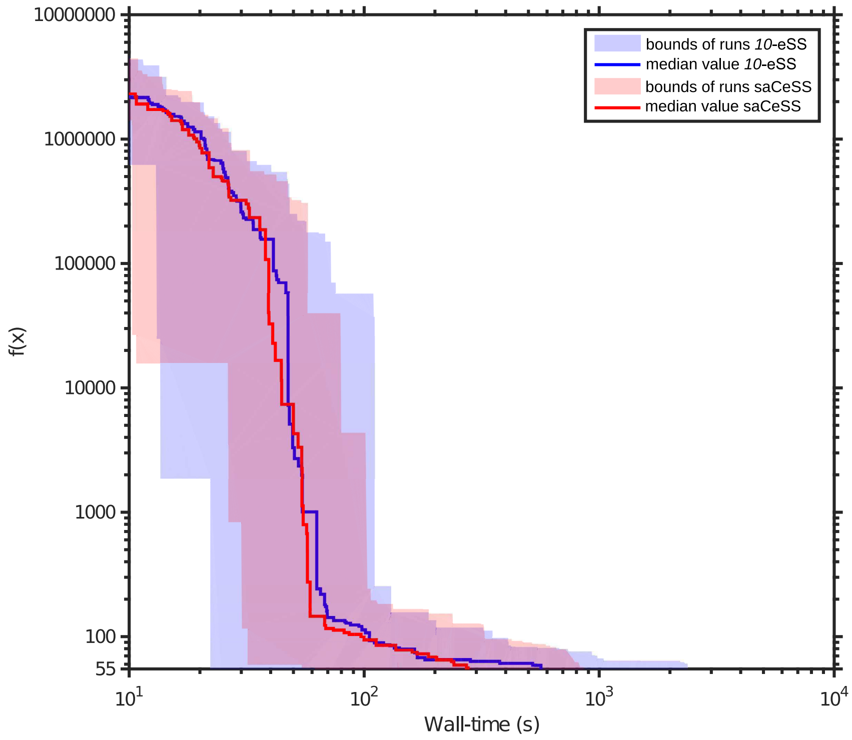

Figure 22: Convergence curves for *10*-eSS vs saCeSS considering benchmark B4.

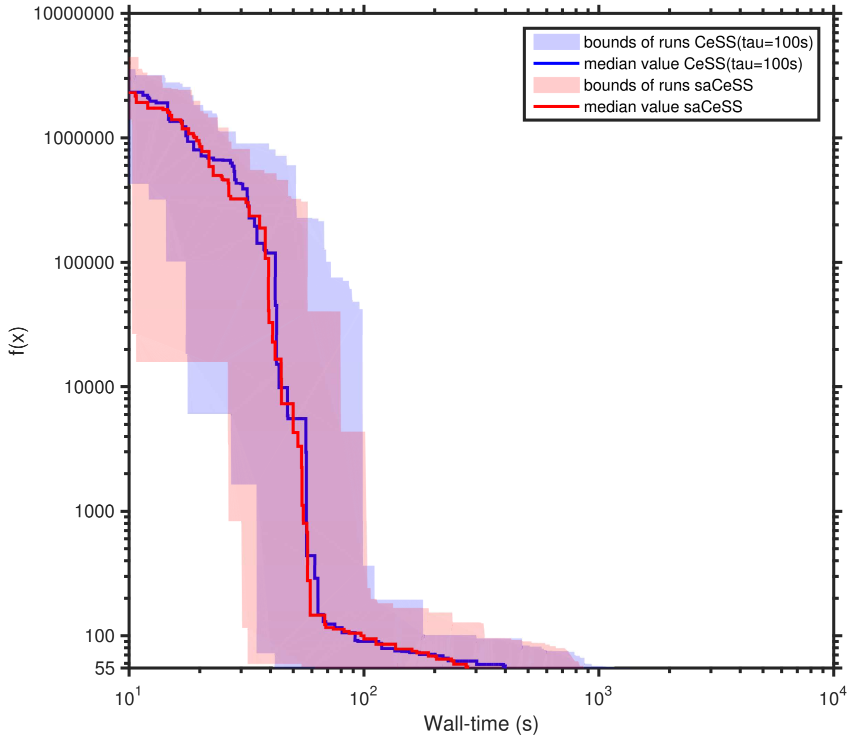

Figure 23: Convergence curves for CeSS($\tau = 100s$) vs saCeSS considering benchmark B4.

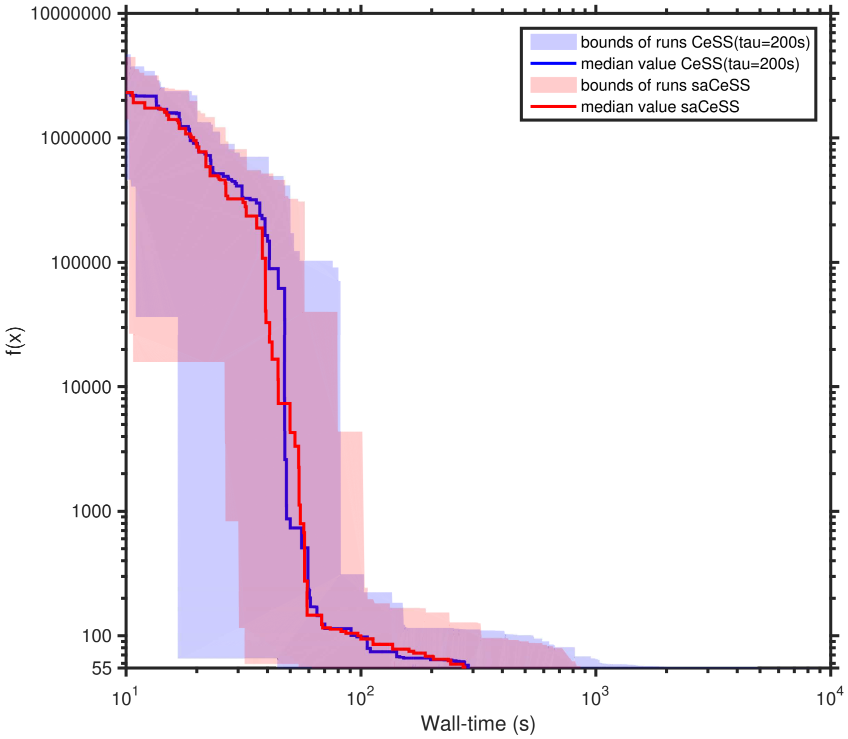

Figure 24: Convergence curves for CeSS($\tau = 200s$) vs saCeSS considering benchmark B4.

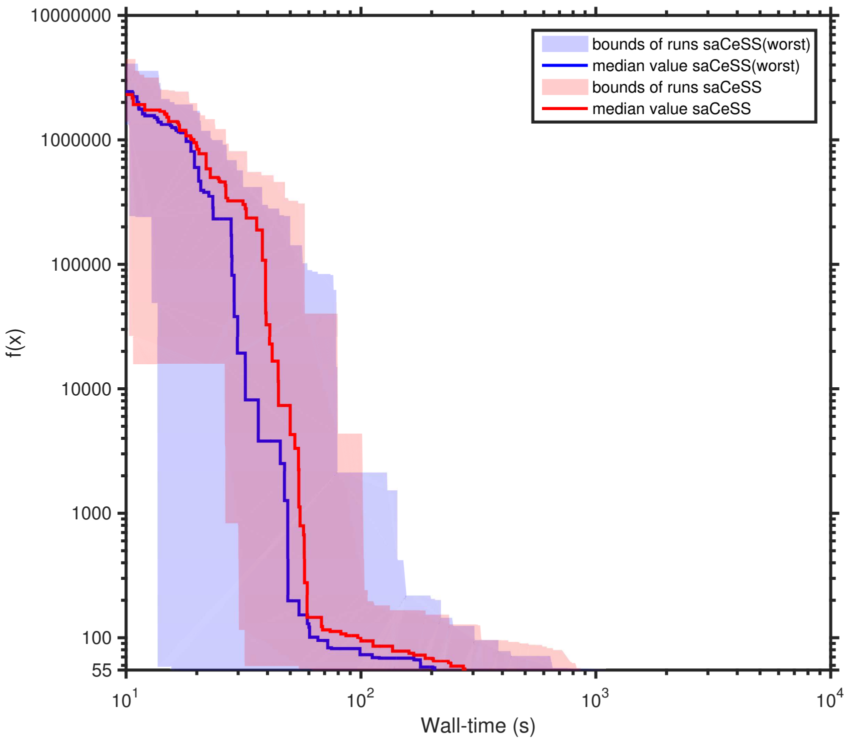

Figure 25: Convergence curves of saCeSS(worst) vs saCeSS considering benchmark B4.

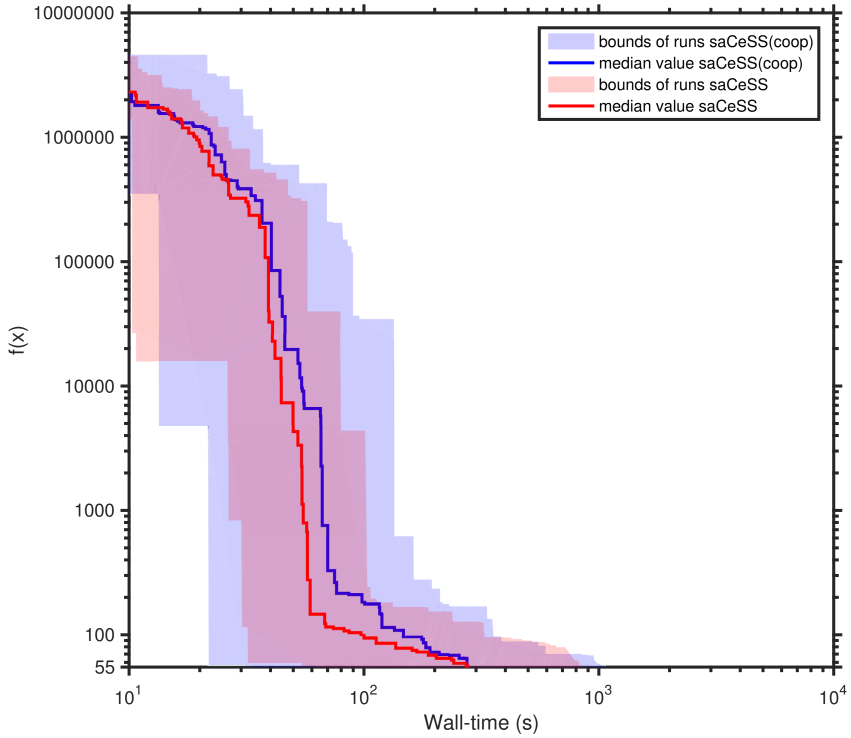

Figure 26: Convergence curves of saCeSS(coop) vs saCeSS considering benchmark B4.

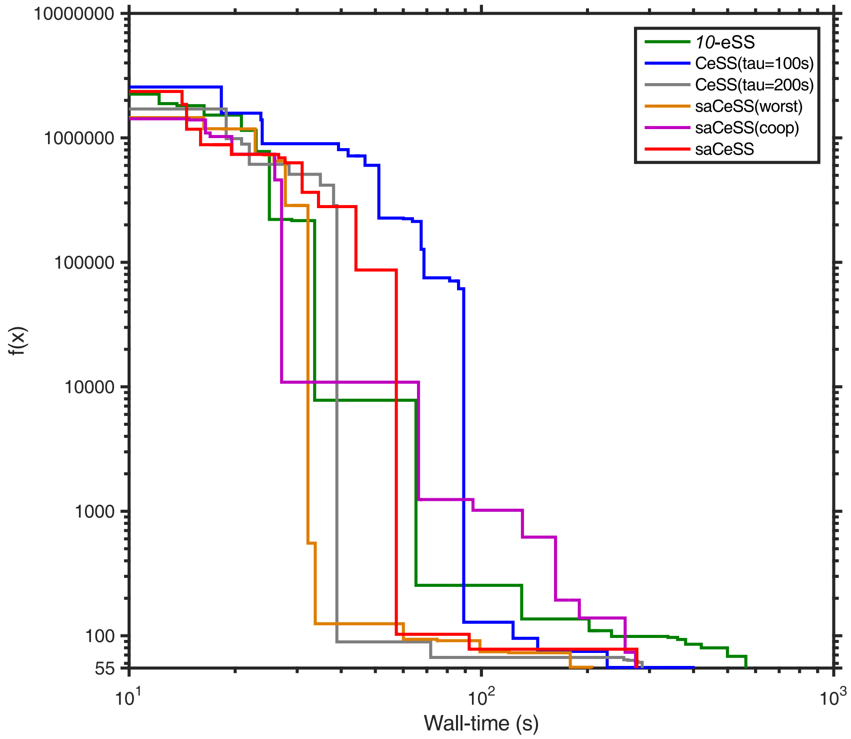

Figure 27: Convergence curves for methods *10*-eSS, CeSS and saCeSS, corresponding to the runs that are closer the median values of the results distribution considering benchmark B4.

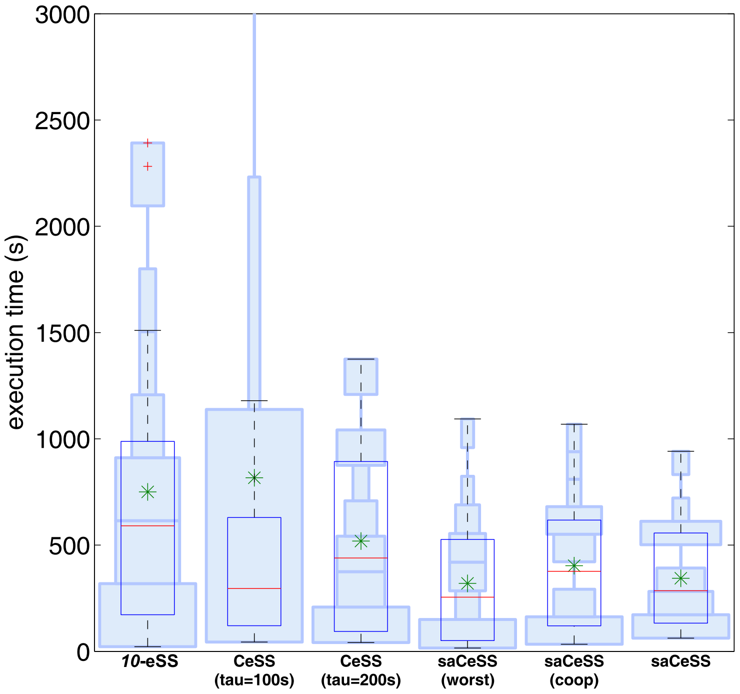

Figure 28: Violin/Box plots of execution time for different strategies in benchmark B4.

Table 5: **Detailed results for benchmark B4**

**method**	**mean iter ±std**	**mean evals ±std**	**mean time ±std(s)**	**min/max time(s)**
eSS	99±121	2230089±2068300	750 ± 692	22/2392
CeSS ($\tau = 100s$)	140±386	1665954±2921838	817 ± 1909	44/8796
CeSS ($\tau = 200s$)	119±87	1649723±1024833	518 ± 428	41/1375
saCeSS(worst)	34±34	947565±914204	319 ± 297	15/1093
saCeSS(coop)	39±30	1163458±927751	402 ± 303	33/1068
saCeSS	35±24	1017956±728328	343 ± 240	62/941

Benchmark B5

Figures 29, 30, 31, 33 and 32 show the convergence curves for each parallel scheme compared with the proposed saCeSS. Each figure illustrates the region between the lower and upper bounds of the 20 runs performed for each experiment, with a strong line representing the median value for each time moment.For Figures 30 and 31 parameter τ specifies the time elapse between information sharing among different processes.

For comparison purposes the convergence curves of each method for those experiments that are closer to the median values of the results distribution are shown the Figure 34.

Figure 35 illustrates how the proposed saCeSS method reduces the variability of the execution time compared to the other parallel schemes for benchmark B5. In addition, Table 6 details different results from these experiments.

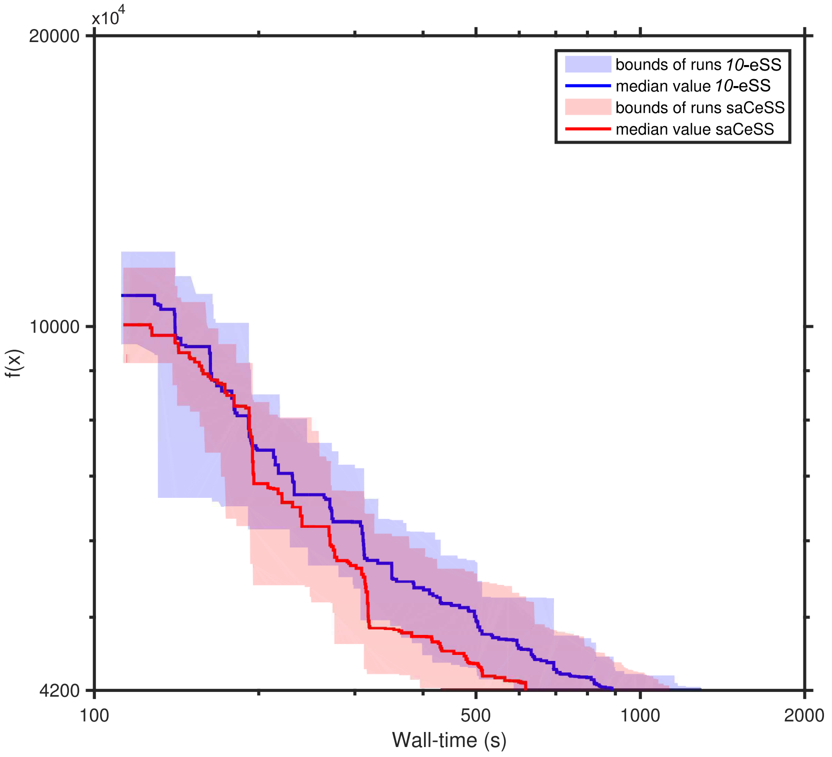

Figure 29: Convergence curves for *10*-eSS vs saCeSS considering benchmark B5.

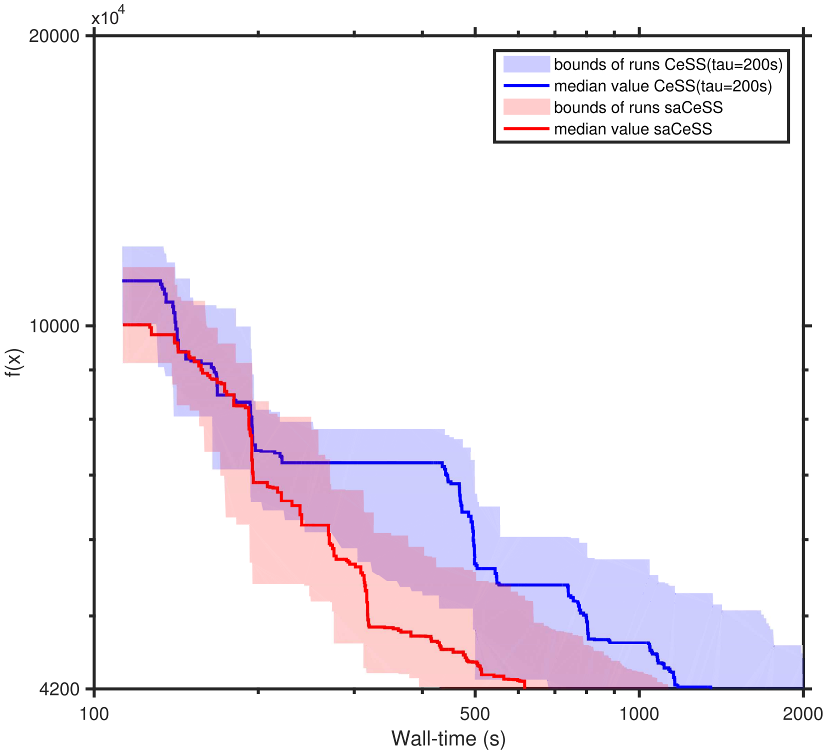

Figure 30: Convergence curves for CeSS(τ = 200) vs saCeSS considering benchmark B5.

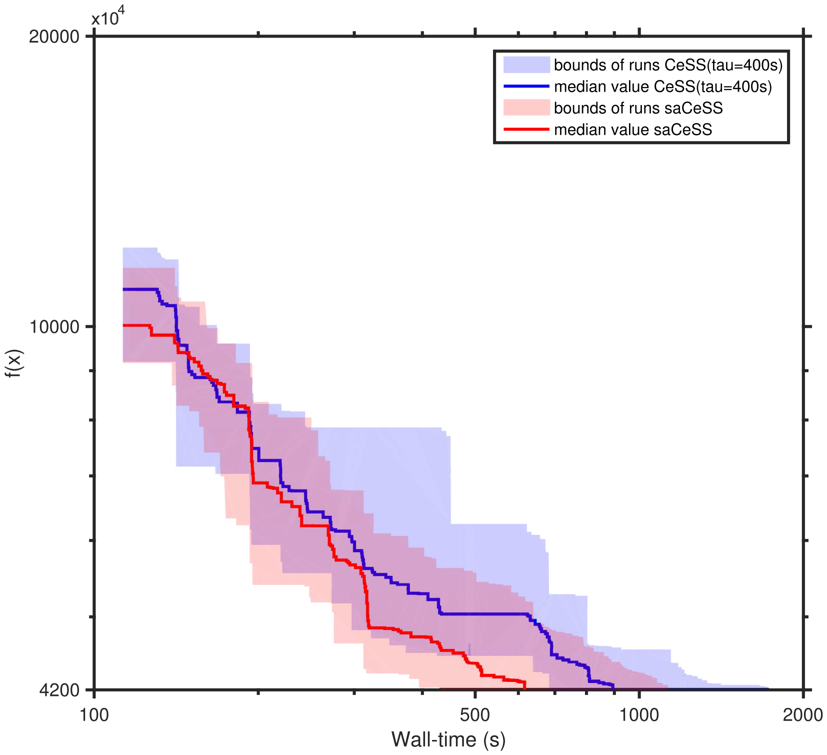

Figure 31: Convergence curves for CeSS($\tau = 400s$) vs saCeSS considering benchmark B5.

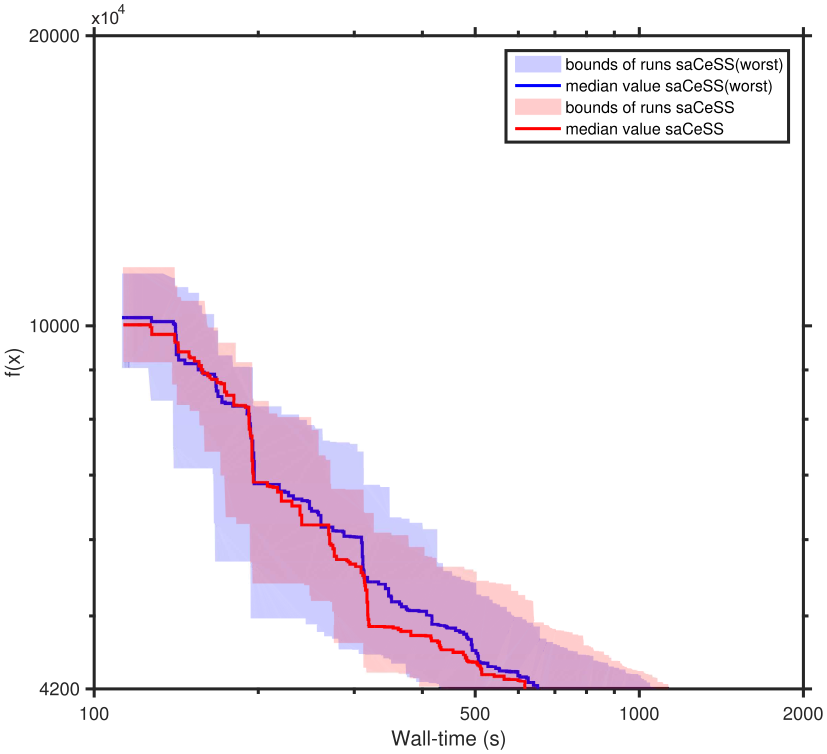

Figure 32: Convergence curves of saCeSS(worst) vs saCeSS considering benchmark B5.

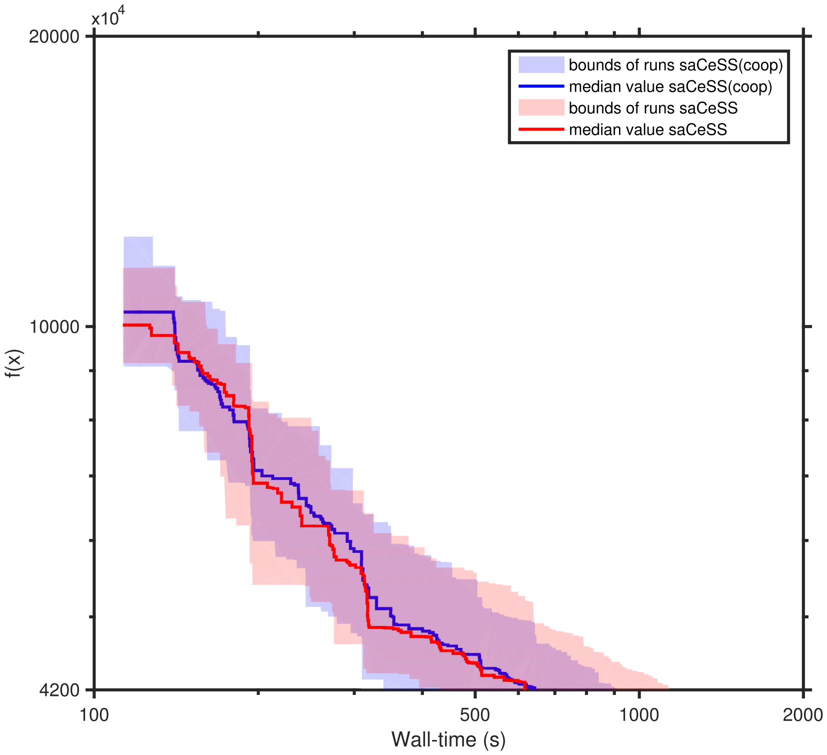

Figure 33: Convergence curves of saCeSS(coop) vs saCeSS considering benchmark B5.

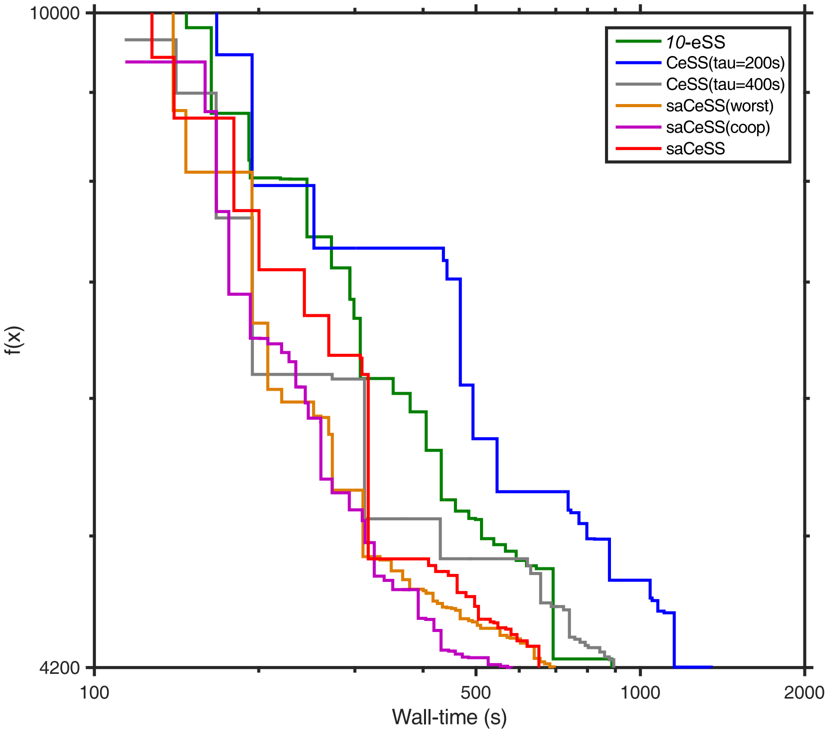

Figure 34: Convergence curves for methods *10*-eSS, CeSS and saCeSS, corresponding to the runs that are closer to the median values of the results distribution considering benchmark B5.

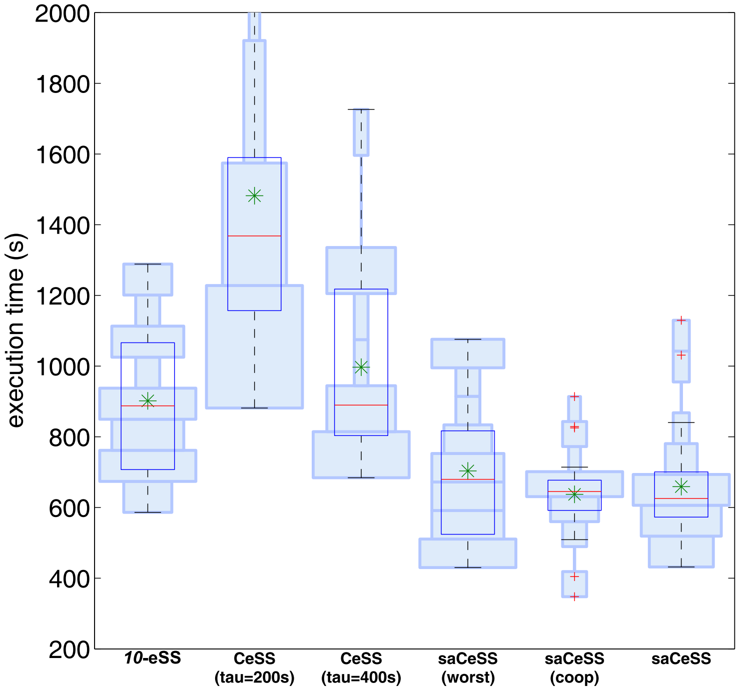

Figure 35: Violin/Box plots of execution time for different strategies in benchmark B5.

Table 6: **Detailed results for benchmark B5**

**method**	**mean iter ±std**	**mean evals ±std**	**mean time ±std(s)**	**min/max time(s)**
10-eSS	16±4	69448±14570	901 ± 197	585/1288
CeSS (τ = 200s)	11±4	108481±36190	1481 ± 634	881/3654
CeSS (τ = 400s)	14±3	94963±20172	996 ± 264	683/1726
saCeSS(worst)	11±3	54824±14741	703 ± 202	429/1075
saCeSS(coop)	10±2	49622±9530	637 ± 131	347/913
saCeSS	10±3	51076±12696	658 ± 174	431/1129

Benchmark B6

Figures 36, 37, 38, 40 and 39 show the convergence curves for each parallel scheme compared with the proposed saCeSS. Each figure illustrates the region between the lower and upper bounds of the 20 runs performed for each experiment, with a strong line representing the median value for each time moment. For Figure 37 parameter τ specifies the time elapse between information sharing among different processes.

For comparison purposes the convergence curves of each method for those experiments that are closer to the median values of the results distribution are shown the Figure 41.

Figure 42 illustrates how the proposed saCeSS method reduces the variability of the execution time compared to the other parallel schemes for benchmark B6. Table 7 details results from these experiments.

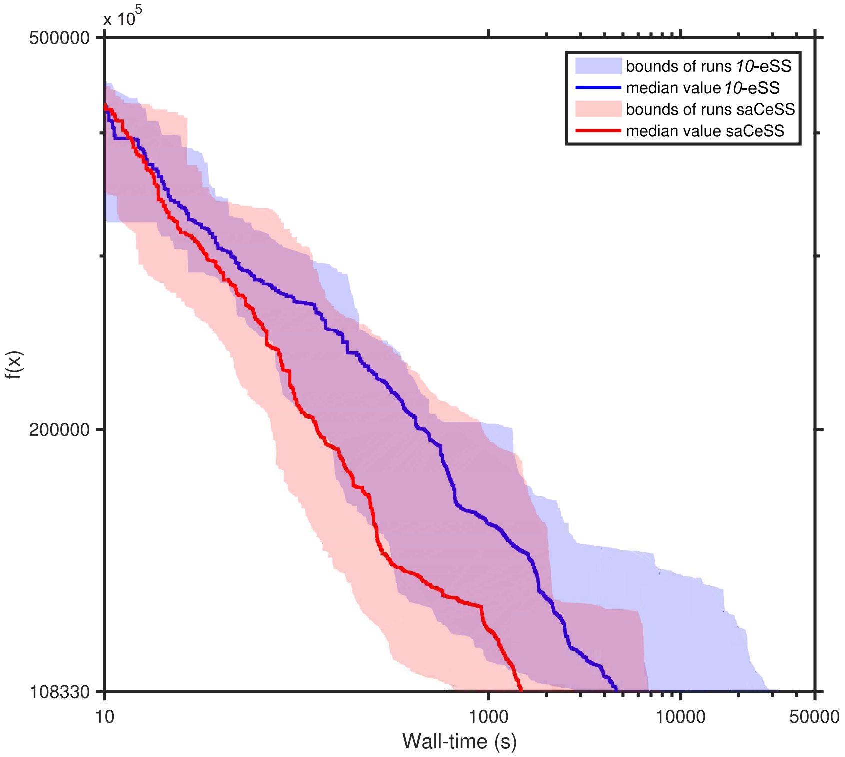

Figure 36: Convergence curves for *10*-eSS vs saCeSS considering benchmark B6.

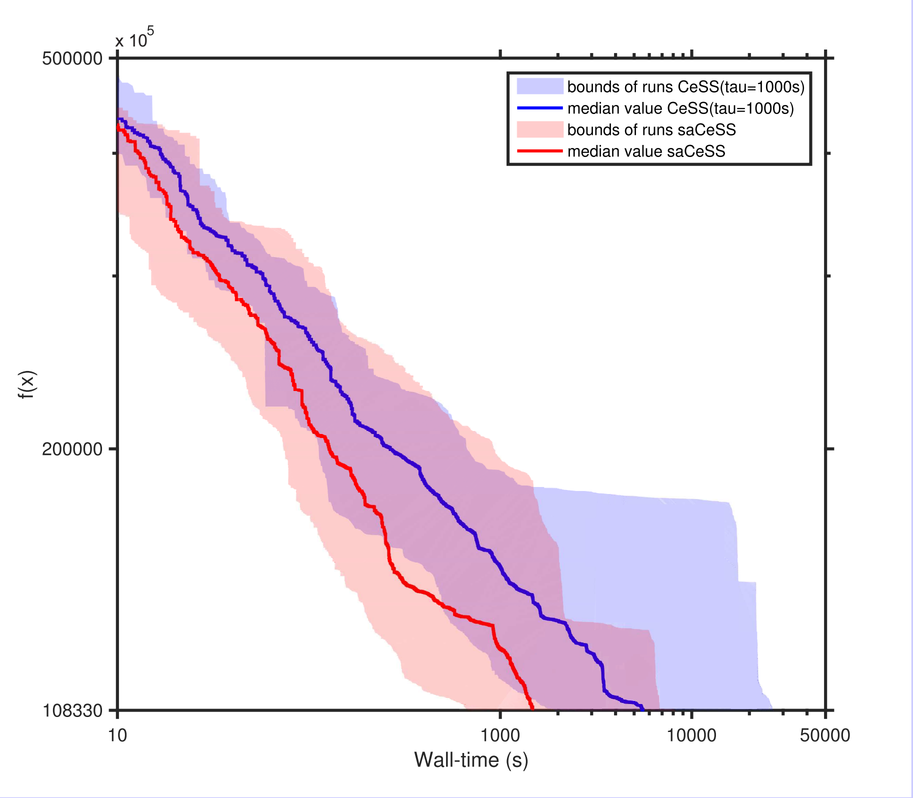

Figure 37: Convergence curves for CeSS($\tau = 1000s$) vs saCeSS considering benchmark B6.

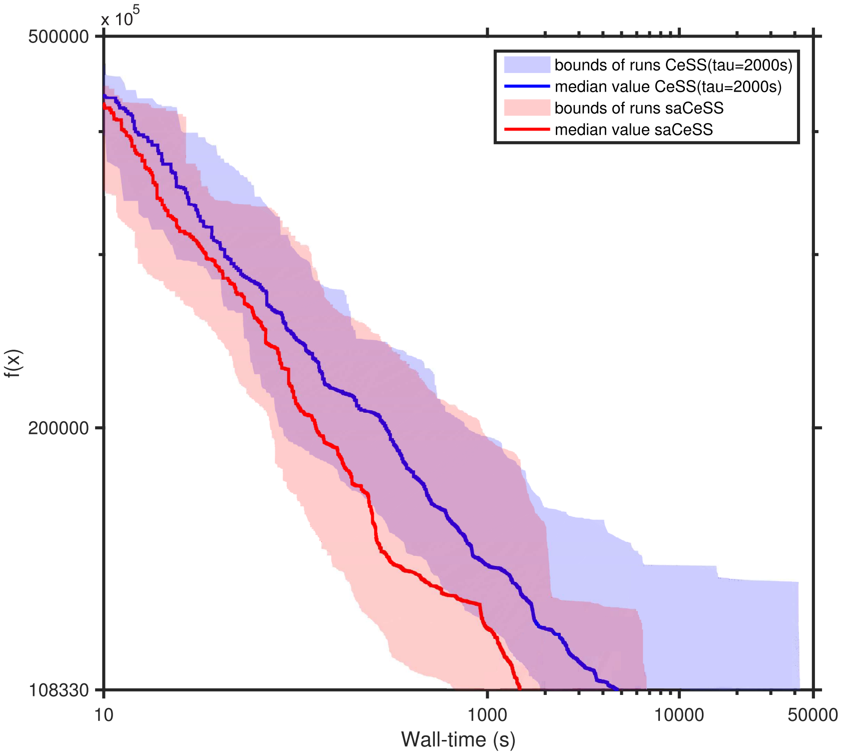

Figure 38: Convergence curves for CeSS($\tau = 2000s$) vs saCeSS considering benchmark B6.

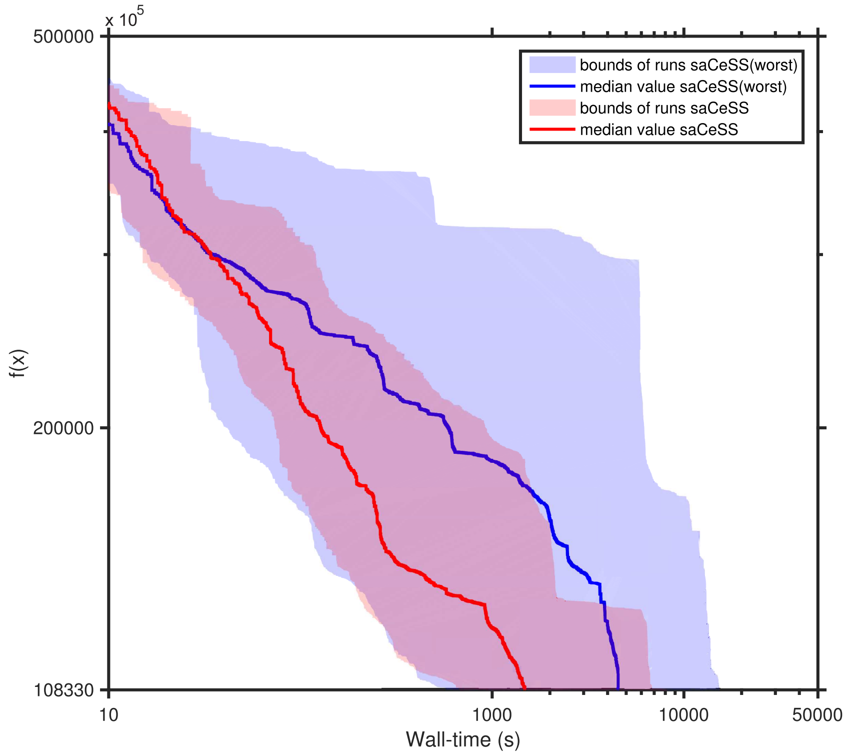

Figure 39: Convergence curves of saCeSS(worst) vs saCeSS considering benchmark B6.

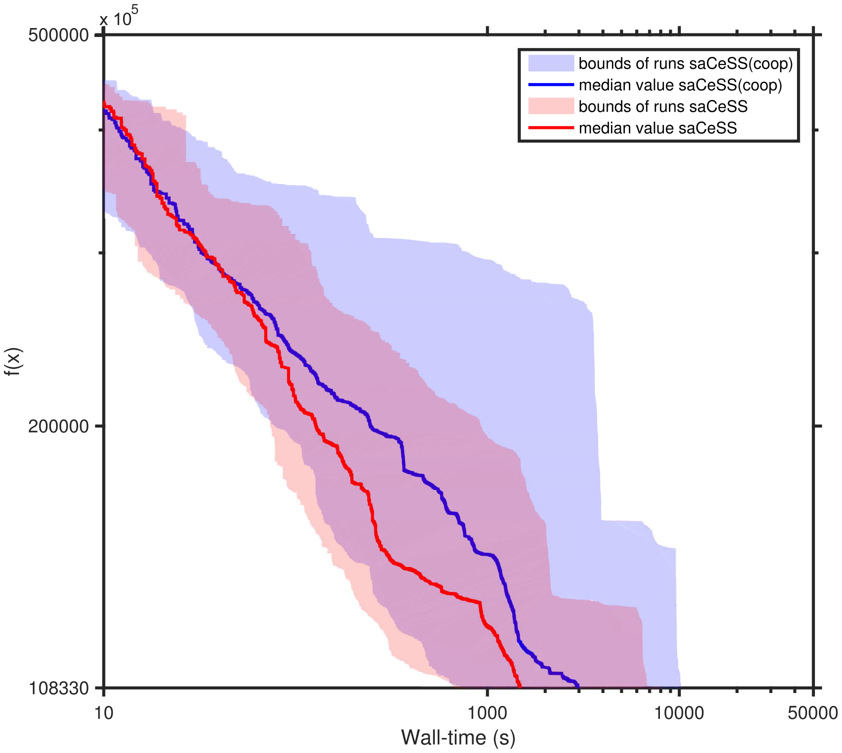

Figure 40: Convergence curves of saCeSS(coop) vs saCeSS considering benchmark B6.

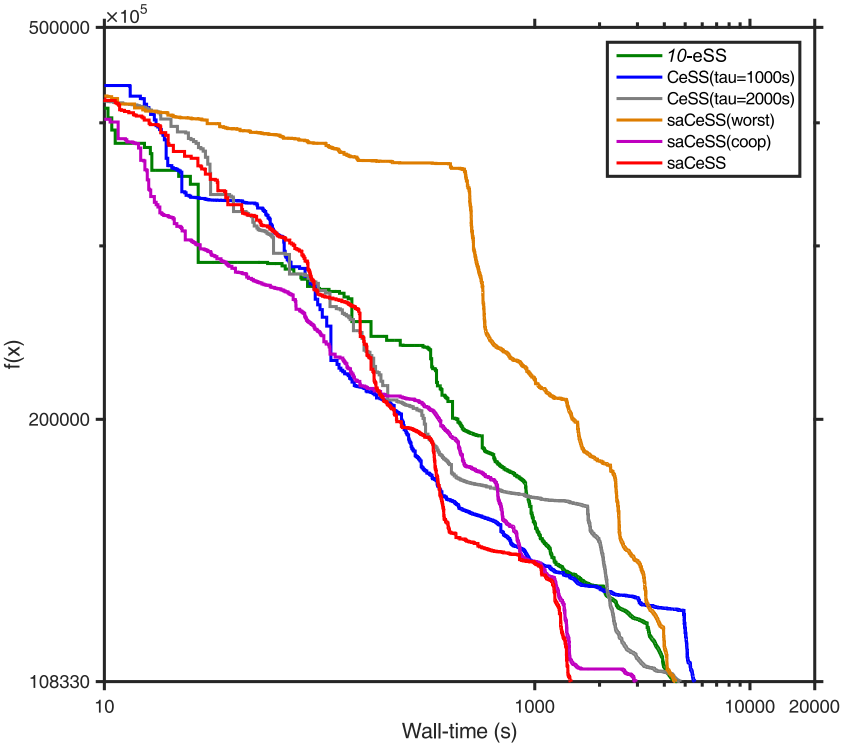

Figure 41: Convergence curves for methods *10*-eSS, CeSS and saCeSS, corresponding to the runs that are closer to the median values of the results distribution considering benchmark B6.

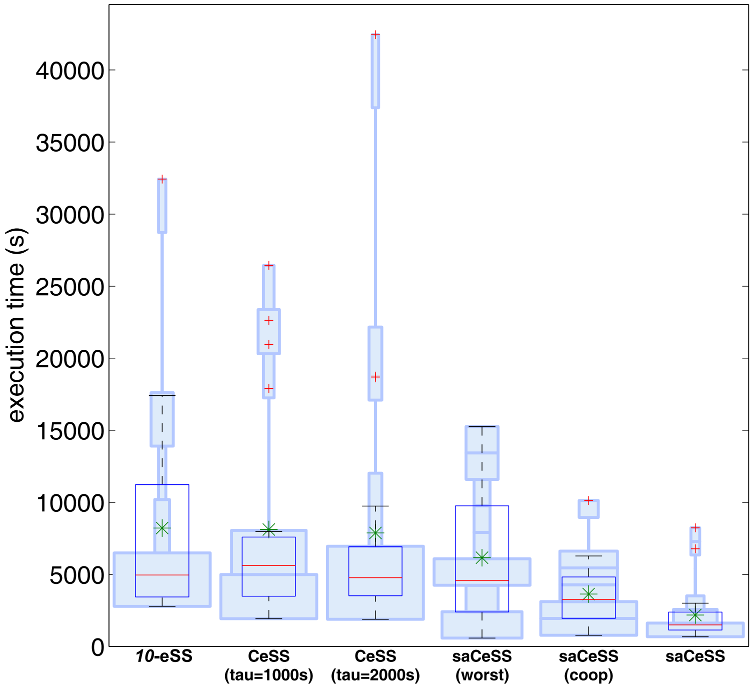

Figure 42: Violin/Box plots of execution time for different strategies in benchmark B6.

Table 7: **Detailed results for benchmark B6**

**method**	**mean iter ±std**	**mean evals ±std**	**mean time ±std(s)**	**min/max time(s)**
10-eSS	4659±3742	9783720±8755231	8217 ± 7536	2779/32429
CeSS ($\tau = 1000s$)	5919±5079	10475485±8978383	8109 ± 7441	1921/26433
CeSS ($\tau = 2000s$)	6108±6850	10778260±12157617	7878 ± 9400	1880/42447
saCeSS(worst)	4243±3210	7383578±5581720	6157 ± 4650	576/15258
saCeSS(coop)	2501±1517	4394243±2689489	3638 ± 2302	771/10122
saCeSS	1500±1265	2594741±2214235	2177 ± 1933	1676/8228

5 Scalability analyses of the proposed saCeSS method

Figures 43, 44, 45 and 46 show the scalability of the proposed saCeSS when the number of processors increases from 1 to 40. They demonstrate that the saCeSS still improves the convergence results when the number of processors grows, thanks to the asynchronous communication protocol.

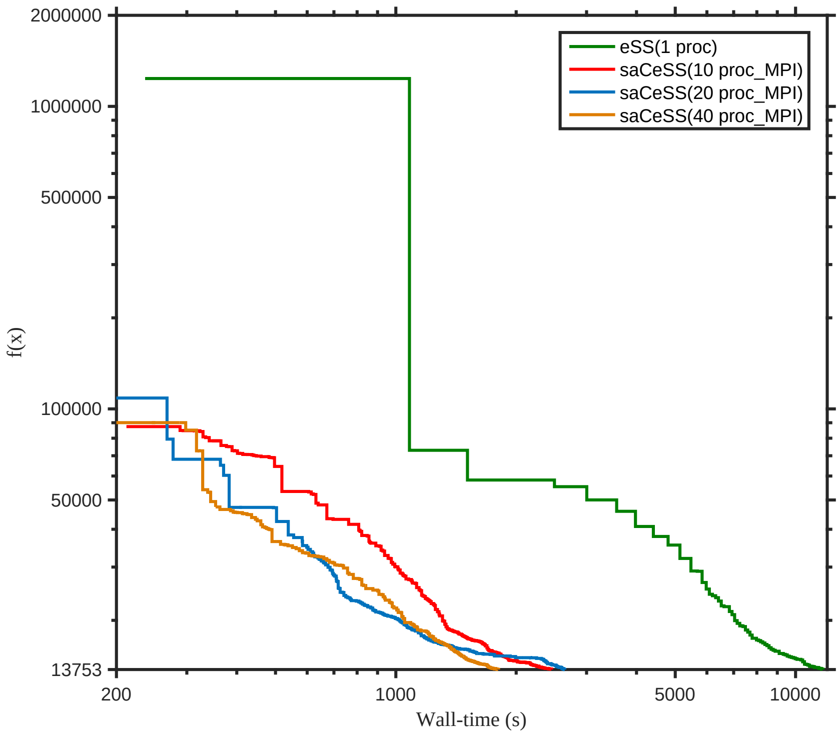

Figure 43: Convergence curves for saCeSS using 1, 10, 20 and 40 processors, corresponding to the runs that are closer to the median values of the results distribution considering benchmark B1.

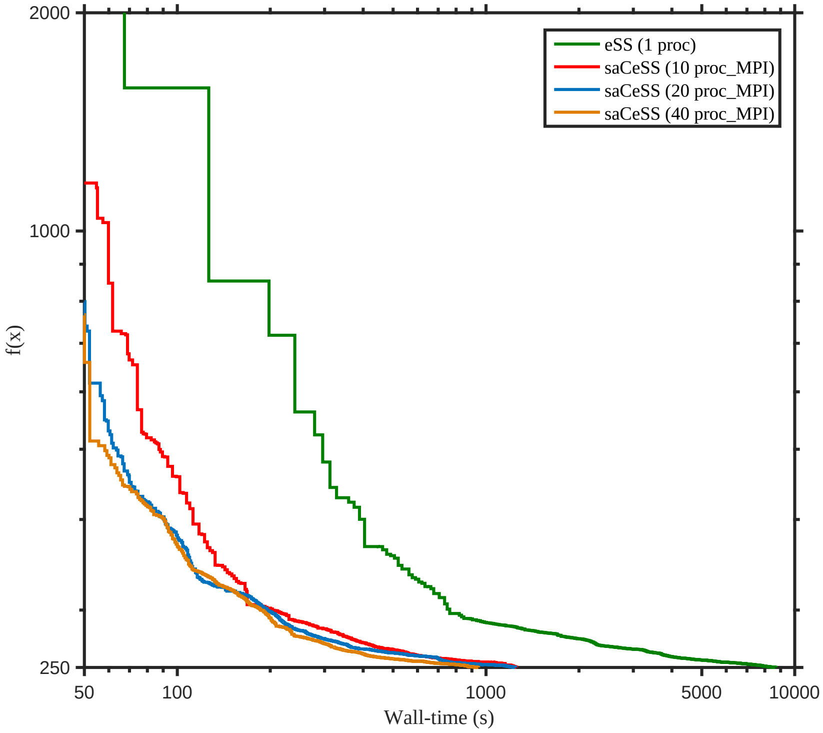

Figure 44: Convergence curves for saCeSS using 1, 10, 20 and 40 processors, corresponding to the runs that are closer to the median values of the results distribution considering benchmark B2.

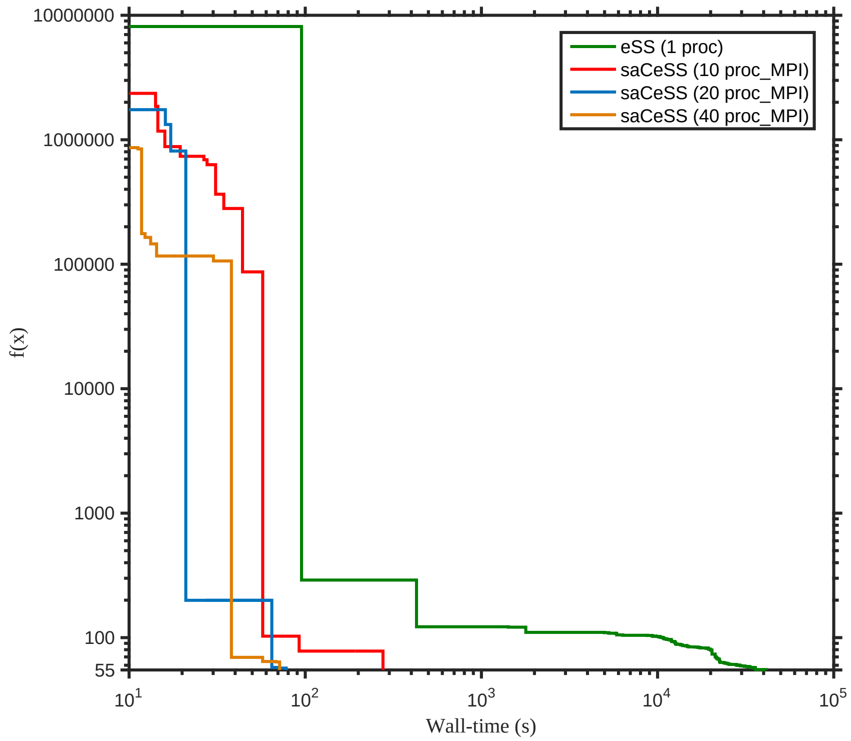

Figure 45: Convergence curves for saCeSS using 1, 10, 20 and 40 processors, corresponding to the runs that are closer to the median values of the results distribution considering benchmark B4.

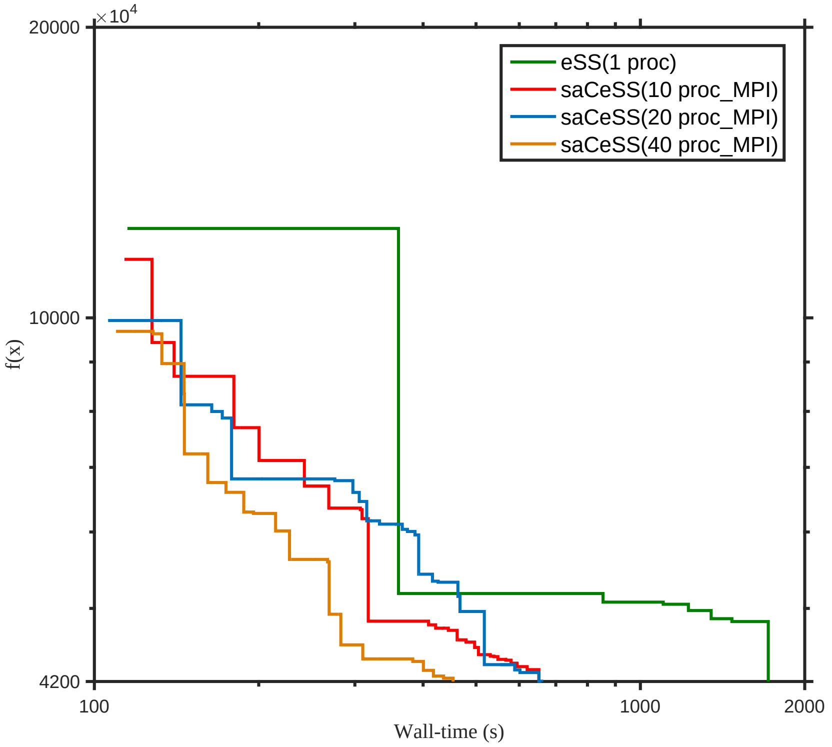

Figure 46: Convergence curves for saCeSS using 1, 10, 20 and 40 processors, corresponding to the runs that are closer to the median values of the results distribution considering benchmark B5.

6 Performance analysis of the hybrid MPI+OpenMP saCeSS implementation

This section shows the performance and scalability of the hybrid MPI+OpenMP implementation proposed in the paper. Benchmarks B3 and B6 were excluded of these evaluations: B3 due to our lack of available resources to run such long executions, and B6 since its currently available implementation could not be carried out with openMP enabled.

Benchmark B1

Figures 47, 48 and 49 allow to analyze the performance of the hybrid MPI+OpenMP saCeSS implementation. For the same number of processors, those hybrid configurations that achieve a good balance between intensification and diversification perform better. Thus, for instance, the configuration of 5 MPI processes with 8 OpenMP threads each performs better than the configuration with 20 MPI processes with 2 OpenMP threads each (see Figure 49).

Figures 50, 51, 52 illustrate the dispersion in the execution time results for these different configurations. Again, in general, those hybrid configurations that achieve a good balance between intensification and diversification obtain a reduction in the variability of the execution time.

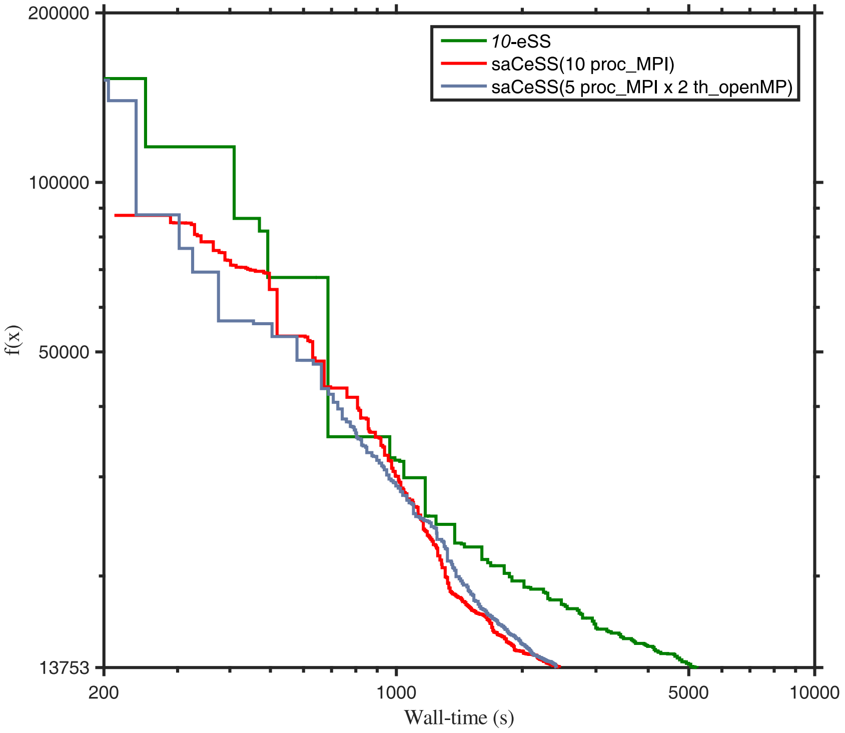

Figure 47: Convergence curves for different configuration of saCeSS using 10 processors, corresponding to the runs that are closer to the median values of the results distribution, for benchmark B1.

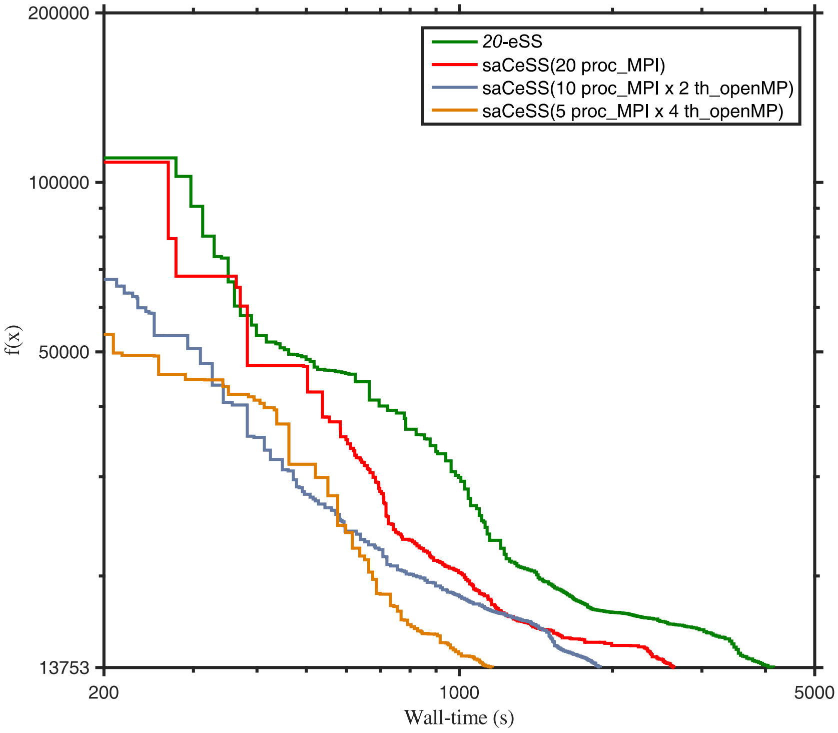

Figure 48: Convergence curves for different configuration of saCeSS using 20 processors, corresponding to the runs that are closer to the median values of the results distribution, for benchmark B1.

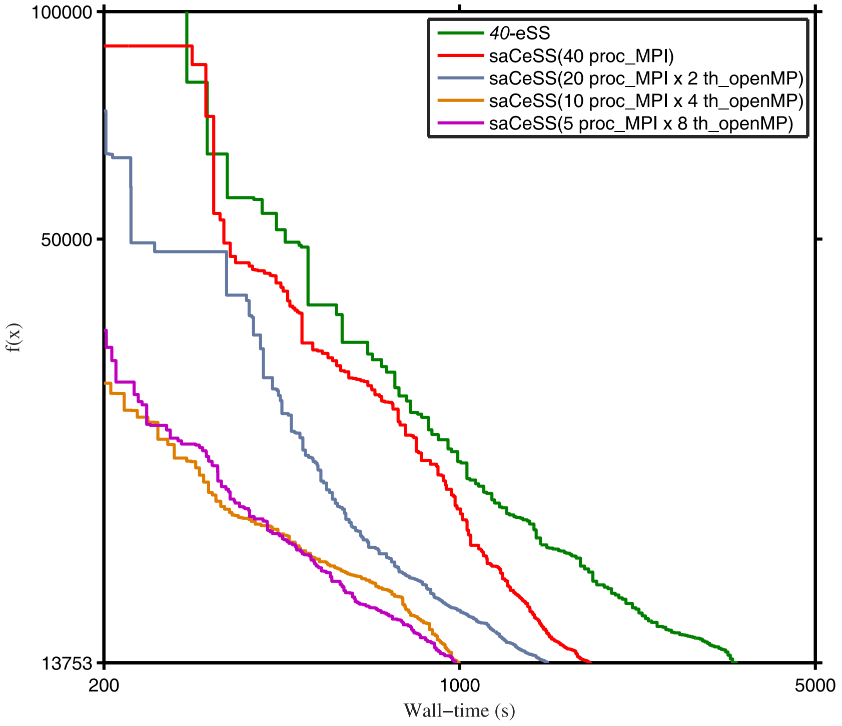

Figure 49: Convergence curves for different configuration of saCeSS using 40 processors, corresponding to the runs that are closer to the median values of the results distribution, for benchmark B1.

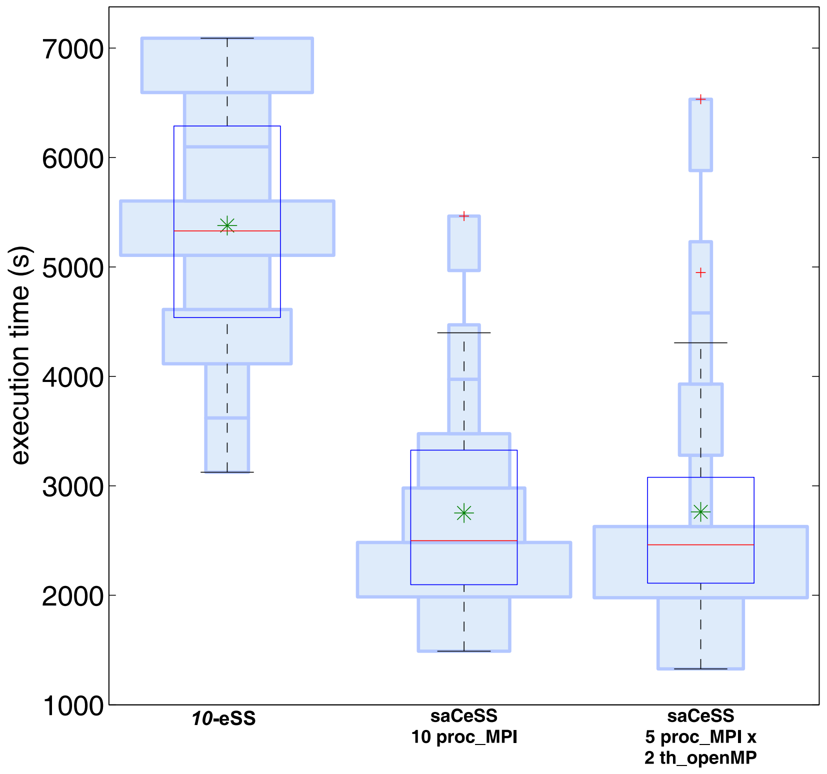

Figure 50: Violin/Box plots of execution time for different configurations of saCeSS using 10 processors considering benchmark B1.

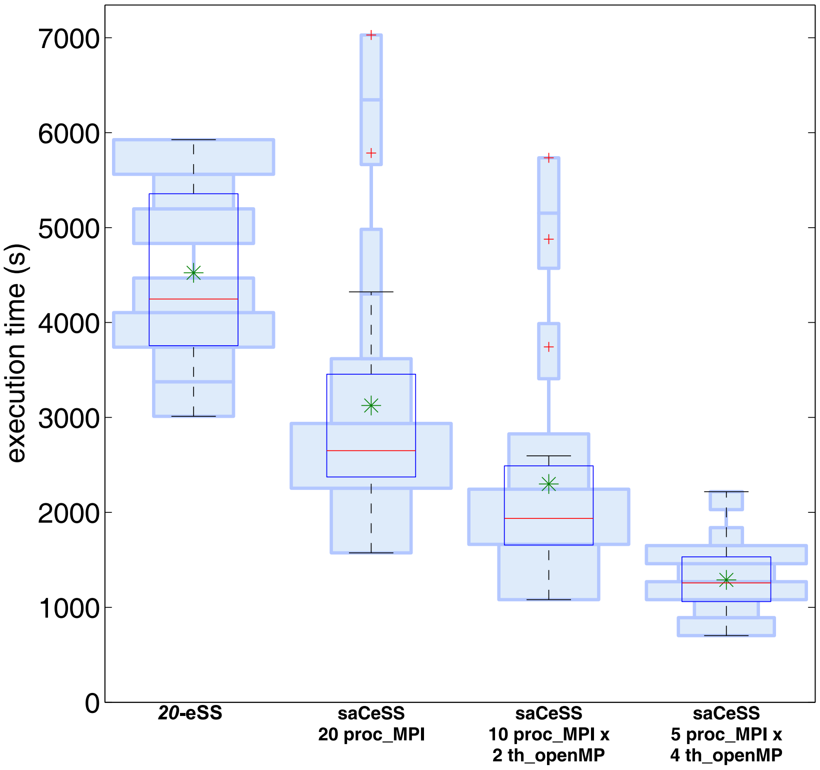

Figure 51: Violin/Box plots of execution time for different configurations of saCeSS using 20 processors considering benchmark B1.

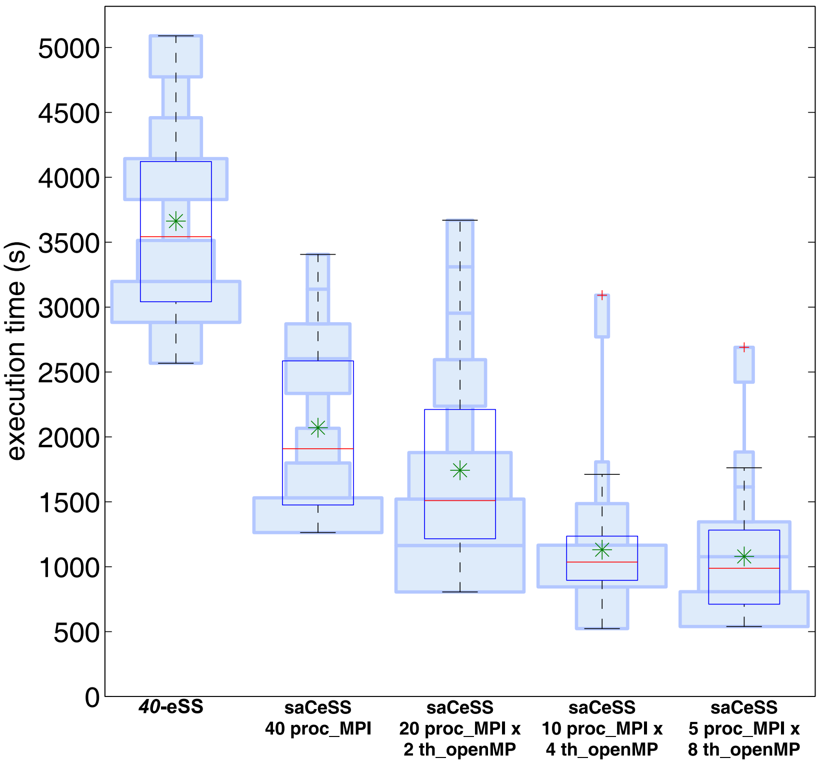

Figure 52: Violin/Box plots of execution time for different configurations of saCeSS using 40 processors considering benchmark B1.

Benchmark B2

Figures 53, 54 and 55 allow to analyze the performance of the hybrid MPI+OpenMP saCeSS implementation. As for benchmark B1, using the same number of processors, those hybrid configurations that achieve a good balance between intensification and diversification perform better. For instance, the configuration of 5 MPI processes with 8 OpenMP threads each performs better than the configuration with 20 MPI processes with 2 OpenMP threads each (see Figure 55).

Figures 56, 57, 58 illustrate the dispersion in the execution time results for these different configurations. Again, achieving a good balance between intensification and diversification is crucial to improve the performance of the method, specially when the number of total processors grows.

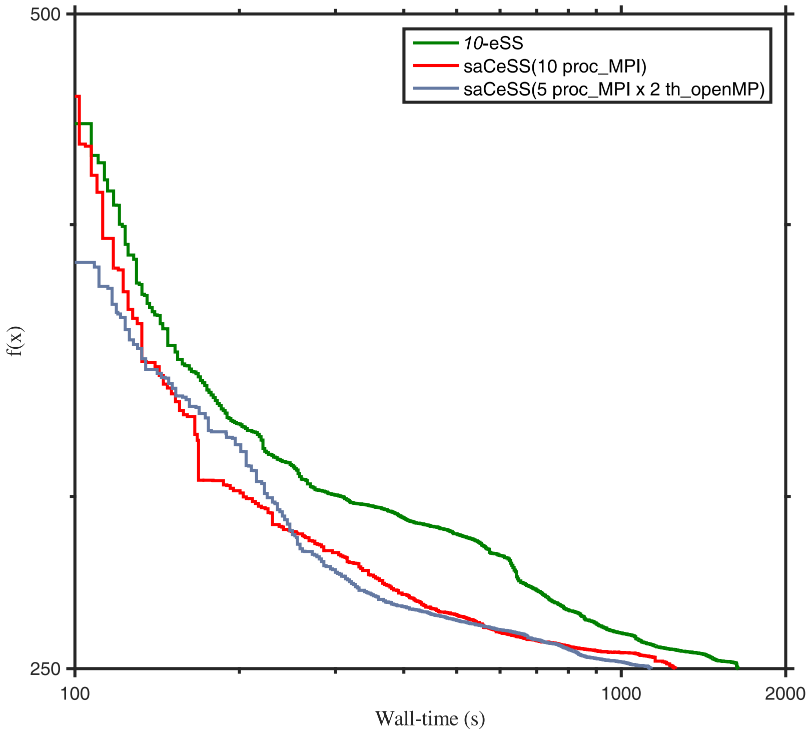

Figure 53: Convergence curves for different configuration of saCeSS using 10 processors, corresponding to the runs that are closer to the median values of the results distribution, for benchmark B2.

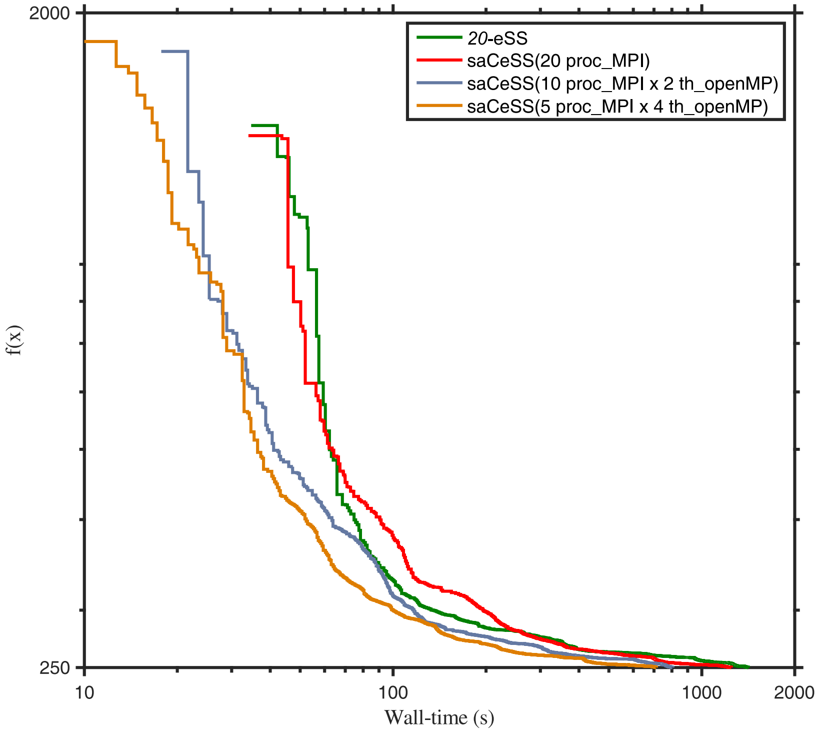

Figure 54: Convergence curves for different configuration of saCeSS using 20 processors, corresponding to the runs that are closer to the median values of the results distribution, for benchmark B2.

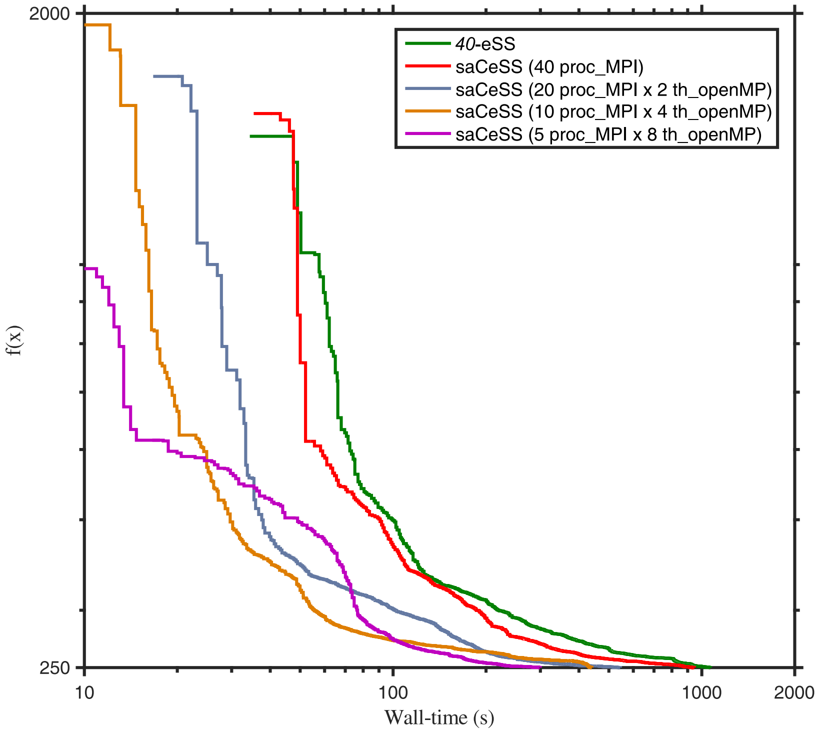

Figure 55: Convergence curves for different configuration of saCeSS using 40 processors, corresponding to the runs that are closer to the median values of the results distribution, for benchmark B2.

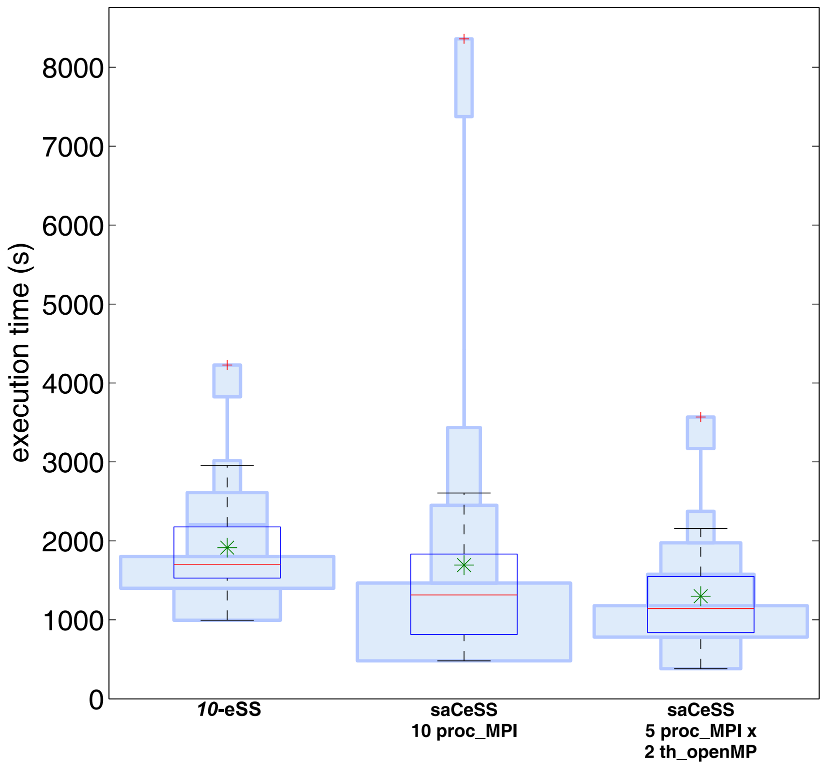

Figure 56: Violin/Box plots of execution time for different configurations of saCeSS using 10 processors considering benchmark B2.

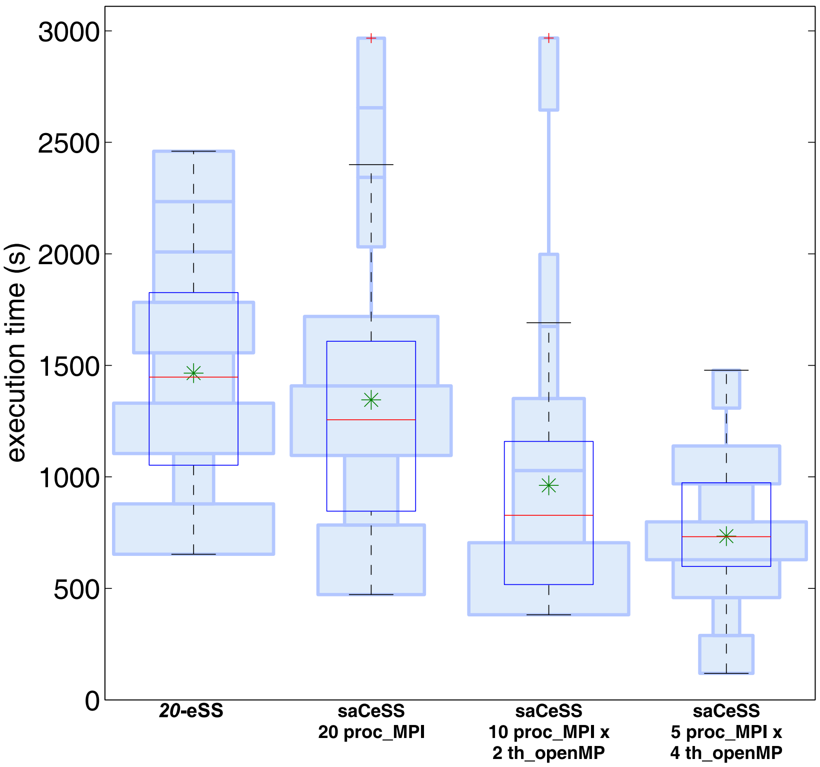

Figure 57: Violin/Box plots of execution time for different configurations of saCeSS using 20 processors considering benchmark B2.

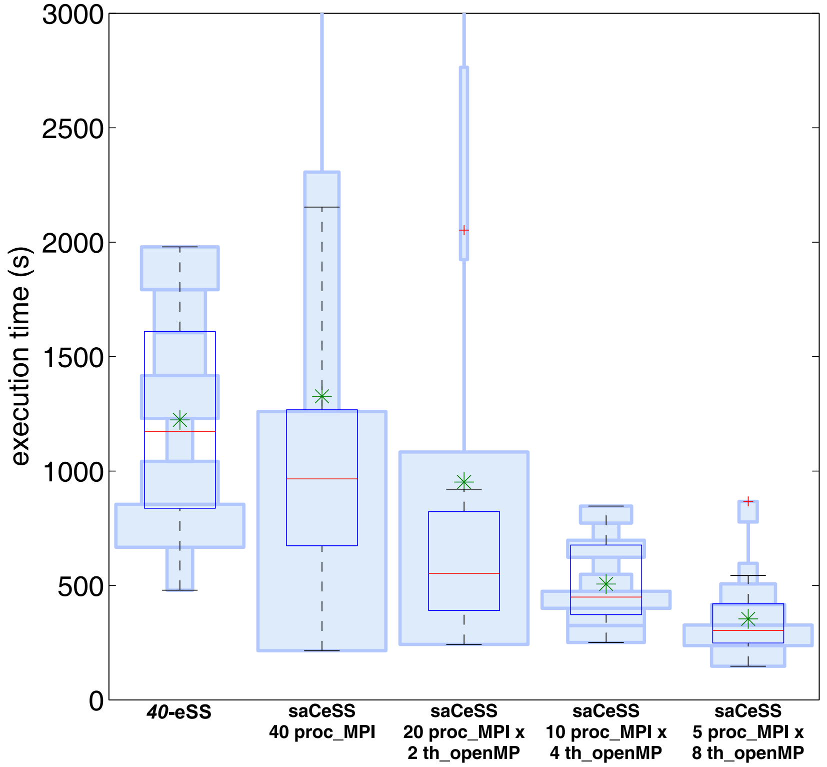

Figure 58: Violin/Box plots of execution time for different configurations of saCeSS using 40 processors considering benchmark B2.

Benchmark B4

Figures 59, 60 and 61 allow to analyze the performance of the hybrid MPI+OpenMP saCeSS implementation. As it can be seen, performance of benchmark B4 is heavily affected by the number of different processes cooperating. That is, this benchmark greatly benefits form the diversity introduced when the number of MPI processes grows versus the intensify in the OpenMP search. Thus, configurations using all the available resources to run MPI cooperative processes outperforms the hybrid configurations.

Figures 62, 63, 64 illustrate the dispersion in the execution time results for these different configurations. Again, it can be seen that, for benchmark B4, the configuration that only uses MPI processes ourperforms the hybrid configurations.

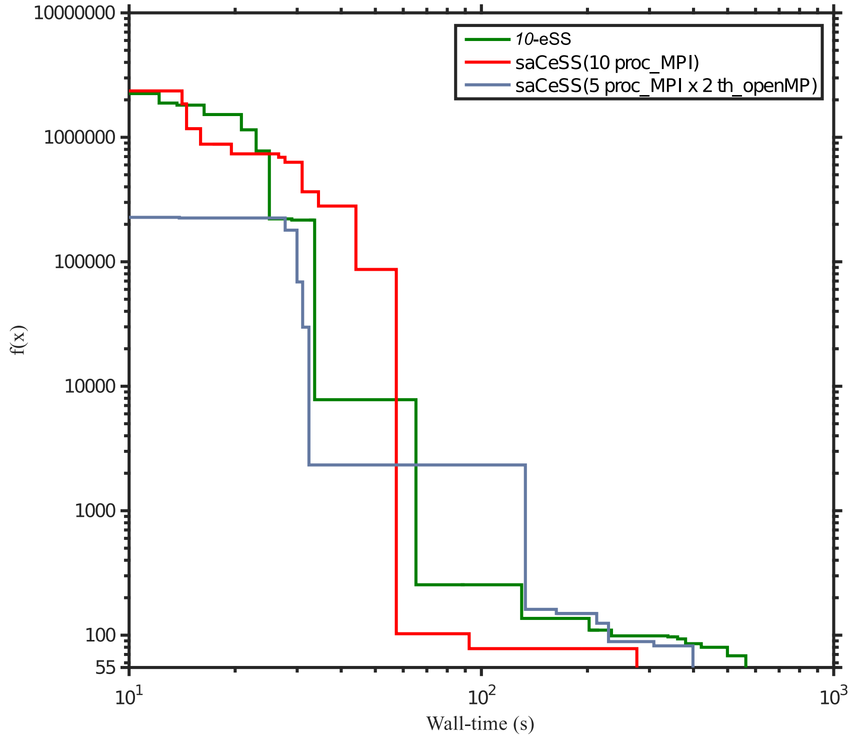

Figure 59: Convergence curves for different configuration of saCeSS using 10 processors, corresponding to the runs that are closer to the median values of the results distribution, for benchmark B4.

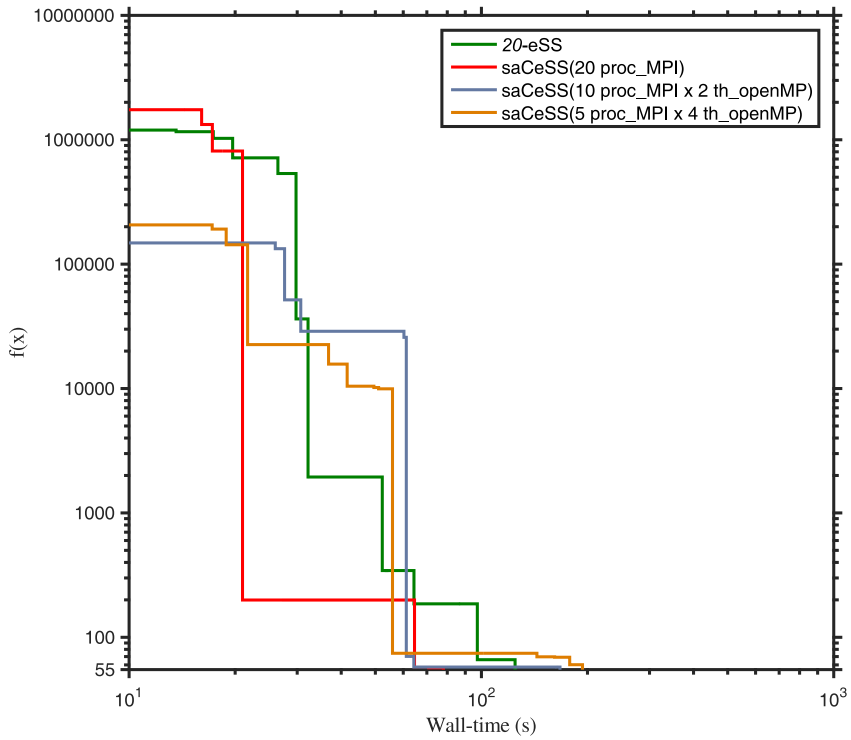

Figure 60: Convergence curves for different configuration of saCeSS using 20 processors, corresponding to the runs that are closer to the median values of the results distribution, for benchmark B4.

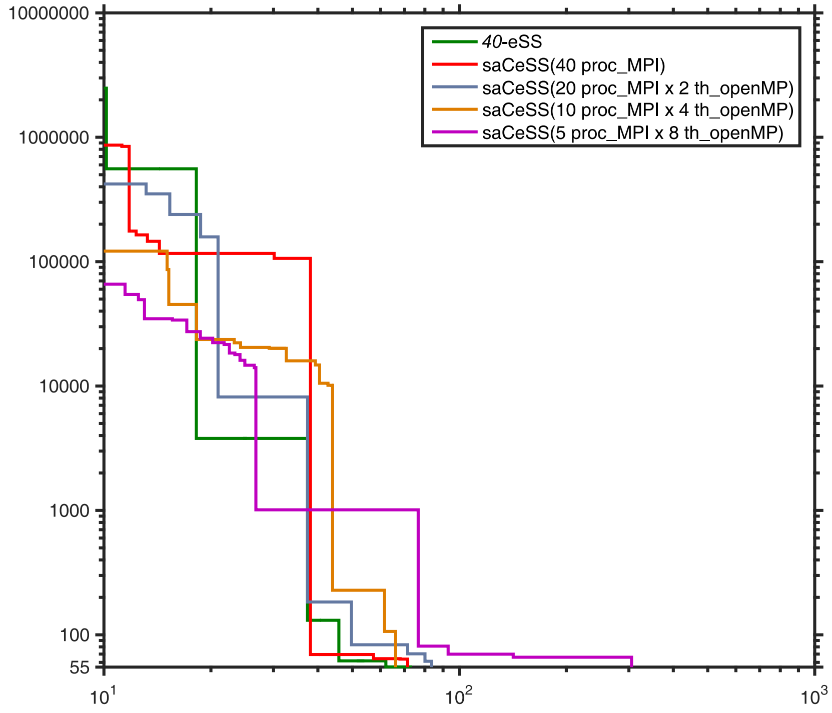

Figure 61: Convergence curves for different configuration of saCeSS using 40 processors, corresponding to the runs that are closer to the median values of the results distribution, for benchmark B4.

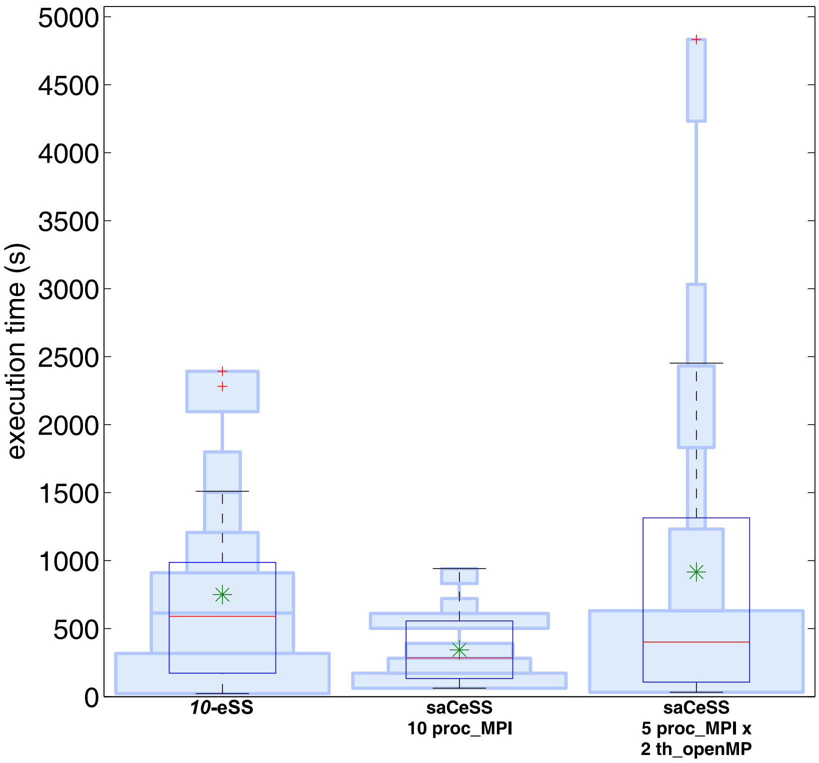

Figure 62: Violin/Box plots of execution time for different configurations of saCeSS using 10 processors considering benchmark B4.

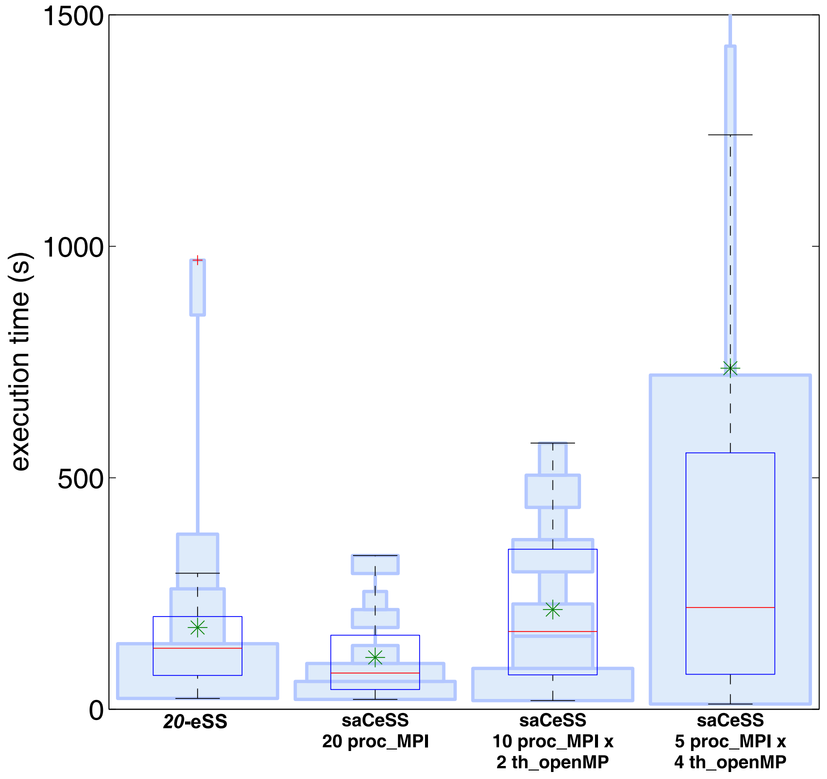

Figure 63: Violin/Box plots of execution time for different configurations of saCeSS using 20 processors considering benchmark B4.

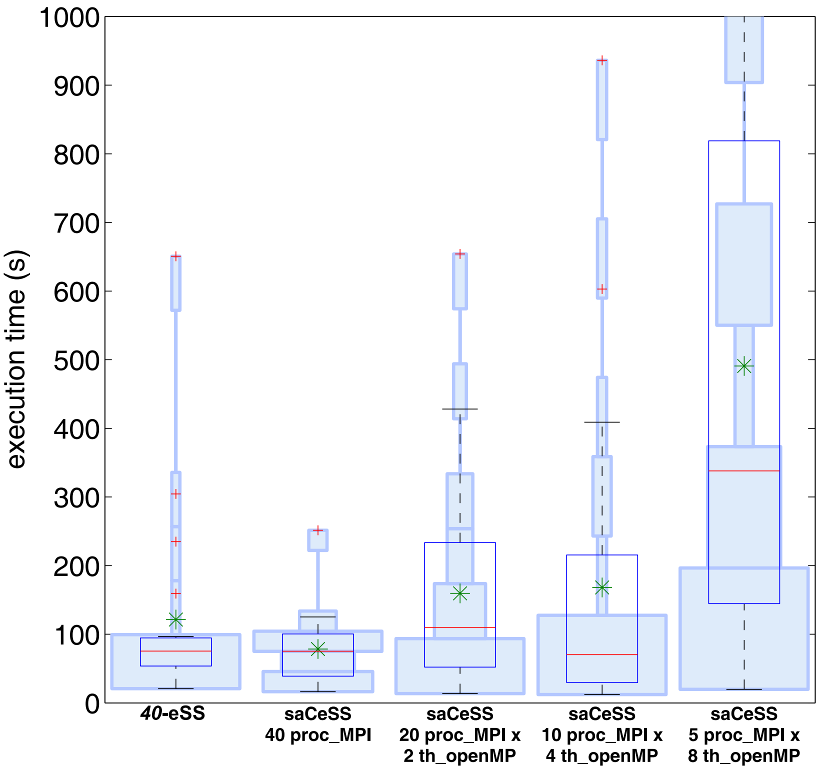

Figure 64: Violin/Box plots of execution time for different configurations of saCeSS using 40 processors considering benchmark B4.

Benchmark B5

Figures 65, 66 and 67 allow to analyze the performance of the hybrid MPI+OpenMP saCeSS implementation. As for benchmarks B1 and B2, when the same number of processors are used, those hybrid configurations that achieve a good balance between intensification and diversification perform better. Thus, the configuration of 5 MPI processes with 4 OpenMP threads each performs better than the configuration with 10 MPI processes with 2 OpenMP threads each (see Figure 66).

Figures 68, 69, 70 illustrate the dispersion in the execution time results for these different configurations. The dispersion, again, is lower in those configurations that balance the number of MPI processes and OpenMP threads.

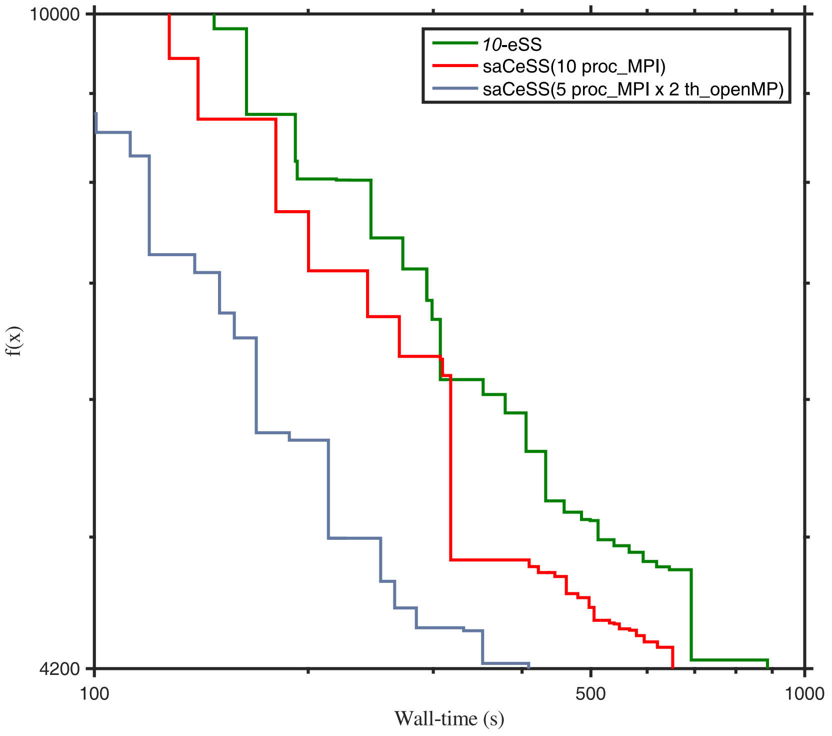

Figure 65: Convergence curves for different configuration of saCeSS using 10 processors, corresponding to the runs that are closer to the median values of the results distribution, for benchmark B5.

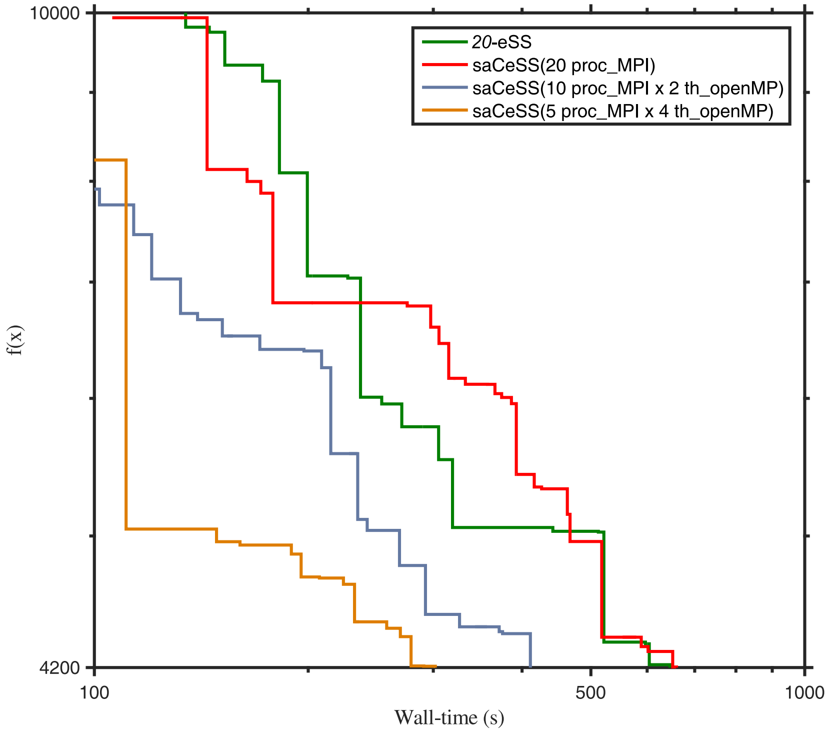

Figure 66: Convergence curves for different configuration of saCeSS using 20 processors, corresponding to the runs that are closer to the median values of the results distribution, for benchmark B5.

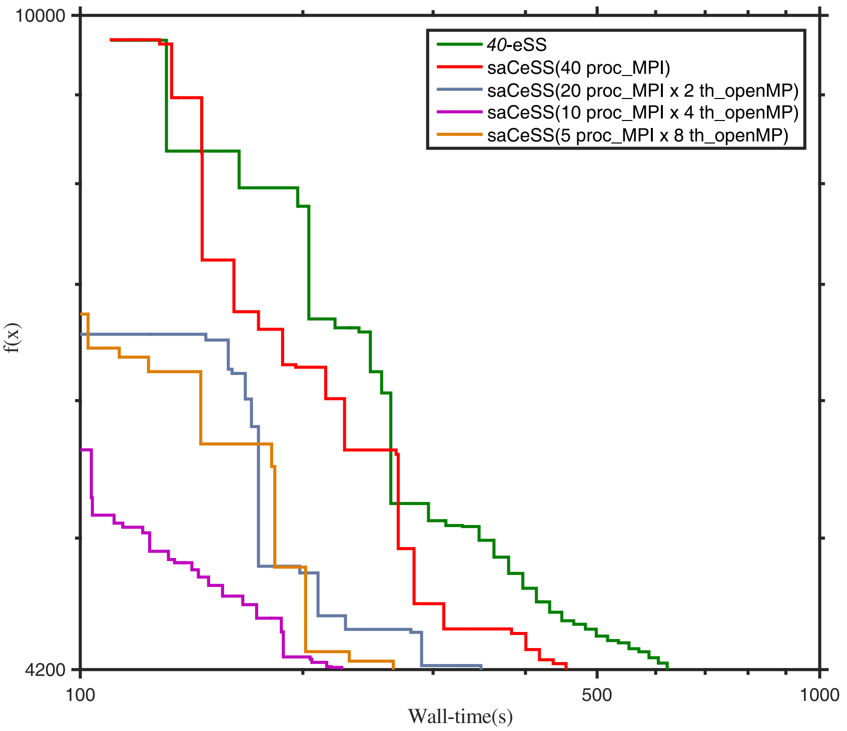

Figure 67: Convergence curves for different configuration of saCeSS using 40 processors, corresponding to the runs that are closer to the median values of the results distribution, for benchmark B5.

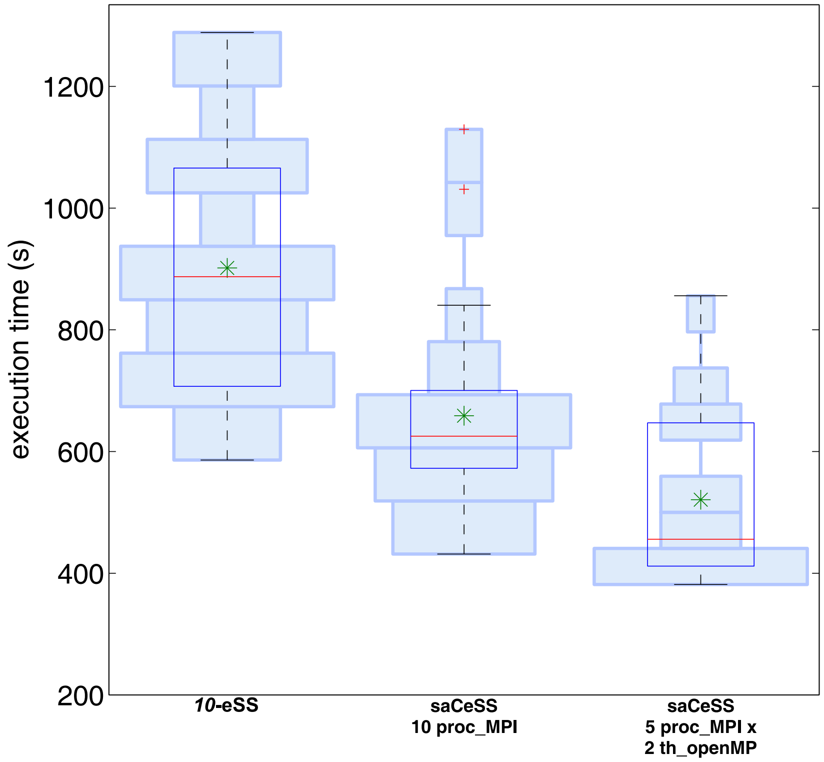

Figure 68: Violin/Box plots of execution time for different configurations of saCeSS using 10 processors considering benchmark B5.

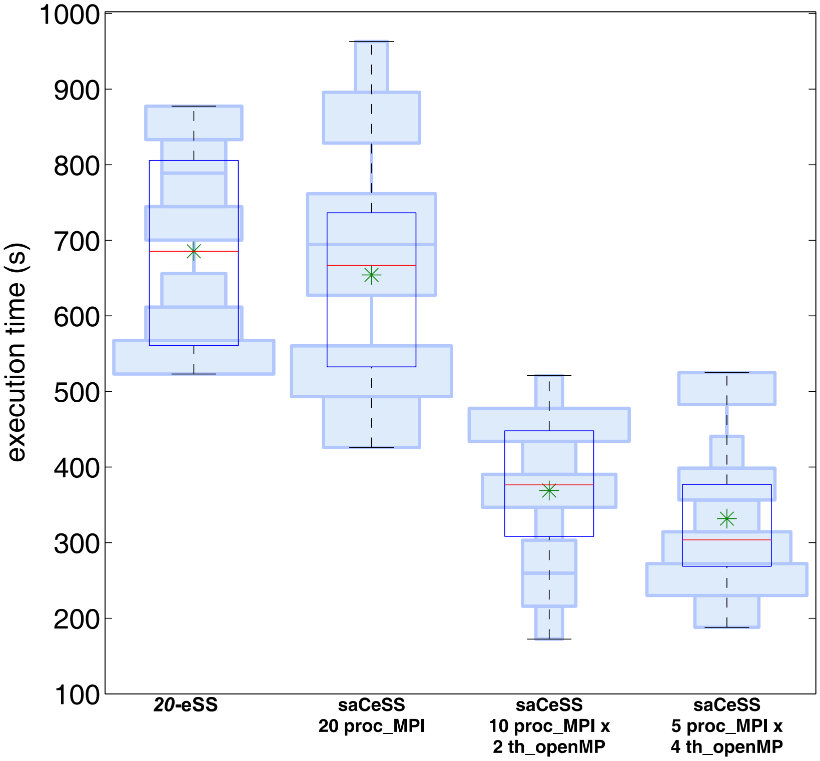

Figure 69: Violin/Box plots of execution time for different configurations of saCeSS using 20 processors considering benchmark B5.

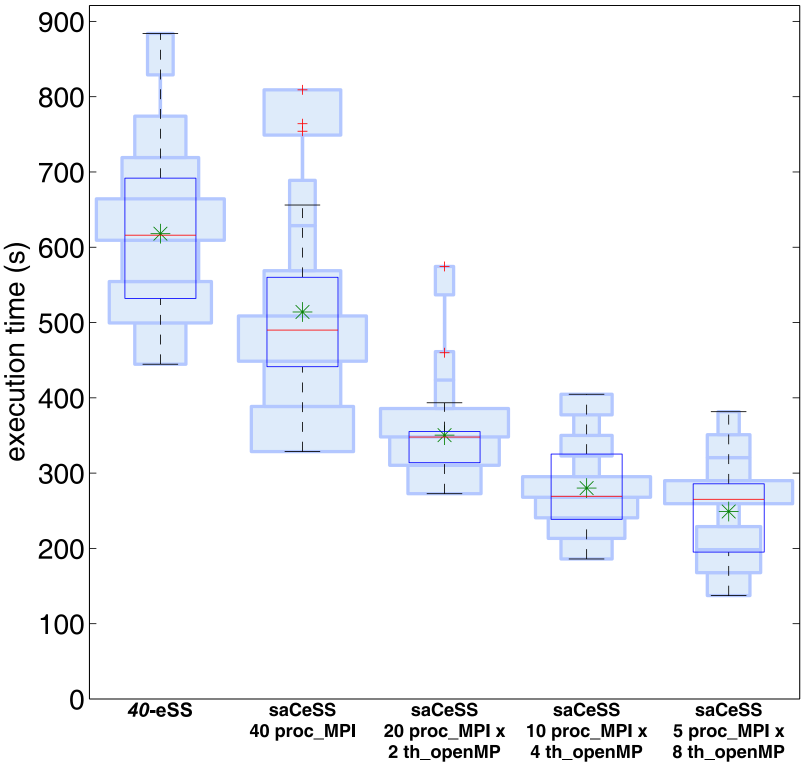

Figure 70: Violin/Box plots of execution time for different configurations of saCeSS using 40 processors considering benchmark B5.

7 Comparative between saCeSS and asynPDE

In order to evaluate the new method with respect to existing parallel metaheuristics, we compare it with another state of the art metaheuristic, Differential Evolution (DE). To ensure a fair comparison, we choose a DE implementation (asynPDE [5]) that improves global search through an asynchronous parallel implementation based on a cooperative island-model, and that also improves the local search by means of several heuristics also employed in the eSS, such as the local solver, the tabu list, or the logarithmic search.

The convergence curves of the asynPDE and the saCeSS algorithms for 10 and 20 processors are shown in the following figures. These figures represent the convergence curves for those experiments that are closer to the median values of the results distribution. Although the best configuration for the saCeSS method is, in general, an hybrid MPI+OpenMP one, since the asynPDE method only performs a coarse-grained parallelization, for comparison purposes the convergence curves of saCeSS using only MPI processes are also shown. As it can be seen in the figures, in all cases the asynPDE algorithm progresses more slowly than the saCeSS method.

Benchmark B1

Figures 71 and 72: comparative analysis between saCeSS and asynPDE with 10 and 20 processors for benchmark B1.

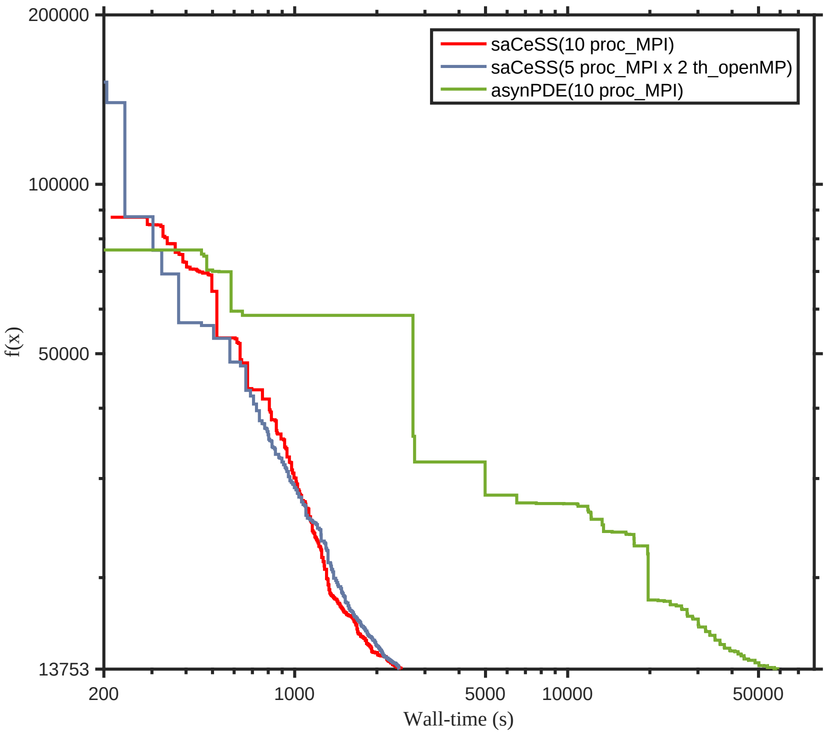

Figure 71: Convergence curves for asynPDE algorithm vs saCeSS using 10 processors considering benchmark B1.

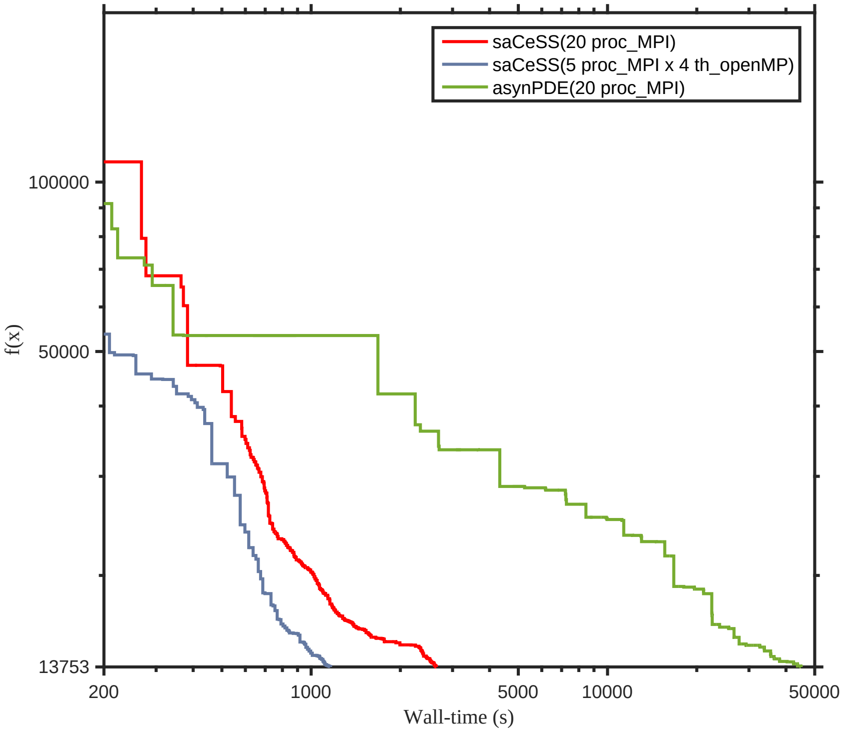

Figure 72: Convergence curves for asynPDE algorithm vs saCeSS using 20 processors considering benchmark B1.

Benchmark B2

Figures 73 and 74 comparative analysis between saCeSS and asynPDE with 10 and 20 processors for benchmark B2.

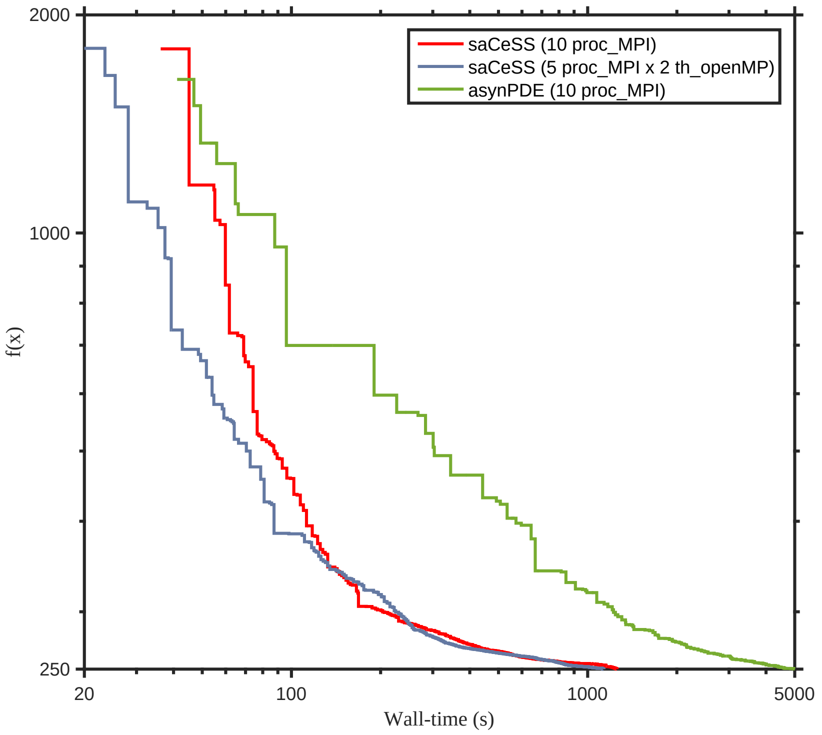

Figure 73: Convergence curves for asynPDE algorithm vs saCeSS using 10 processors considering benchmark B2.

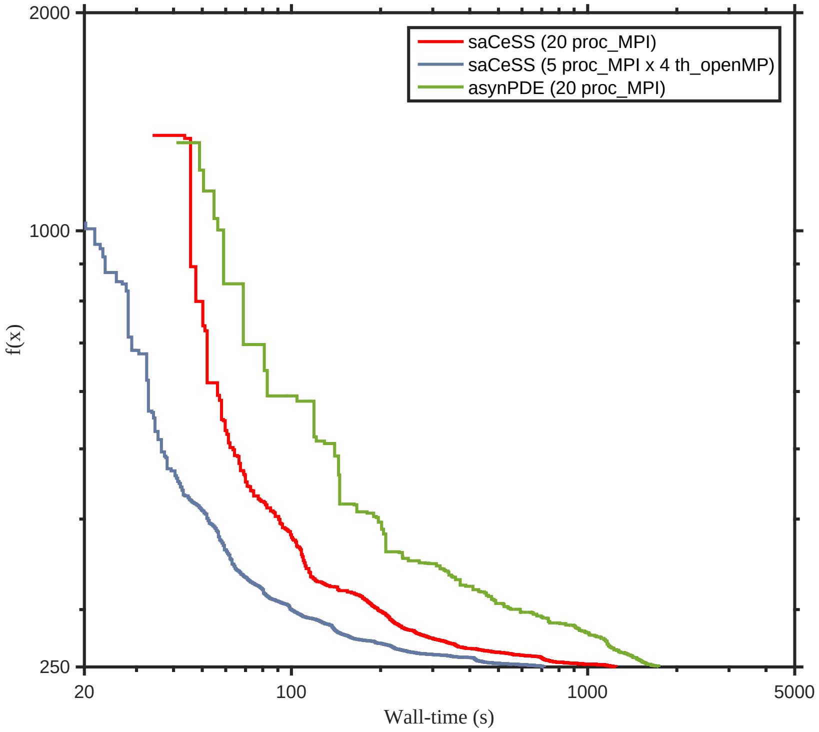

Figure 74: Convergence curves for asynPDE algorithm vs saCeSS using 20 processors considering benchmark B2.

Benchmark B4

The following figures 75 and 76 comparative analysis between saCeSS and asynPDE with 10 and 20 processors for benchmark B4.

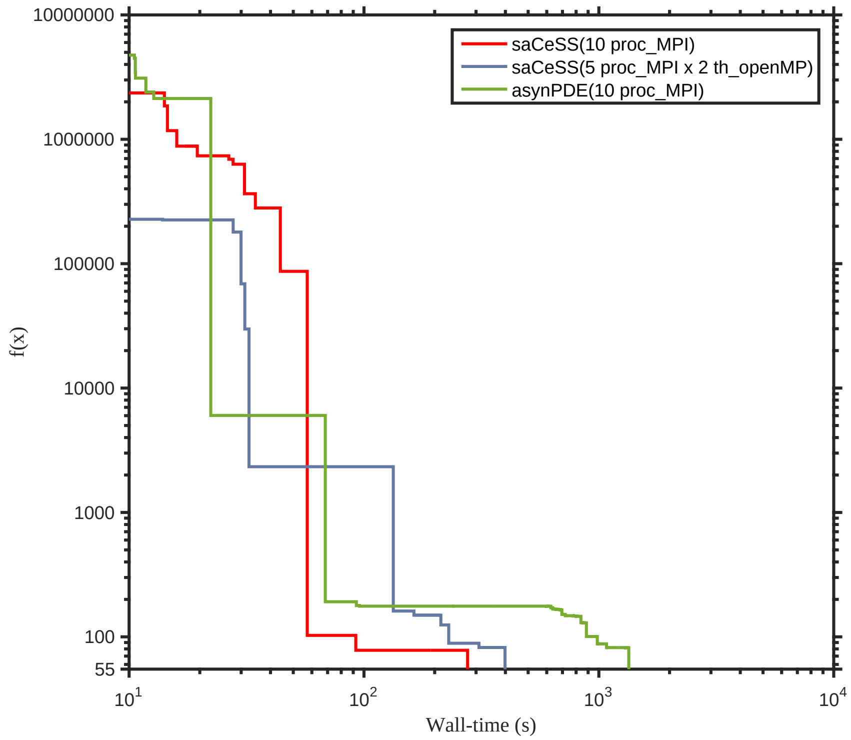

Figure 75: Convergence curves for asynPDE algorithm vs saCeSS using 10 processors considering benchmark B4.

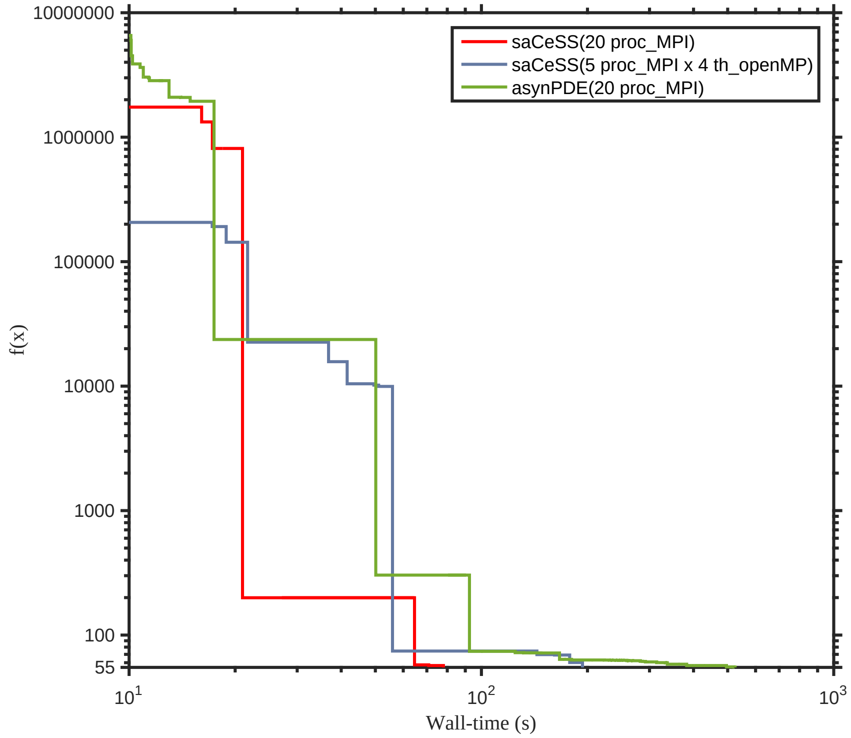

Figure 76: Convergence curves for asynPDE algorithm vs saCeSS using 20 processors considering benchmark B4.

Benchmark B5

Figures 77 and 78 comparative analysis between saCeSS and asynPDE with 10 and 20 processors for benchmark B5.

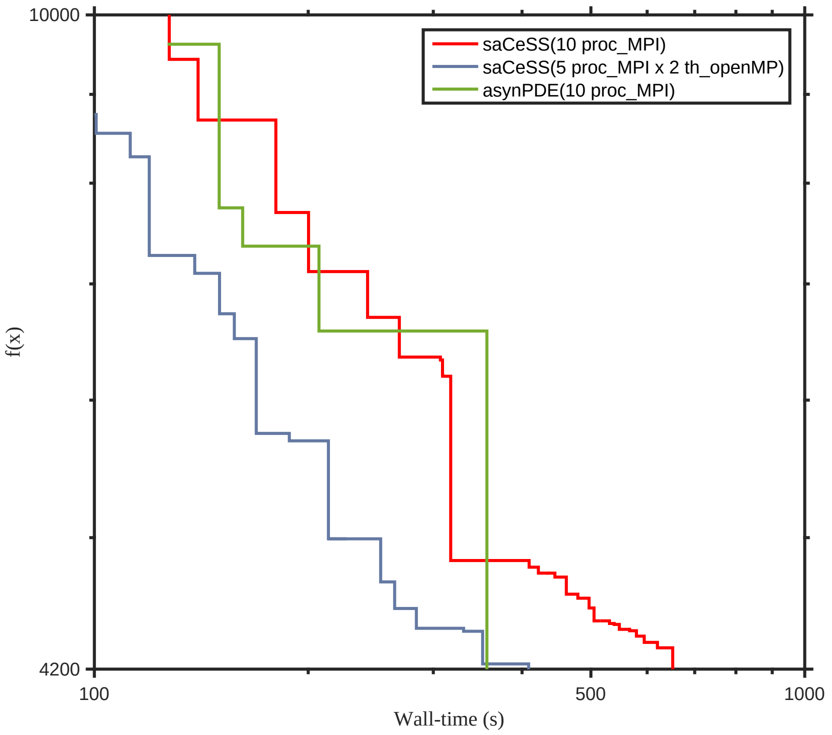

Figure 77: Convergence curves for asynPDE algorithm vs saCeSS using 10 processors considering benchmark B5.

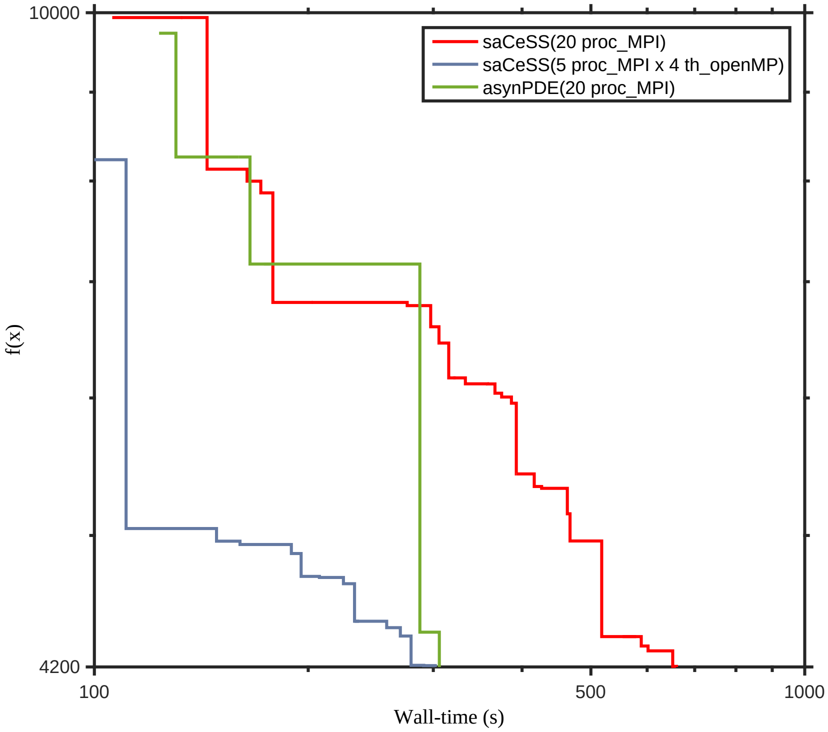

Figure 78: Convergence curves for asynPDE algorithm vs saCeSS using 20 processors considering benchmark B5.

References

[1] Villaverde, A.F., Egea, J.A., Banga, J.R.: A cooperative strategy for parameter estimation in large scale systems biology models. BMC Systems Biology **6:75** (2012)

[2] Villaverde, A.F., Henriques, D., Smallbone, K., Bongard, S., Schmid, J., Cicin-Sain, D., Crombach, A., Saez-Rodriguez, J., Mauch, K., Balsa-Canto, E., Mendes, P., Jaeger, J., Banga, J.R.: Biopredyn-bench: a suite of benchmark problems for dynamic modelling in systems biology. BMC Systems Biology **9:1-15** (2015). In press

[3] Dennis, J.E. Jr., Gay, D.M., Welsch, R.E.: Algorithm 573: Nl2sol - an adaptive nonlinear least-squares algorithm. ACM Trans. Math. Softw. **7**(3), 369–383 (1981)

[4] de la Maza, M., Yuret, D.: Dynamic hill climbing. AI Expert **9**(3), 26–31 (1994)

[5] Penas, D., Banga, J., González, P., Doallo, R.: Enhanced parallel differential evolution algorithm for problems in computational systems biology. Applied Soft Computing **33**, 86–99 (2015)
